# Hurdles in the evolutionary epidemiology of *Angiostrongylus cantonensis*: Pseudogenes, incongruence between taxonomy and DNA sequence variants, and cryptic lineages

**DOI:** 10.1111/eva.12621

**Published:** 2018-03-25

**Authors:** Sirilak Dusitsittipon, Charles D. Criscione, Serge Morand, Chalit Komalamisra, Urusa Thaenkham

**Affiliations:** ^1^ Department of Helminthology Faculty of Tropical Medicine Mahidol University Bangkok Thailand; ^2^ Departments of Parasitology and Entomology Faculty of Public Health Mahidol University Bangkok Thailand; ^3^ Department of Biology Texas A&M University College Station TX USA; ^4^ CNRS ISEM‐CIRAD ASTRE Faculty of Veterinary Medicine Kasetsart University Bangkok Thailand; ^5^ Mahidol‐Bangkok School of Tropical Medicine Faculty of Tropical Medicine Mahidol University Bangkok Thailand

**Keywords:** *Angiostrongylus cantonensis*, *Angiostrongylus mackerrasae*, *Angiostrongylus malaysiensis*, cryptic lineages, *cytochrome b* (*CYTB*) gene, *cytochrome c oxidase* 1 (*CO*1) gene, pseudogene, species complex

## Abstract

*Angiostrongylus cantonensis*, the rat lungworm, is a zoonotic pathogen that is one of the leading causes of eosinophilic meningitis worldwide. This parasite is regarded as an emerging pathogen with a global range expansion out of southeastern Asia post‐WWII. To date, molecular systematic/phylogeographic studies on *A. cantonensis* have mainly used two mitochondrial (mtDNA) markers, *cytochrome c oxidase 1* (*CO*1) and *cytochrome b* (*CYTB*), where the focus has largely been descriptive in terms of reporting local patterns of haplotype variants. In order to look for more global evolutionary patterns, we herein provide a collective phylogenetic assessment using the six available whole mtDNA genome samples that have been tagged as *A. cantonensis*,* A. malaysiensis*, or *A. mackerrasae* along with all other GenBank *CO*1 and *CYTB* partial sequences that carry these species identifiers. The results reveal three important complications that researchers will need to be aware of, or will need to resolve, prior to conducting future molecular evolutionary studies on *A. cantonensis*. These three problems are (i) incongruence between taxonomic identifications and mtDNA variants (haplotypes or whole mtDNA genome samples), (ii) the presence of a *CYTB* mtDNA pseudogene, and (iii) the need to verify *A. mackerrasae* as a species along with other possible cryptic lineages, of which there is suggestive evidence (i.e., *A. cantonensis* could be a species complex). We provided a discussion of how these complications are hurdles to our understanding of the global epidemiology of angiostrongyliasis. We call for future studies to be more explicit in morphological traits used for identifications (e.g., provide measurements). Moreover, it will be necessary to repeat prior morphological and life‐history studies while simultaneously using sequence data in order to assess possible associations between critical epidemiological data (e.g., biogeography, virulence/pathology, host species use) and specific lineages.

## 1 INTRODUCTION

*Angiostrongylus cantonensis*, the rat lungworm, is a zoonotic pathogen that is one of the leading causes of eosinophilic meningitis worldwide (Barratt et al., 2016). This parasite is regarded as an emerging pathogen with a global range expansion out of southeastern Asia post‐WWII (Kliks & Palumbo, 1992; Wang, Lai, Zhu, Chen, & Lun, 2008). Despite cases of human infection in many of the areas where *A. cantonensis* has expanded its range (Wang et al., 2008), there has been little attempt to use molecular evolutionary methods to elucidate the global epidemiology of this important pathogen. Indeed, the use of DNA data only came about in the 2000s. For example, ribosomal DNA sequences were used to assess metastrongylid nematode relationships (Carreno & Nadler, 2003) or to help survey larvae from mollusk intermediate hosts (Fontanilla & Wade, 2008; Qvarnstrom, Sullivan, Bishop, Hollingsworth, & da Silva, 2007; Qvarnstrom et al., 2010), and ~360‐bp region of the *cytochrome c oxidase 1* (*CO*1) gene was used to assess relationships to other species of *Angiostrongylus* (Eamsobhana et al., 2010). To date, molecular systematic/phylogeographic studies on *A. cantonensis* have mainly used two mitochondrial (mtDNA) markers, *CO*1 and *cytochrome b* (*CYTB*), where the focus has largely been descriptive in terms of reporting local patterns of haplotype variants (Aghazadeh et al., 2015; Dalton, Fenton, Cleveland, Elsmo, & Yabsley, 2017; Dusitsittipon, Criscione, Morand, Komalamisra, & Thaenkham, 2017; Dusitsittipon, Thaenkham, Watthanakulpanich, Adisakwattana, & Komalamisra, 2015; Eamsobhana, Song, et al., 2017; Eamsobhana, Yong, et al., 2017; Lv et al., 2012; Monte et al., 2012; Moreira et al., 2013; Nakaya et al., 2013; Okano et al., 2014; Rodpai et al., 2016; Simoes et al., 2011; Tokiwa et al., 2012, 2013; Vitta et al., 2016; Yong, Eamsobhana, Song, Prasartvit, & Lim, 2015; Yong, Song, Eamsobhana, Goh, & Lim, 2015; Yong, Song, Eamsobhana, & Lim, 2016).

Comparisons among molecular systematic studies on *A. cantonensis* would be useful to look for evolutionary patterns that could aid in epidemiological inference (e.g., biogeographic patterns and sources of introductions, host‐associated or disease‐associated lineages). Unfortunately, because studies did not concurrently use the *CO*1 and *CYTB* markers, such comparisons are difficult to make. However, the availability of recent whole mitochondrial genome sequences of *A. cantonensis*,* A. malaysiensis*, and *A. mackerrasae* provides a chance to “anchor” *CO*1 and *CYTB* clades because the mtDNA does not generally recombine (Ballard & Whitlock, 2004).

In order to look for more global evolutionary patterns, we herein provide a collective phylogenetic assessment using the six available whole mtDNA genome samples that have been tagged as *A. cantonensis*,* A. malaysiensis*, or *A. mackerrasae* along with all other GenBank *CO*1 and *CYTB* partial sequences that carry these species identifiers. Our study focuses on these three species due to their morphological and life‐history similarities (Spratt, 2015; details given in the Discussion). Our results reveal three important complications that researchers will need to be aware of, or will need to resolve, prior to conducting future molecular evolutionary studies on *A. cantonensis*. These three problems are (i) incongruence between taxonomic identifications and mtDNA variants (haplotypes or whole mtDNA genome samples), (ii) the presence of a *CYTB* mtDNA pseudogene, and (iii) the need to verify *A. mackerrasae* as a species along with other possible cryptic lineages, of which there is suggestive evidence (i.e., *A. cantonensis* could be a species complex). We provided a discussion of how these complications are hurdles to our understanding of the global epidemiology of angiostrongyliasis. We call for future studies to be more explicit in morphological traits used for identifications (e.g., provide measurements). Moreover, it will be necessary to repeat prior morphological and life‐history studies while simultaneously using sequence data in order to assess possible associations between critical epidemiological data (e.g., biogeography, virulence/pathology, host species use) and specific lineages.

## 2 MATERIALS AND METHODS

### 2.1 Assembly of *CYTB* and *CO*1 datasets

Available in GenBank, there are five mitochondrial genomes identified as either *A. cantonensis* (NC_013065 = GQ398121, Lv et al., 2012; AP017672, BioProject: PRJEB493; KT186242, Yong, Song, et al., 2015; KT947978, Yong et al., 2016) or *A. malaysiensis* (KT947979 = NC_030332; Yong et al., 2016). A whole mtDNA genome identified as *A. mackerrasae* was discussed in Aghazadeh et al. (2015), but where the actual sequence was obtained from the appendix of the thesis by Aghazadeh (2015). We refer to these six samples as the mtDNA genome reference samples. Sample information, for example, geographic location, and host species, of the six reference samples was obtained from the original publications or available information on GenBank and is included in Tables S1 and S2.

As prior studies on *A. cantonensis* do not include both *CO*1 and *CYTB*, a primary goal was to determine the correspondence between *CO*1 and *CYTB* clades that have been identified in prior work. Because the mtDNA does not generally recombine (Ballard & Whitlock, 2004), we used the *CYTB* and *CO*1 sequences from the above six mtDNA genome reference samples to anchor *CYTB* or *CO*1 haplotypes obtained from previous studies. From GenBank, we assembled all available *CYTB* or *CO*1 sequences that have been tagged as *A. cantonensis*,* A. malaysiensis*, or *A. mackerrasae* and that have associated publications. Tables S1 and S2 also contain the sample information for all these additional sequences obtained from GenBank. For outgroups, we used *CYTB* and *CO*1 sequences from the mtDNA genome samples of *A. costaricensis* (NC013067, AP017675, and KR827449) and *A. vasorum* (NC_018602).

Alignments were conducted with ClustalW in BioEdit (Hall, 1999) along with manual corrections made by eye. In aligning sequences, a haplotype from a particular study may have been subsumed (i.e., there was an identical match in the overlapping region) by a longer sequence from another study. In such cases, we only used the longer sequence in the phylogenetic analyses. The matching GenBank haplotypes are indicated in Tables S1 and S2.

The *CYTB* alignment consisted of approximately 852 nucleotides. Primers that amplify this region of *CYTB* are described in Dusitsittipon et al. (2017) (also, see below). Due to the presence of indels in a *CYTB* pseudogene (see Results), the *CYTB* alignment was based on amino acid translation (translation table 5, the invertebrate mtDNA code, on GenBank) of the mtDNA sequences. Subsequent nucleotide alignments (after back translation) were then kept in codon reading frame to facilitate downstream molecular evolution analyses. The *CYTB* dataset contained 38 unique in‐group haplotypes (including pseudogene haplotypes; see Results) and three outgroup sequences. Table S3 contains the *CYTB* alignment in fasta format.

Because many of the available *CO*1 sequences were short (e.g., ~300 bases), information with regard to the relationships among the six mtDNA genome reference samples would be lost if we trimmed the alignment to match the shortest sequence. As our primary interest was in assessing *CO*1 haplotype relationships with respect to the six mtDNA genome reference samples, the *CO*1 alignment retained the full‐length sequences of reference samples. The resulting gapped sites (i.e., sites that flank the overlapping region) in such an alignment are treated as missing data in a maximum‐likelihood phylogenetic analysis. We note that there were two *CO*1 sequences (MF000735 and MF000736) from Dalton et al. (2017) that did not overlap with the region of *CO*1 that has predominately been used in studies of *A. cantonensis*. As a result, the phylogenetic positions of the *CO*1 haplotypes of Dalton et al. (2017) should only be interpreted with respect to the six reference sequences and not the remaining *CO*1 haplotypes. The topology of the *CO*1 tree remains the same even if the sequences of Dalton et al. (2017) are excluded (results not shown). The *CO*1 dataset contained 41 unique in‐group haplotypes and four outgroup sequences. Table S4 contains the *CO*1 alignment in fasta format. Tables S1 and S2 contain information on the references from which *CO*1 or *CYTB* haplotypes originated as well as matching haplotypes, naming conventions, and sample identifiers used in the original papers.

### 2.2 Phylogenetic analyses

Phylogenetic analyses were conducted in MEGA6 software (Tamura, Stecher, Peterson, Filipski, & Kumar, 2013) after using the maximum‐likelihood model selection (with the “use all sites' option”) to determine the evolutionary substitution model. To confirm that both the *CO*1 and *CYTB* haplotypes from the six reference samples provided the same phylogenetic signal, we first conducted maximum‐likelihood analyses using the TN93 + G and HKY + G models, respectively. For heuristic purposes and to determine whether the two mtDNA markers provided similar information, we calculated pairwise *p*‐distance between the six reference samples and tested for a correlation between the *CO*1 and *CYTB* pairwise *p*‐distances using a Mantel test. For the complete *CO*1 and *CYTB* datasets, maximum‐likelihood analyses with the GTR + G and HKY + G models, respectively, were used for phylogenetic reconstruction. One thousand bootstraps were used to assess nodal support. In our prior study (Dusitsittipon et al., 2017), we found strong associations between nine nuclear microsatellite clusters and *CYTB* mtDNA clades. We follow the naming conventions of the clusters and clades identified in Dusitsittipon et al. (2017) to facilitate discussion in linking to *CO*1 clades.

In general, we linked *CO*1 and *CYTB* clades based on the phylogenetic positions of the reference samples. However, bootstrap support was expected to be low in the complete *CO*1 dataset because of the presence of many short sequences (e.g., ~300 bases). As a result, it is important to recognize that the positions of the short *CO*1 haplotypes are subject to change pending acquisition of new sequence data. Also, for the latter reason, we used a conservative approach where a link in the *CO*1 phylogeny was made only back to the node that contained the most recent common ancestor of a reference sequence unless a clade had >90% bootstrap support. There were two exceptions to this rule where we used the relative phylogenetic positions of clades; we consider these exceptions to be hypothesized clades (see Results).

### 2.3 Pseudogene analyses

In our prior study (Dusitsittipon et al., 2017), we described how the *CYTB* primer pairs *Cytb*‐F, 5′‐TGAATAGACAGAATTTTAAGAG‐3′ and *Cytb*‐R, 5′‐ATCAACTTAACATTACAGAAAC‐3′ of Dusitsittipon et al. (2015) would fail in some samples or produce sequences that translated with premature stop codons (henceforth referred to as untranslatable haplotypes). Upon the design of new primers *Cytb*‐F3 5′‐ATGTTATCTTGATAAGGTAGAG‐3′ and *Cytb*‐R2 5′‐AACCTAAATTCAATATACATACTAT‐3′ (see Dusitsittipon et al., 2017), we were able to obtain translatable sequences from specimens that previously failed or produced untranslatable haplotypes. Specimen sampling and sequencing protocols are explained in detail in Dusitsittipon et al. (2017). Here, we note that untranslatable haplotypes were obtained from 41 individuals (Table S1 gives the individual identifiers and locations of samples) using *Cytb*‐F and *Cytb*‐R. From 30 of these 41 individuals, we also obtained the real *CYTB* sequence using *Cytb*‐F3 and *Cytb*‐R2 (Table 1).

**Table 1:** Samples from which both the pseudogene and *CYTB* gene were obtained (*n* = 30)

Sample ID	Provinces	Coordinates	Pseudogene haplotype	*CYTB* haplotype
BKK 6, 7 BKK 9	Bangkok	13°45′0″N 100°31′1.20″E	H22 H24	H14 H14
LB 19–21	Lop Buri	14°51ʹ21ʺN 100°59ʹ24ʺE	H24	H14
PCK 2, 5 PCK 7,	Prachuap Khiri Khan	12°23ʹ42ʺN 99°55ʹ0ʺE	H22 H22	H13 H16
CTI 1, 2, 16	Chanthaburi	12°36′3″N 102°16′14″E	H14	H14
CMI 3	Chiang Mai	18°47′43″N 98°59′55″E	H22	H18
NWT 7 NWT 9	Narathiwat	6°25′N 101°49′E	H22 H25	H16 H19
NKI 1–3, 6, 7 NKI 10	Nong Khai	17°52′5″N 102°44′40″E	H26 H26	H19 H20
PNA 2, 4	Phang Nga	8°27′52″N 98°31′54″E	H22	H14
RNG 1–5, 7	Ranong	9°57′43″N 98°38′20″E	H24	H14
KKN 6	Khon Kaen	16°26′N 102°50′E	H25	H19

GenBank accession numbers are MG516589‐MG516592 for H22‐H25, respectively. Sample CMI 4 had H23 (Table S4), but we did not obtain the corresponding *CYTB* haplotype from this sample.

As pseudogenes are genes that have lost function, selection pressures, especially purifying, will be relaxed and the pseudogenes may accumulate indels resulting in frameshifts and possible premature stop codons. Also, substitution rates will become equal among codon positions; that is, the ratio of nonsynonymous to synonymous substitution rates (dN/dS = ω) will approach 1 (Bensasson, Zhang, Hartl, & Hewitt, 2001; Wertheim, Murrell, Smith, Kosakovsky Pond, & Scheffler, 2015). We used amino acid translated sequences to guide alignment between the *CYTB* of the six reference mtDNA samples and the untranslatable haplotypes, that is, candidate pseudogene haplotypes. Manual corrections to nucleotide alignments were made by eye. We inspected the alignment for indels that would cause frameshifts and premature stop codons.

We then tested for elevated ω and relaxed selection in the hypothesized pseudogene lineage. PAML v4.9d (Yang, 2007) via the PAMLX v1.3.1 GUI interface (Xu & Yang, 2013) was used to run codeml. The *CYTB* alignment file was the same as for the phylogenetic analysis above (Table S3). Gapped sites (i.e., ambiguous codons) are ignored in the analyses. A likelihood ratio test of branch models was used to determine whether there was elevated ω on the hypothesized pseudogene lineage. The null model (M0) of one ω ratio for all branches was compared to an alternative model of two ω ratios: one for the hypothesized pseudogene (foreground branches) and one for the remaining branches (background branches). Likelihood ratio tests 1 and 2 described in Zhang, Nielsen, and Yang (2005) were used to determine whether the elevated ω was due to relaxed selection as opposed to positive selection (e.g., see Pavlova et al., 2017). In both tests, branch‐site Model A is the alternative hypothesis. Branch‐site Model A model assumes four site classes: sites under purifying selection (0 < ω_0_ < 1); sites under neutrality (ω_1_ = 1); sites under positive selection ω_2_ > 1 in the foreground lineage but under purifying selection (0 < ω_0_ < 1) in background lineages; and sites under positive selection ω_2_ > 1 in the foreground lineage but under neutrality (ω_1_ = 1) in background lineages. Test 1 uses as a null the sites model M1a, which assumes two site classes for all branches: 0 < ω_0_ < 1 and ω_1_ = 1. Rejection of the null in Test 1 has good power to detect relaxed selection, but has a high false‐positive rate for positive selection (Zhang et al., 2005). Test 2 compares branch‐site Model A to a null where branch‐site Model A has ω_2_ = 1 in foreground branches. Test 2 has an appropriate type I error when testing for positive selection (Zhang et al., 2005). Thus, a significant Test 1 along with failure to reject in Test 2 would provide support for relaxed selection.

## 3 RESULTS

### 3.1 Phylogenetic analyses

**Figure 1:**
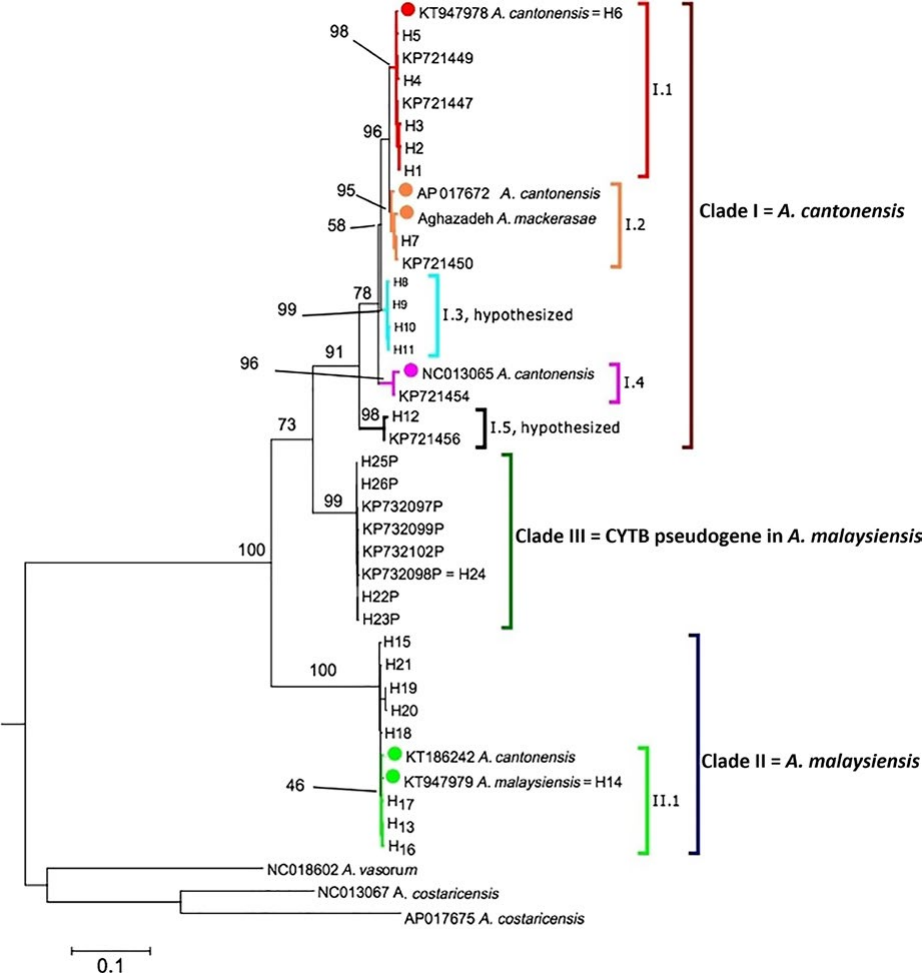
Maximum‐likelihood phylogenetic tree of the complete mtDNA *CYTB* dataset (Tables S1 and S3). The six reference mtDNA samples are indicated by circles. Clade I is anchored by three reference mtDNA samples originally identified as *Angiostrongylus cantonensis* (NC_013065, AP017672, and KT947978) and the sample identified as *A. mackerrasae*. Clade I is referred to as *A. cantonensis* (see main text for justification). Clade II is anchored by KT947979 (originally identified as *A. malaysiensis*; Yong et al., 2016) and KT186242 (originally identified as *A. cantonensis*, Yong, Song, et al., 2015). Clade II is referred to as *A. malaysiensis* (see main text for justification). Clade III represents a *CYTB* pseudogene lineage within specimens that fall in Clade II. Outgroups include *A. vasorum* (NC_018602), and *A. costaricensis* (NC_013067, and AP017675). Subclade designations (discussed in main text) that link the *CYTB* clades to *CO*1 clades are denoted by different colors. Numbers at nodes indicated bootstrap support (only nodes with >50% support or that are of particular interest are shown)

**Figure 2:**
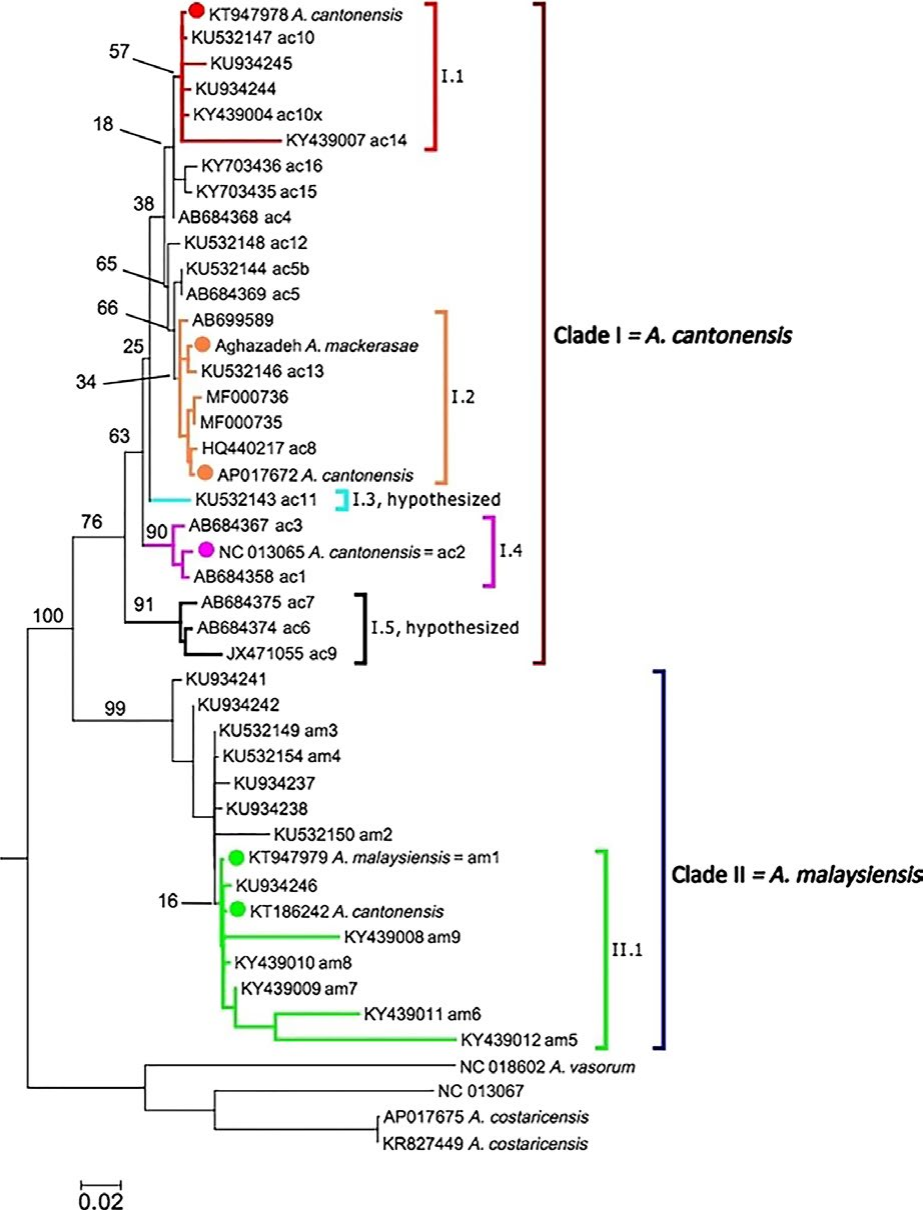
Maximum‐likelihood phylogenetic tree of the complete mtDNA *CO*1 dataset (Tables S2 and S4). The six reference mtDNA samples are indicated by circles. Clade I is anchored by three reference mtDNA samples originally identified as *Angiostrongylus cantonensis* (NC_013065, AP017672, and KT947978) and the sample identified as *A. mackerrasae*. Clade I is referred to as *A. cantonensis* (see main text for justification). Clade II is anchored by KT947979 (originally identified as *A. malaysiensis*; Yong et al., 2016) and KT186242 (originally identified as *A. cantonensis*, Yong, Song, et al., 2015). Clade II is referred to as *A. malaysiensis* (see main text for justification). Outgroups include *A. vasorum* (NC_018602 and NC_013067) and *A. costaricensis* (AP017675 and KR827449). Subclade designations (discussed in main text) that link the *CO*1 clades to *CYTB* clades are denoted by different colors. Numbers at nodes indicated bootstrap support (only nodes with >50% support or that are of particular interest are shown)

*CO*1 and *CYTB* phylogenetic trees based on the six mtDNA genome reference samples were identical in topology (data not shown) and matched their relationships observed in the results based on the complete *CO*1 and *CYTB* datasets (Figures 1 and 2). There is a significant and very high correlation in *CO*1 and *CYTB* pairwise *p*‐distances of the six reference samples (*r *=* *.99; *p *<* *.001; Table 2). Thus, as would be expected if there was no recombination, the two mtDNA genes show the same phylogenetic signal and are appropriate to use as anchors for the *CYTB* and *CO*1 haplotypes observed in previous studies.

**Table 2:** Pairwise *p*‐distance between the six mtDNA reference samples. *CO*1 is above the diagonal and *CYTB* is below

	AP017672	Aghazadeh	KT947978	NC013065	KT947979	KT186242
AP017672		0.009	0.018	0.041	0.090	0.090
Aghazadeh	0.006		0.019	0.041	0.090	0.090
KT947978	0.014	0.014		0.038	0.091	0.091
NC013065	0.036	0.038	0.036		0.086	0.086
KT947979	0.110	0.108	0.109	0.117		0.001
KT186242	0.112	0.110	0.111	0.119	0.002	

At the broadest level, two divergent clades are observed in the both the *CO*1 and *CYTB* datasets (Figures 1 and 2). Clade I is anchored by three reference mtDNA samples originally identified as *A. cantonensis* (NC_013065, AP017672, and KT947978) and the sample identified as *A. mackerrasae*, which is very closely related to AP017672. Clade II is anchored by KT947979 (originally identified as *A. malaysiensis*; Yong et al., 2016) and KT186242 (originally identified as *A. cantonensis*, Yong, Song, et al., 2015). A third clade, Clade III, was also identified in the *CYTB* dataset. Evidence is provided below that shows Clade III represents a *CYTB* pseudogene lineage within specimens that fall in Clade II.

Comparing the *CO*1 and *CYTB* phylogenetic trees, the reference samples enabled the linking of four subclades and relative phylogenetic positions provided hypothesized links between two additional subclades. Within Clade I, Subclade I.1 is anchored by the reference KT947978 (=*CYTB* Haplotype 6 of Dusitsittipon et al., 2017; red clade in Figures 1 and 2). Subclade I.2 was anchored by both AP017672 and the mtDNA genome sample of *A. mackerrasae* (orange clade in Figures 1 and 2). The reference NC_013065 linked Subclade I.4 (pink clade in Figures 1 and 2). Subclades I.3 and I.5 (aqua and black clades, respectively, in Figures 1 and 2) are the hypothesized clades based on relative phylogenetic positions; however, additional reference samples (i.e., samples from which both *CO*1 and *CYTB* have been obtained) are needed to verify these links. Within Clade II, the Subclade II.1 (light green clade in Figures 1 and 2) is anchored by the reference samples KT186242 and KT947979 (=*CYTB* Haplotype 14 of Dusitsittipon et al., 2017).

Generally, the subclade designations have strong bootstrap support (>90%) in the *CYTB* phylogeny (Figure 1). In contrast, bootstrap support for subclades is often low in the *CO*1 phylogeny (Figure 2) because many haplotypes are short (e.g., <500 nucleotides). As a result of our conservative approach, several *CO*1 haplotypes that were sister to Subclades I.1 or I.2 were not included in these subclades (Figure 2). Likewise, no additional linking was made in Clade II due to a lack of additional reference sequences and the low phylogenetic resolution among the *CO*1 or *CYTB* haplotypes in this clade. We also note that in Subclade II.1, a few haplotypes (KY439008, KY439011, KY439012) are short (~300 nucleotides), but show large divergence (i.e., long branches; Figure 2); hence, their inclusion in this clade should be considered tentative.

### 3.2 Pseudogene analyses

From prior studies (Dusitsittipon et al., 2015, 2017), the primers *Cytb*‐F and *Cytb*‐R produced sequences with uninterrupted amino acid translations (i.e., no premature stop codons) for specimens that only fall out in Clade I (Figure 1). In the current study, these same primers yielded five haplotypes (H22–H26) with interrupted amino acid *CYTB* translations from 41 specimens collected from various locations across Thailand (Table S1). Upon using primers *Cytb*‐F3 and *Cytb*‐R2, we obtained *CYTB* sequences with uninterrupted amino acid translations from 30 of these 41 specimens (Table 1). Hence, both translatable and untranslatable haplotypes were obtained from the same individuals. The translatable haplotypes (from primers *Cytb*‐F3 and *Cytb*‐R2) from all 30 of these individuals fall in Clade II (Table 1, Figure 1).

We also highlight that the *Cytb*‐F and *Cytb*‐R primers were used in a study by Yong, Eamsobhana, Song, et al. (2015) for samples they identified as *A. cantonensis* and *A. malaysiensis*. However, upon downloading from GenBank, it was observed that all of Yong, Eamsobhana, Song, et al. (2015) sequences labeled as *A. malaysiensis* were marked as “unverified” on GenBank (Table S1). Indeed, straight translation (i.e., no gaps in the alignment are inserted) of these sequences revealed premature stop codons. One haplotype we observed, H24, was the same as their AMM_1TAK sample (GenBank # KP732098) (Figure 1, Table S1). Aligning these “unverified” sequences with our own samples showed eight different untranslatable haplotypes. After alignment of these eight to the reference mtDNA samples, the untranslatable haplotypes had four common regions with deletions of six, one, one, and two nucleotides in length. It is after the first two deletion regions that there is a frameshift that leads to several premature stop codons in translation of the ungapped haplotypes. These eight haplotypes are largely distinguished from one another by unique single indels or a few single nucleotide polymorphisms (Table S3). The phylogenetic analyses showed that all eight of the untranslatable haplotypes fall into a single clade, Clade III, which is sister to Clade I (Figure 1). Note that because the maximum‐likelihood analysis treats gaps as missing data, some unique untranslatable haplotypes appear identical in the phylogenetic tree as their only differences are indels (Figure 1).

The branch test with two ω ratios (ω_background_ = 0.015, ω_foreground_ = 0.534, lnL = −3,033) was significantly favored over the M0 model of a single ratio (ω = 0.019, lnL = −3,037) for all branches (χ^2^ = 8, *df* = 1, *p *=* *.005). Hence, the hypothesized pseudogene branches show elevated ω. There was no difference in the branch‐site Model A (lnL = −3,025) and Model A with ω_2_ = 1 (lnL = −3,025), that is, ω_2_ was estimated to be 1 in Model A (Test 2, χ^2^ = 0, *df* = 1, *p *=* *1). However, Test 1 was significant were Model A was favored over M1a (lnL = −3,031; χ^2^ = 12, *df* = 2, *p *=* *.002). The results of Test 1 and Test 2 are consistent with relaxed selection on the hypothesized pseudogene lineage.

## 4 DISCUSSION

### 4.1 Incongruence between taxonomic identifications and mtDNA lineages

In Dusitsittipon et al. (2017), we observed two divergent *CYTB* clades (>11% *p*‐distance) among wild‐caught larval samples originally identified as *A. cantonensis* (based on morphology of adult worms from experimentally infected rats). At the time of our study, we had used two mtDNA genome samples as references in our phylogenetic analyses because they were identified in their respective publications as *A. cantonensis*. These were NC_013065 (Lv et al., 2012) and KT186242 (Yong, Eamsobhana, Lim, et al., 2015; Yong, Song, et al., 2015), both of which originated from laboratory‐maintained isolates in China or Thailand, respectively. Clade I contained NC_013065 and Clade II had KT186242. Moreover, we observed these two mtDNA clades were completely congruent with two genetically distant clusters that were based on 12 nuclear microsatellite loci (Dusitsittipon et al., 2017). As the mtDNA and microsatellites came from the same individuals, the complete non‐random association between mtDNA clades and microsatellite clusters provided conclusive evidence that these two clades represented distinct species (Dusitsittipon et al., 2017). In the current study after adding additional *CYTB* haplotypes along with four more mtDNA reference samples, these two *CYTB* clades remained clearly defined (Figure 1). Furthermore, these two clades, anchored by the six reference mtDNA samples, are clearly observed in the *CO*1 phylogeny (Figure 2).

While the current molecular data show clear evidence of two species as reflected by the two clades (Figures 1 and 2), there are evident incongruences between taxonomic identifications of samples and mtDNA clades when comparing the *CYTB* and *CO*1 phylogenies. With the exception of the *A. mackerrasae* reference (discussed below), all haplotypes (*CYTB* or *CO*1) that fall in Clade I originated from samples originally identified as *A. cantonensis* (Tables S1 and S2). In contrast, samples that fall in Clade II were identified as either *A. cantonensis* or *A. malaysiensis*.

In the *CO*1 phylogeny, several haplotypes from wild‐collected larval samples that were identified as *A. cantonensis* in Vitta et al. (2016) fall into Clade II (Table S2, Figure 2). Vitta et al. (2016) also used KT186242 as a *CO*1 reference in their phylogeny. Haplotypes from Rodpai et al. (2016), also from wild‐caught larval samples, fell into Clade II. Rodpai et al. (2016) referred to their samples as *A. malaysiensis* in conjunction with the finding of two diverged *CO*1 clades that corresponded to two 18S ribosomal DNA (rDNA) clades that had prior species‐designated reference sequences (see below). Eamsobhana, Yong, et al. (2017) identified adult, wild‐caught samples as *A. malaysiensis*, and their samples also fall in Clade II (Table S2; Figure 2).

Notably, we highlight that the reference sequence KT186242 (Yong, Song, et al., 2015) was based on the whole‐genome sequence of a Thai laboratory isolate identified as *A. cantonensis* in Yong, Eamsobhana, Lim, et al. (2015). The same group in the subsequent year (Yong et al., 2016) published the mtDNA genome of a wild‐caught adult sample from Malaysia that they identified as *A. malaysiensis* (KT947979). Yong et al. (2016) and Eamsobhana, Yong, et al. (2017) do not compare KT947979 to KT186242. We note that these two mtDNA genomes, which carry different species names in GenBank, fall in Clade II (Figures 1 and 2) and are extremely similar at the *CO*1 and *CYTB* (<0.2% *p*‐distance, Table 2).

Selective inclusion/exclusion of haplotypes among studies along with possible misidentifications due to similar morphologies of *A. malaysiensis* and *A. cantonensis* (discussed below) have likely led to these incongruences between taxonomy of samples and phylogenetic relationships of their respective haplotypes. Based on the fact that all samples for *CYTB* and *CO*1 in Clade I have been identified as *A. cantonensis* and that Eamsobhana, Yong, et al. (2017) identified wild‐caught adult worms a priori as *A. malaysiensis*, we henceforth refer to Clade I as *A. cantonensis* and Clade II as *A. malaysiensis*.

Further support for assigning these species names to these clades comes from Rodpai et al. (2016) in which they obtained both *CO*1 and 18S rDNA sequences from the same individuals. Rodpai et al. (2016) found complete congruence between two *CO*1 clades and two 18S clades where the two 18S clades had prior reference sequences designating them as *A. cantonensis* (GenBank AY295804; Carreno & Nadler, 2003) or *A. malaysiensis* (GenBank EF514914; Fontanilla & Wade, 2008). To facilitate future studies, we provided Tables S5 and S6, which provide sample information linking 18S haplotypes and *CO*1 haplotypes as well as the alignment of the unique 18S haplotypes of *A. malaysiensis* and *A. cantonensis* in fasta format. We also note that the nuclear 18S clades of Rodpai et al. (2016) are completely congruent with the two main diverged microsatellite clusters observed in Dusitsittipon et al. (2017), thereby reinforcing the species delimitation of clades I and II. Of special note, because KT186242 (Yong, Song, et al., 2015) would now be classified as being from *A. malaysiensis*, then the genome sequence in Yong, Eamsobhana, Lim, et al. (2015) also needs to be reclassified as coming from *A. malaysiensis* and not *A. cantonensis*.

### 4.2 *CYTB* pseudogene

We found strong evidence for a *CYTB* pseudogene within individuals that are in the *A. malaysiensis* clade. First, from 30 individuals (Table 1), we obtained both an untranslatable haplotype that resides in Clade III and a translatable haplotype that falls in Clade II (Figure 1). Second, the untranslatable haplotypes show the classic signs of a pseudogenized gene in having indels, which caused frameshifts and thus, downstream premature stop codons. The four shared deletion regions (Table S3) in all eight haplotypes of Clade III suggest the deletions happened in the ancestral haplotype of this clade. Third, molecular evolution analyses supported relaxed selection with elevated ω on the pseudogene lineage.

Incorrect phylogenetic inferences can be drawn if the pseudogene is treated as an ortholog to the real mtDNA genes (Ballard & Whitlock, 2004). Yong, Eamsobhana, Song, et al. (2015) used the *Cytb*‐F and *Cytb*‐R primers on samples identified as *A. malaysiensis* and recovered untranslatable haplotypes. As shown in our analyses, these haplotypes of Yong, Eamsobhana, Song, et al. (2015) fall into Clade III, that is, the pseudogene clade. Fortunately, no phylogenetic inferences were grossly misrepresented as *A. malaysiensis* is still sister to *A. cantonensis* using the orthologous *CO*1 or *CYTB* genes (Figures 1 and 2). Nonetheless, appropriate genetic distance comparisons would need to be made between clades I and II and not clades I and III (e.g., Rodpai et al., 2016; Yong, Eamsobhana, Song, et al., 2015).

### 4.3 Need to verify cryptic lineages and *A. mackerrasae* as species

Another interesting pattern obtained by collectively analyzing the *CO*1 and *CYTB* data is that the sample mtDNA genome from *A. mackerrasae* (Aghazadeh et al., 2015) is nested within samples that have been identified as *A. cantonensis* (Subclade I.2, Figures 1 and 2). In particular, the *A. mackerrasae* sample is very closely related to and less than 1% divergent (*p*‐distance) at the *CO*1 and *CYTB* (Table 2) from AP017672 (Figures 1 and 2), an unpublished GenBank entry that was part of the 50 Helminth Genomes Project (BioProject: PPRJEB493). AP017672 was identified as *A. cantonensis* from Taiwan with no other information given.

Recently Song, Yong, and Eamsobhana (2017) compared the whole mtDNA of *A. mackerrasae* (Aghazadeh, 2015) to KT947978 (Thailand isolate of *A. cantonensis*, Yong et al., 2016) and to NC_013065 (China isolate of *A. cantonensis*, Lv et al., 2012). They found across all protein coding genes the following pairwise *p*‐distances: *A. mackerrasae*‐KT947978 = 1.73%, *A. mackerrasae*‐NC_013065 = 3.7%, KT947978‐NC_013065 = 3.52%. These overall distances are reflected in the average of the *CO*1 and *CYTB p*‐distances given in Table 2. Because of the genetic similarity, they concluded “…that *A. mackerrasae* may be conspecific with *A. cantonensis* (Song et al., 2017). It remains to be resolved whether *A. mackerrasae* is conspecific with *A. cantonensis* or undergoing incipient speciation.” We agree with their argument and in general suggest concordance in multiple markers be used in species delimitation especially when mtDNA divergence is low (Janzen et al., 2017).

Our prior study showed concordant patterns between nuclear microsatellite markers and *CYTB* lineages (Dusitsittipon et al., 2017). Affiliations between the subclades designated herein, and these microsatellite clusters are given in Table 3. The non‐random associations between mtDNA haplotypes and nuclear clusters, along with *p*‐distances upwards of 6% between some *CYTB* haplotypes (e.g., between Subclades I.1 and I.5) within Clade I, are strong suggestive evidence of cryptic species within what is regarded as the single species *A. cantonensis* (Dusitsittipon et al., 2017). For example, from the Nan province of Thailand, Dusitsittipon et al. (2017) found two *CYTB* haplotypes (H7 and H9; Subclades I.2 and I.3, respectively) each of which corresponded to distinct microsatellite clusters (clusters 6 and 5, respectively). Thus, in sympatry, strong mtDNA–nuclear marker associations remain intact. As another example, *CYTB* haplotypes 13, 14, 16, 17 (Subclade II.1; Figure 1) are found only in samples that belong to microsatellite Cluster 8, but yet this cluster is dispersed across seven provinces in Thailand (Dusitsittipon et al., 2017). The maintenance of mtDNA–nuclear marker associations within sympatry and across geography indicates reproductive isolation. Hence, we hypothesize that *A. cantonensis* may in fact be a recently diverged species complex wherein the five subclades in Clade I may reflect different species.

**Table 3:** Subclade affiliations to microsatellite clusters from Dusitsittipon et al. (2017), country locations, and host species. Affiliations were made from sample information (Tables S1 and S2) from either the *CO*1 or *CYTB* haplotypes that fall within the respective clade. (L = laboratory‐maintained, W = wild‐collected, U = unknown origin. If no superscript is given, then it was wild‐collected.)

Subclade	I.1	I.2	I.3, hypothesized	I.4	I.5, hypothesized	II.1
Microsatellite cluster	1–3	6	5	No data	4	8
Countries	Thailand^L,W^	Taiwan^U^ Thailand^L,W^ Australia Japan United States, mainland Brazil	Thailand	China^L,W^ United States, Hawaii^L^ Japan Taiwan Myanmar	Thailand China^L,W^ Japan Brazil	Thailand^L,W^ Malaysia Taiwan
Mollusc hosts	*Achatina fulica* *Cryptozona siamensis*	*Achatina fulica*	*Achatina fulica*	*Achatina fulica* *Limax marginatus*	*Achatina fulica*	*Achatina fulica* *Cryptozona siamensis*
Mammal hosts	*Rattus rattus* *Rattus exulans* *Bandicota indica*	*Rattus norvegicus* *Rattus fuscipes* *Rattus rattus* *Bandicota indica* *Diplothrix legata* *Dasypus novemcinctu* *Didelphis virginiana*	*Rattus rattus*	*Rattus norvegicus* *Rattus rattus*	*Rattus norvegicus* *Rattus rattus*	*Rattus rattus diardii* *Rattus rattus* *Rattus norvegicus* *Bandicota savilei* *Rattus losea*

Due to low phylogenetic resolution in the *CYTB* of Clade II and due to a lack of additional reference sequences (i.e., samples from which both *CO*1 and *CYTB* have been obtained), it is not possible to link to the microsatellite clusters 7 and 9 of Dusitsittipon et al. (2017).

Returning to discussion of the *A. mackerrasae* sample, we draw particular attention to *CYTB* Haplotype 7, which was reported within Nakhon Si Thammarat and Nan, Thailand (Dusitsittipon et al., 2017). Haplotype 7 is very closely related to the *A. mackerrasae* sample (differs by 2 of 852 bp; Subclade I.2; Figure 1). *CYTB* Haplotype 7 was restricted to individuals that were in microsatellite Cluster 6 and no other haplotype was found in this cluster (*n *=* *15; Dusitsittipon et al., 2017). Again, as there was 100% concordance between nuclear and mtDNA data, this is suggestive evidence in support of *A. mackerrasae* as a distinct species. Nevertheless, additional multilocus data are needed to confirm whether there are independent evolutionary units (i.e., distinct species) within Clade I. For example, based on the collective analyses we conducted herein, we would predict that if *A. mackerrasae* is indeed a distinct species, then samples of morphologically identified *A. mackerrasae* from Australia would have a microsatellite profile that would cluster them with Cluster 6.

### 4.4 Evolutionary epidemiological inferences: gaps in linking the phylogenetic data to the life history and morphology

First, we highlight two caveats in linking the *CYTB* and *CO*1 subclades. (i) We are assuming that there is no effective recombination in the mtDNA. Mitochondrial recombination, which has been documented in a few animal species (Ladoukakis & Zouros, 2017), would preclude anchoring the two phylogenies. However, as noted in our results, congruent *CYTB* and *CO*1 topologies and pairwise *p*‐distances among the six mtDNA reference samples suggest recombination was not present in appreciable amounts. (ii) The many small *CO*1 haplotypes led to low bootstrap support for many of the subclades in the *CO*1 tree. Hence, the subclade links we made should be regarded as tentative hypotheses that need to be tested with additional data. Our goal was to begin making sense of the available sequence data in relation to the biology of rat lungworms; thus, the subclade designations were necessary to facilitate discussion and address future avenues of study. Below we relate our phylogenetic results to what is known for the life histories, morphology, and geographic distributions of *A. cantonensis*,* A. malaysiensis*, and *A. mackerrasae*. Admittedly, we probably indicate more gaps than links to the phylogenetic data, but by calling attention to these gaps, we hope to highlight critical areas for future study. We only briefly summarize some key traits below. For a more extensive discussion of the life histories of these three species, we refer readers to an excellent review by Spratt (2015).

*Angiostrongylus cantonensis*,* A. malaysiensis*, and *A. mackerrasae* have similar adult morphologies. These three species are largely distinguished by the adult male spicule lengths and separation or length of bursal rays (Bhaibulaya, 1968, 1979; Bhaibulaya & Cross, 1971; Chen, 1935). Bursal ray separation and length are very subtle and *A. malaysiensis* and *A. cantonensis* overlap in the range of spicule lengths with *A. cantonensis* being larger on average. *Angiostrongylus mackerrasae* has the smallest spicule length and does not overlap the other two (Bhaibulaya, 1979). Likewise, the larvae have very subtle morphological differences (Bhaibulaya, 1975). In part, these subtle differences or overlap in traits has likely lead to some of the taxonomic–mtDNA clade incongruences we noted above. Therefore, we call on future studies to be explicit in key traits such as spicule length.

Also, *A. cantonensis*,* A. malaysiensis*, and *A. mackerrasae* have been described as having similar life histories. They all infect the pulmonary arteries and right ventricle of various rodents. However, differences in developmental rates and pathological affects in primates have been reported (Bhaibulaya, 1979; Cross, 1979). In particular, Cross (1979) describes how experimental infections of monkeys with various geographic isolates of *A. cantonensis* (morphologically identified) resulted in greater pathological effects and death than compared to infections with *A. malaysiensis* or *A. mackerrasae* (monkeys survived infections with the latter species). In experimental infections of rats, Cross (1979) recovered a statistically lower percentage of adults of *A. malaysiensis* compared to *A. cantonensis*.

We are aware of only two studies that have looked at morphology and/or life history in relation to mtDNA isolates. Monte et al. (2014) examined the morphology of laboratory‐maintained worms that had the *CO*1 ac8 (Subclade I.2, Figure 2) and *CO*1 ac9 (Subclade I.5, Figure 2) haplotypes. Interestingly, *CO*1 ac8 is closely related to the *A. mackerrasae* sample. They reported significant differences in the spicule length (mm): 1.29 for worms with ac8 and 1.23 for those with ac9. However, both of these means fall in the range of *A. cantonensis* (1–1.46) and out of the range for *A. mackerrasae* (0.4–0.56) (Bhaibulaya, 1979). Future studies are needed to determine whether spicule length (and other traits) is affected by environmental background (e.g., intermediate or definitive host species). Monte et al. (2012) also report greater larval output with infections of *CO*1 ac9. In Lee et al. (2014), morphology and pathogenicity were examined between two *CO*1 mtDNA isolates, Pingtung (P) and Hualien (H), identified as *A. cantonensis* from Taiwan. There is difficulty in interpreting their sequence data (see Table S2). Nonetheless, they reported significantly lower recovery of adult worms from rats for the H isolate compared to the P isolate and smaller female body size in the H isolate. Lee et al. (2014) also reported greater pathogenicity and survival in rats infected with the P isolate. They indicated no differences in male body size, bursal number, bursal shape, or spicule morphology (though sizes not given).

In some other interesting life‐history studies, cross‐breeding experiments by Bhaibulaya (1974) and Cross and Bhaibulaya (1974) have shown that viable F1 hybrids could be generated between *A. cantonensis* × *A. mackerrasae* and *A. cantonensis* × *A. malaysiensis*, respectively. However, while the F1 females appeared to produce eggs (which we take to mean oocytes), the F1 males did not produce spermatozoa. In agreement, with the results of the latter cross, Dusitsittipon et al. (2017) found an F1 hybrid between Clade I (*A. cantonensis*) and Clade II (*A. malaysiensis*), using microsatellite markers. The mtDNA–nuclear concordance for clades I and II indicates there is little to no backcrossing of F1 hybrids, which is in agreement F1 sterility found by Cross and Bhaibulaya (1974).

Table 3 indicates both host (intermediate and definitive) and geographic locations from which clades have been recorded. Some caution is advised in interpreting these reports as sampling is not equal among studies. Also, the molecular‐based identifications are based on the mtDNA results in Figures 1 and 2. There are rDNA‐based surveys. However, we do not cover the rDNA‐based surveys here as the lack of variation in the rDNA precludes assignment to the subclade level. Despite these limitations, some results confirm past morphological‐based surveys and some interesting patterns have also emerged. For example, although originally described from Malaysia, there are morphological‐based reports of *A. malaysiensis* in Thailand (Spratt, 2015). In particular, a mixed infection of *A. cantonensis* and *A. malaysiensis* has been reported from rats in Bangkok, Thailand (Bhaibulaya & Techasoponmani, 1972). These morphological reports are now confirmed with the molecular‐based studies of Rodpai et al. (2016) and Dusitsittipon et al. (2017), wherein in the latter study, sympatric populations of *A. cantonensis* and *A. malaysiensis* were found in Thailand. In addition to the locations reported for Subclade II.1 (Thailand, Malaysia, Taiwan), other countries where the mtDNA data indicate the occurrence of *A. malaysiensis* include Myanmar and Laos (Tables S1 and S2). Thus, there are no mtDNA‐based identifications of *A. malaysiensis* outside South‐East Asia.

In contrast, mtDNA‐identified samples of *A. cantonensis*, Clade I, have not only been found throughout South‐East Asia, but also in Japan, Brazil, and the continental United States. Subclades I.1 and I.3 have only been reported from Thailand. Subclade I.4 is largely restricted to South‐East Asia and Japan. The Hawai'i report (GenBank KP721454 in Figure 1) in this subclade is from a Thailand laboratory‐maintained isolate by way of a Japan laboratory‐maintained isolate (Table S1). The other Hawai'i reports, *CO*1 haplotypes ac5b and ac5 (sister to Subclade I.2; Figure 2), are also based on laboratory‐maintained isolates in Japan or Thailand (Table S2). As the Hawai'i isolates are not in the same subclades, it appears that there may be more than one type of Hawai'i laboratory isolate. The most globally distributed subclades are I.2 and I.5. Subclade I.2 is interesting in that it contains the *A. mackerrasae* sample, which is the sole sample from Australia. Intriguingly, the larval samples from Dalton et al. (2017) (GenBank MF000735 and MF000736, Figure 2) that were reported from wildlife in the continental United States are closely related to *A. mackerrasae* (Figure 2). Thus, if Subclade I.2 represents *A. mackerrasae*, then caution is advised in interpreting past global reports of *A. cantonensis* as it is clear that Subclade I.2 has also been introduced to the Americas (Table 3).

## 5 SUMMARY

Our study represents an initial attempt to consolidate the available mtDNA data to link the findings of past studies. Assimilating the available mtDNA has highlighted three critical factors that investigators need to be aware of or resolve in future studies on the evolutionary epidemiology of *A. cantonensis*. First, there are incongruences between species identifications and mtDNA variants. We provided a resolution to this problem above and Tables [Link], [Link], [Link], [Link] to aid future studies. Second, we presented strong evidence of a *CYTB* pseudogene. The primers we designed should enable avoidance of this pseudogene. Third, current mtDNA–nuclear concordant patterns strongly hint at the presence of several cryptic species within Clade I and Clade II (Dusitsittipon et al., 2017). More multilocus‐based studies are needed to assess species delimitations and to test the validity of *A. mackerrasae*.

Eosinophilic meningitis has only been attributed to *A. cantonensis* (Barratt et al., 2016), and Spratt (2015) indicates there has not been unequivocal evidence to show that *A. mackerrasae* or *A. malaysiensis* are zoonotics. However, as discussed above, morphological identifications of these species are difficult due to their similarities. Thus, experiments are needed to determine trait distributions within clades (or subclades) and where possible, definitively link morphological traits to the molecular‐based phylogenetic lineages. In addition, current molecular diagnostic tools rely on rDNA (Qvarnstrom et al., 2010), which does not have as much variation as mtDNA. The geographic affiliations of subclades given in Table 3 suggest global distributions could be limited to just some subclades. Finer genetic marker resolution is needed because existing studies suggest variation in life history or pathogenicity among lineages (Lee et al., 2014; Monte et al., 2012). More studies are necessary to determine whether mtDNA subclades (possibly representing a species complex) are associated with life‐history, infectivity, or pathogenicity traits. Such studies will provide a clearer picture of the evolutionary epidemiology of *A. cantonensis* and closely related species as well as enable molecular diagnostics when examining outbreaks or varying pathologies.

## CONFLICT OF INTEREST

None declared.
