# Supplementary material for: Hurdles in the evolutionary epidemiology of Angiostrongylus cantonensis: Pseudogenes, incongruence between taxonomy and DNA sequence variants, and cryptic lineages
Source: Evol Appl. 2018 Mar 25;11(8):1257–69. doi: 10.1111/eva.12621 (PMC6099809; doi:10.1111/eva.12621)
Supplement: Supplementary file 3 [file EVA-11-1257-s003.docx]

>KP732097P

-GTTGAAAT-ATGGTAGAATATTGGGAATAATTTTAGTTTTTCAGTTGTTAACTGGTTTATTTTTATTTTTTTATTATGTTGCAGATGGTTTAGTGGCTTTTAGTTCGGTTCAGTATATTATGTCTGATGTTAATTTAGGTTGGTTATTTCGGATTTTTCATTTTAATGG------TTTGTTTTTTATTTTTT-GTATTTACATATTTTTAAGGGTTTGTTTATAGTTAGTTATCGATTGAAAAAAGTTTGGGGTACAGGTTTGTTAATTTTTTTATTGATTATGATAGAGGCTTTTATGGGTTATGTTTTAGTTTGGGCCCAAATAAGATTTTGAGCTGCGGTTGTGATTACTAGGTTGTTGAGTGTTGTTCCTGTTTGAGGTCAATTAATTGTAATGTGAATTTGAAGAGGTTTTGGGGTGACGAGATCAACTTTAAAATTTTTTTTT---GTTTTGCATTTTTTGTTACCTTGGTTAATGACGGTTTTGGTTATAGTGCATATGATTTTTTTACATAGGACGGGTAGGACTTCTAGTTTATATTGTCATAGTGATTATGATAAAATTAGTTTTGGTCCTTATTATTGAAATAAGGATTTTTATAACTTGTTAATTTTTTTTATTTTTTTTTTGCTAGTTTTGTTAAAGCCTTTTTACTTGGGCGATCCTGAAATGTTTATTGAGGCTGATCCTATGATGAGACCGGTACATATTGTTCCAGAGTGATATTTTT-ATTTGCCTATGCTATTTTACGGGCTATTCCTAATAAGGTTGTGGGTGTTTTGGCTTTATTGATGAGAATTTTATCATTT--TCTTTTTTTATTGGT-AGTAATTATACATCTTGTTTAG--

>KP732099P

-GTTGAAATTATGGTAGAATATTGGGAATAATTTTAGTTTTTCAGTTGTTAACTGGTTTATTTTTATTTTTTTATTATGTTGCAGATGGTTTAGTGGCTTTTAGTTCGGTTCAGTATATTATGTCTGATGTTAATTTAGGTTGGTTATTTCGGATTTTTCATTTTAATGG------TTTGTTTTTTATTTTTT-GTATTTACATATTTTTAAGGGTTTGTTTATAGTTAGTTATCGATTGAAAAAAGTTTGGGGTACAGGTTTGTTAATTTTTTTATTGATTATGATAGAGGCTTTTATGGGTTATGTTTTAGTTTGGGCCCAAATAAGATTTTGAGCTGCGGTTGTGATTACTAGGTTGTTGAGTGTTGTTCCTGTTTGAGGTCAATTAATTGTAATGTGAATTTGAAGAGGTTTTGGGGTGACGAGATCAACTTTAAAATTTTTTTTT---GTTTTGCATTTTTTGTTACCTTGGTTAATGACGGTTTTGGTTATAGTGCATATGATTCTTTTACATAGGACGGGTAGGACTTCTAGTTTATATTGTCATAGTGATTATGATAAAATTAGTTTTGGTCCTTATTATTGAAATAAGGATTTTTATAACTTGTTAATTTTTTTTATTTTTTTTTTGCTAGTTTTGTTAAAGCCTTTTTACTTGGGCGATCCTGAAATGTTTATTGAGGCTGATCCTATGATGAGACCGGTACATATTGTTCCAGAGTGATATTTTT-ATTTGCCTATGCTATTTTACGGGCTATTCCTAATAAGGTTGTGGGTGTTTTGGCTTTATTGATGAGAATTTTATCATTT--TCTTTTTTTATTGGTTAGTAATTATACATCTTGTTTAG--

>KP732102P

-GTTGAAATTATGGTAGAATATTGGGAATAATTTTAGTTTTTCAGTTGTTAACTGGTTTATTTTTATTTTTTTATTATGTTGCAGATGGTTTAGTGGCTTTTAGTTCGGTTCAGTATATTATGTCTGATGTTAATTTAGGTTGGTTATTTCGGATTTTTCATTTTAATGG------TTTGTTTTTTATTTTTT-GTATTTACATATTTTTAAGGGTTTGTTTATAGTTAGTTATCGATTGAAAAAAGTTTGGGGTACAGGTTTGTTAATTTTTTTATTGATTATGATAGAGGCTTTTATGGGTTATGTTTTAGTTTGGGCCCAAATAAGATTTTGAGCTGCGGTTGTGATTACTAGGTTGTTGAGTGTTGTTCCTGTTTGAGGTCAATTAATTGTAATGTGAATTTGAAGAGGTTTTGGGGTGACGAGATCAACTTTAAAATTTTTTTTT---GTTTTGCATTTTTTGTTACCTTGGTTAATGACGGTTTTGGTTATAGTGCATATGATTTTTTTACATAGGACGGGTAGGACTTCTAGTTTATATTGTCATAGTGATTATGATAAAATTAGTTTTGGTCCTTATTATTGAAATAAGGATTTTTATAACTTGTTAATTTTTTTTATTTTTTTTTTGCTAGTTTTGTTAAAGCCTTTTTACTTGGGCGATCCTGAAATGTTTATTGAGGCTGATCCTATGATGAGACCGGTACATATTGTTCCAGAGTGATATTTTT-ATTTGCCTATGCTATTTTACGGGCTATTCCTAATAAGGTTGTGGGTGTTTTGGCTTTATTGATGAGAATTTTATCATTT--TCTTTTTTTATTG-TAAGTAATTATACATCTTGTTTAG--

>KP732098P

-GTTGAAATTATGGTAGAATATTGGGAATAATTTTAGTTTTTCAGTTGTTAACTGGTTTATTTTTATTTTTTTATTATGTTGCAGATGGTTTAGTGGCTTTTAGTTCGGTTCAGTATATTATGTCTGATGTTAATTTAGGTTGGTTATTTCGGATTTTTCATTTTAATGG------TTTGTTTTTTATTTTTT-GTATTTACATATTTTTAAGGGTTTGTTTATAGTTAGTTATCGATTGAAAAAAGTTTGGGGTACAGGTTTGTTAATTTTTTTATTGATTATGATAGAGGCTTTTATGGGTTATGTTTTAGTTTGGGCCCAAATAAGATTTTGAGCTGCGGTTGTGATTACTAGGTTGTTGAGTGTTGTTCCTGTTTGAGGTCAATTAATTGTAATGTGAATTTGAAGAGGTTTTGGGGTGACGAGATCAACTTTAAAATTTTTTTTT---GTTTTGCATTTTTTGTTACCTTGGTTAATGACGGTTTTGGTTATAGTGCATATGATTTTTTTACATAGGACGGGTAGGACTTCTAGTTTATATTGTCATAGTGATTATGATAAAATTAGTTTTGGTCCTTATTATTGAAATAAGGATTTTTATAACTTGTTAATTTTTTTTATTTTTTTTTTGCTAGTTTTGTTAAAGCCTTTTTACTTGGGCGATCCTGAAATGTTTATTGAGGCTGATCCTATGATGAGACCGGTACATATTGTTCCAGAGTGATATTTTT-ATTTGCCTATGCTATTTTACGGGCTATTCCTAATAAGGTTGTGGGTGTTTTGGCTTTATTGATGAGAATTTTATCATTT--TCTTTTTTTATTGGTTAGTAATTATACATCTTGTTTAG--

>H22P

-GTTGAAATTATGGTAGAATATTGGGAATAATTTTAGTTTTTCAGTTGTTAACTGGTTTATTTTTATTTTTTTATTATGTTGCAGATGGTTTAGTGGCTTTTAGTTCGGTTCAGTATATTATGTCTGATGTTAATTTAGGTTGGTTATTTCGGATTTTTCATTTTAATGG------TTTGTTTTTTATTTTTT-GTATTTACATATTTTTAAGGGTTTGTTTATAGTTAGTTATCGATTGAAAAAAGTTTGGGGTACAGGTTTGTTAATTTTTTTATTGATTATGATAGAGGCTTTTATGGGTTATGTTTTAGTTTGGGCCCAAATAAGATTTTGAGCTGCGGTTGTGATTACTAGGTTGTTGAGTGTTGTTCCTGTTTGAGGTCAATTAATTGTAATGTGAATTTGAAGAGGTTTTGGGGTGACGAGATCAACTTTAAAATTTTTTTTT---GTTTTGCATTTTTTGTTACCTTGGTTAATGACGGTTTTGGTTATAGTGCATATGATTTTTTTACATAGGACGGGTAGGACTTCTAGTTTATATTGTCATAGTGATTATGATAAAATTAGTTTTGGTCCTTATTATTGAAATAAGGATTTTTATAACTTGTTAATTTTTTTTATTTTTTTTTTGCTAGTTTTGTTAAAGCCTTTTTACTTGGGCGATCCTGAAATGTTTATTGAGGCTGATCCTATGATGAGACCGGTACATATTGTTCCAGAGTGATATTTTT-ATTTGCCTATGCTATTTTACGGGCTATTCCTAATAAGGTTGTGGGTGTTTTGGCTTTATTGATGAGAATTTTATCATTT--TCTTTTTTTATTGGTTAGTAATTATACAT-TTGTTTAG--

>H23P

-GTTGAAATTATGGTAGAATATTGGGAATAATTTTAGTTTTTCAGTTGTTAACTGGTTTATTTTTATTTTTTTATTATGTTGCAGATGGTTTAGTGGCTTTTAGTTCGGTTCAGTATATTATGTCTGATGTTAATTTAGGTTGGTTATTTCGGATTTTTCATTTTAATGG------TTTGTTTTTTATTTTTT-GTATTTACATATTTTTAAGGGTTTGTTTATAGTTAGTTATCGATTGAAAAAAGTTTGGGGTACAGGTTTGTTAATTTTTTTATTGATTATGATAGAGGCTTTTATGGGTTATGTTTTAGTTTGGGCCCAAATAAGATTTTGAGCTGCGGTTGTGATTACTAGGTTGTTGAGTGTTGTTCCTGTTTGAGGTCAATTAATTGTAATGTGAAATTGAAGAGGTTTTGGGGTGACGAGATCAACTTTAAAATTTTTTTTT---GTTTTGCATTTTTTGTTACCTTGGTTAATGACGGTTTTGGTTATAGTGCATATGATTTTTTTACATAGGACGGGTAGGACTTCTAGTTTATATTGTCATAGTGATTATGATAAAATTAGTTTTGGTCCTTATTATTGAAATAAGGATTTTTATAACTTGTTAATTTTTTTTATTTTTTTTTTGCTAGTTTTGTTAAAGCCTTTTTACTTGGGCGATCCTGAAATGTTTATTGAGGCTGATCCTATGATGAGACCGGTACATATTGTTCCAGAGTGATATTTTT-ATTTGCCTATGCTATTTTACGGGCTATTCCTAATAAGGTTGTGGGTGTTTTGGCTTTATTGATGAGAATTTTATCATTT--TCTTTTTTTATTGGTTAGTAATTATACAT-TTGTTTAG--

>H25P

-GTTGAAATTATGGTAGAATATTGGGAATAATTTTAGTTTTTCAGTTGTTAACTGGTTTATTTTTATCTTTTTATTATGTTGCAGATGGTTTAGTGGCTTTTAGTTCGGTTCAGTATATTATGTCTGATGTTAATTTAGGTTGGTTATTTCGGATTTTTCATTTTAATGG------TTTGTTTTTTATTTTTT-GTATTTACATATTTTTAAGGGTTTGTTTATAGTTAGTTATCGATTGAAAAAAGTTTGGGGTACAGGTTTGTTAATTTTTTTATTGATTATGATAGAGGCTTTTATGGGTTATGTTTTAGTTTGGGCCCAAATAAGATTTTGAGCTGCGGTTGTGATTACTAGGTTGTTGAGTGTTGTTCCTGTTTGAGGTCAATTAATTGTAATGTGAATTTGAAGAGGTTTTGGGGTGACGAGATCAACTTTAAAATTTTTTTTT---GTTTTGCATTTTTTGTTACCTTGGTTAATGACGGTTTTGGTTATAGTGCATATGATTTTTTTACATAGGACGGGTAGGACTTCTAGTTTATATTGTCATAGTGATTATGATAAAATTAGTTTTGGTCCTTATTATTGAAATAAGGATTTTTATAACTTGTTAATTTTTTTTATTTTTTTTT-GCTAGTTTTGTTAAAGCCTTTTTACTTGGGCGATCCTGAAATGTTTATTGAGGCTGATCCTATGATGAGACCGGTACATATTGTTCCAGAGTGATATTTTT-ATTTGCCTATGCTATTTTACGGGCTATTCCTAATAAGGTTGTGGGTGTTTTGGCTTTATTGATGAGAATTTTATCATTT--TCTTTTTTTATTGGTTAGTAATTATACAT-TTGTTTAG--

>H26P

-GTTGAAATTATGGTAGAATATTGGGAATAATTTTAGTTTTTCAGTTGTTAACTGGTTTATTTTTATCTTTTTATTATGTTGCAGATGGTTTAGTGGCTTTTAGTTCGGTTCAGTATATTATGTCTGATGTTAATTTAGGTTGGTTATTTCGGATTTTTCATTTTAATGG------TTTGTTTTTTATTTTTT-GTATTTACATATTTTTAAGGGTTTGTTTATAGTTAGTTATCGATTGAAAAAAGTTTGGGGTACAGGTTTGTTAATTTTTTTATTGATTATGATAGAGGCTTTTATGGGTTATGTTTTAGTTTGGGCCCAAATAAGATTTTGAGCTGCGGTTGTGATTACTAGGTTGTTGAGTGTTGTTCCTGTTTGAGGTCAATTAATTGTAATGTGAATTTGAAGAGGTTTTGGGGTGACGAGATCAACTTTAAAATTTTTTTTT--TGTTTTGCATTTTTTGTTACCTTGGTTAATGACGGTTTTGGTTATAGTGCATATGATTTTTTTACATAGGACGGGTAGGACTTCTAGTTTATATTGTCATAGTGATTATGATAAAATTAGTTTTGGTCCTTATTATTGAAATAAGGATTTTTATAACTTGTTAATTTTTTTTATTTTTTTTT-GCTAGTTTTGTTAAAGCCTTTTTACTTGGGCGATCCTGAAATGTTTATTGAGGCTGATCCTATGATGAGACCGGTACATATTGTTCCAGAGTGATATTTTT-ATTTGCCTATGCTATTTTACGGGCTATTCCTAATAAGGTTGTGGGTGTTTTGGCTTTATTGATGAGAATTTTATCATTT--TCTTTTTTTATTGGTTAGTAATTATACATCTTGTTTAG--

>NC013067 A. costaricensis

-ATTGGAATTATGGTAGTATGTTAGGGATAGTTTTGTTGTTTCAGATATTAACTGGTTTGTTTTTGTCTTTTTATTATGTAGCGGATGGTATGTTAGCTTTTAGATCAGTTCAATATATTATGTATGATGTTAATTTGGGTTGGTTATTTCGAATTTTTCATTTTAATGGAGCAAGATTATTTTTTATTTTTTTGTATCTGCATATTTTTAAGGGTTTATTAATAATAAGATATCGTTTGAAGAAGGTGTGGGGTACTGGTTTATTGATTTTTTTATTGATTATAATGGAGGCCTTTATAGGTTATGTATTGGTTTGGGCTCAGATGAGGTTCTGGGCTGCGGTGGTGATTACTAGTTTGTTAAGAGTTGTTCCTGTTTGGGGTCAGTTAATTGTGATATGAATTTGAAGTGGTTTTGGGGTTACAAGTTCTACTTTGAAATTTTTTTTT---GTTTTGCATTTTTTATTGCCGTGATTTTTGATGGTTTTAGTGATAATTCATATAATTTGTTTACATAGGACAGGAAGAACTTCTAGTCTTTATTGTCATGGGGATTATGATAAAATTAGTTTTGGTCCTTATTATTGAAATAAAGATTTTTATAATTTGATAGGTTTTATAATATTTTTTATTTTTGTGTTAGTTAGACCTTTTTTGTTGGGTGATCCGGAAATGTTTGTTGAGGCTGATCCTATAACTAGTCCGGTTCATATTGTACCTGAATGGTATTTTTTATTTGCTTATGCTATTTTACGAGCTATTCCTAATAAGGTTTTGGGTGTTTTAGCTTTGTTAATAAGGATTATATTTTTTTATTTTTTTTTGTTAGTGGATAATTATACATCATGTATAG--

>AP017675 A. costaricensis

-ATTGAAATTATGGGAGTATATTAGGGATAATTTTGTTATTTCAATTATTGACTGGTTTATTTTTATCTTTTTATTATGTAGCAGATGGTGTAATAGCTTTTGGTTCAGTTCAATATATTATGTTCGATGTTAATTTGGGTTGATTATTTCGAATTTTTCATTTTAATGGGGCTAGGCTTTTTTTTATTTTTATATATCTTCATATTTTTAAGGGTTTATTTATAATGAGTTATCGTTTAAAGTTAGTTTGAGGAACTGGTTTAGTGATTTTTTTATTAGTTATATTAGAAGCTTTTATGGGTTATGTGTTAGTTTGGGCTCAAATGAGGTTTTGGGCTGCCGTGGTGATTACTAGTTTGTTGAGAGTTATTCCTGTTTGAGGACAGTTGATTGTGATATGAATTTGAAGGGGTTTTGGGGTAACTAGTTCTACTTTGAAGTTTTTTTTT---GCTTTACATTTTTTGTTACCTTGATTTTTAGTAGTTTTGGTTATAGTTCATATAATTTTTTTGCATAGAACGGGGAGAACTTCTGGATTGTATTGTCACGGGGATTATGATAAAATTAGTTTTGGCCCTTATTATTGAAATAAAGATTTTTATAATATAGTAGGATTTTTGTTGTTTTTTATTTTTGTATTAGTTAGACCCTTTTTGTTAGGAGATCCTGAAATATTTGTTGAGGCTGATCCTATAACTAGTCCAGTACATATTGTGCCGGAATGGTATTTTTTGTTTGCTTATGCTATTTTACGTGCTATTCCTAATAAAGTTTTAGGTGTTTTGGCTTTATTAATAAGGGTTTTGTCTTTTTTTTTTTTTTTATTAGTGGGGAATTATACATCTTGTATGG--

>NC018602 A. vasorum

-GTTGGAATTATGGGAGGATGTTGGGGATGGTTTTGTTGTTCCAGGTATTGACTGGTTTGTTTTTATCTTTTTACTATGTGGCGGATGGATTGATAGCCTTTAGGTCGGTGCAGTATATTATGTCTGATGTTGGTTTGGGTTGGTTGTTTCGGATTTTTCATTTTAATGGGGCTAGTTTGTTTTTTGTTTTTTTATATATGCATATTTTTAAGGGTTTATTTTTTATAAGTTATCGTTTGAAGAAGGTTTGGGGTACTGGTTTGTTGATTTTTTTGTTGGTTATAATGGAGGCCTTTATAGGTTATGTGTTGGTTTGGGCTCAAATGAGATTTTGGGCTGCGGTGGTAATTACTAGTTTGTTAAGAGTGATTCCTATTTGGGGCCAGTTGATTGTGATGTGGATTTGAGGAGGTTTTGGGGTTACAAGGTCTACTTTAAAGTTTTTTTTT---GTTTTACATTTTTTGTTGCCTTGGTTTTTGATGGTGTTAGTAATAGTGCATATGGTTTTTTTACATAGAAGGGGGAGTACTTCTAGGTTGTATTGTCATGGAGATTATGATAAAATTGTTTTTGGTCCTTATTATTGGAATAAGGACTTTTATAATTTGATGGTGTTTGTAGTTTTTTTTGTGTTTGTTTTGTTAAAGCCTTTTGTTTTGGGGGATCCGGAAATATTTATTGAAGCTGATCCGATGACTAGTCCAGTCCATATTGTGCCGGAGTGGTATTTTTTGTTTGCGTATGCTATTTTACGAGCTATTCCTAATAAGGTTTTGGGTGTTTTGGCTTTGTTGATGAGTATCTTGATGTTTTATTTTTTTTTGATAGTGGGGAATTATACGTCTTGTTTGA--

>H1

-GTTGAAATTATGGTAGAATGTTGGGAATGATTTTAGTTTTTCAGTTGTTAACTGGATTATTTTTATCTTTTTATTATGTTGCGGATGGTTTAGTGGCTTTTAGTTCGGTTCAATATGTTATATTTGATGTTAATTTGGGTTGGTTATTTCGGATTTTTCATTTTAATGGGGCGAGTTTGTTTTTTATTTTTTTGTATTTGCATATTTTTAAGGGTTTGTTTATAGTTAGTTATCGGTTGAAAAAAGTTTGAGGTACGGGTTTATTGATTTTTTTGTTGATTATGATAGAGGCTTTTATGGGTTATGTTTTAGTTTGGGCTCAAATAAGATTTTGAGCTGCGGTTGTAATTACTAGGTTGTTGAGTGTTGTTCCTGTTTGAGGTCAGTTGATTGTAATGTGAATTTGAAGAGGTTTTGGAGTAACCAGGTCAACTTTAAAGTTTTTTTTT---GTTTTACATTTTTTGTTACCTTGGTTATTGTTGGTTTTGGTTATAGTGCATATGATTTTTTTGCATAGTACTGGTAGAACTTCTAGTTTGTATTGTCATGGTGATTATGATAAAATTAGTTTTGGTCCTTATTATTGGAATAAGGATTTTTATAATTTTTTAGTTTTTTTTGTTTTTTTTTTGTTAGTTTTGTTGAAGCCTTTTTATTTAGGAGATCCCGAAATGTTTGTTGAGGCAGATCCCATGGTGAGCCCAGTGCATATTGTTCCAGAATGGTATTTTTTGTTTGCTTATGCTATTCTGCGAGCTATTCCTAATAAGGTTATGGGTGTTGTAGCTTTGTTGATAAGAATTTTGTTATTTTATTTTTTTTTGTTGGTTAGTAATTATACGTCTTGTTTGG--

>H2

-GTTGAAATTATGGTAGAATGTTGGGAATGATTTTAGTTTTTCAGTTGTTAACTGGATTATTTTTATCTTTTTATTATGTTGCGGATGGTTTAGTGGCTTTTAGTTCGGTTCAATATGTTATATTTGATGTTAATTTGGGTTGGTTATTTCGGATTTTTCATTTTAATGGGGCGAGTTTGTTTTTTATTTTTTTGTATTTGCATATTTTTAAGGGTTTGTTTATAGTTAGTTATCGGTTGAAAAAAGTTTGAGGTACGGGTTTATTGATTTTTTTGTTGATTATGATAGAGGCTTTTATGGGTTATGTTTTAGTTTGGGCTCAAATAAGATTTTGAGCTGCGGTTGTAATTACTAGGTTGTTGAGTGTTGTTCCTGTTTGAGGTCAGTTGATTGTAATGTGAATTTGAAGAGGTTTTGGAGTAACCAGGTCAACTTTAAAGTTTTTTTTT---GTTTTACATTTTTTGTTACCTTGGTTATTGTTGGTTTTGGTTATAGTGCATATGATTTTTTTGCATAGTACTGGTAGAACTTCTAGTTTGTATTGTCATGGTGATTATGATAAAATTAGTTTTGGTCCTTATTATTGGAATAAGGATTTTTATAATTTTTTAGTTTTTTTTGTTTTTTTTTTGTTAGTTTTGTTGAAGCCTTTTTATTTAGGAGATCCCGAAATGTTTGTTGAGGCAGATCCCATGGTGAGCCCAGTGCATATTGTTCCAGAATGGTATTTTTTGTTTGCTTATGCTATTCTGCGAGCTATTCCTAATAAGGTTATGGGTGTTGTAGCTTCGTTGATAAGAATTTTGTTATTTTATTTTTTTTTGTTGGTTAGTAATTATACGTCTTGTTTGG--

>H3

-GTTGAAATTATGGTAGAATGTTGGGAATGATTTTAGTTTTTCAGTTGTTAACTGGATTATTTTTATCTTTTTATTATGTTGCGGATGGTTTAGTGGCTTTTAGTTCGGTTCAATATGTTATATTTGATGTTAATTTGGGTTGGTTATTTCGGATTTTTCATTTTAATGGGGCGAGTTTGTTTTTTATTTTTTTGTATTTGCATATTTTTAAGGGTTTGTTTATAGTTAGTTATCGGTTGAAAAAAGTTTGAGGTACGGGTTTATTGATTTTTTTGTTGATTATGATAGAGGCTTTTATGGGTTATGTTTTAGTTTGGGCTCAAATAAGATTTTGAGCTGCGGTTGTAATTACTAGGTTGTTGAGTGTTGTTCCTGTTTGAGGTCAGTTGATTGTAATGTGAATTTGAAGAGGTTTTGGAGTAACCAGGTCAACTTTAAAGTTTTTTTTT---GTTTTACATTTTTTGTTACCTTGGTTATTGTTGGTTTTGGTTATGGTGCATATGATTTTTTTGCATAGTACTGGTAGAACTTCTAGTTTGTATTGTCATGGTGATTATGATAAAATTAGTTTTGGTCCTTATTATTGGAATAAGGATTTTTATAATTTTTTAGTTTTTTTTGTTTTTTTTTTGTTAGTTTTGTTGAAGCCTTTTTATTTAGGAGATCCCGAAATGTTTGTTGAGGCAGATCCCATGGTGAGCCCAGTGCATATTGTTCCAGAATGGTATTTTTTGTTTGCTTATGCTATTCTGCGAGCTATTCCTAATAAGGTTATGGGTGTTGTAGCTTTGTTGATAAGAATTTTGTTATTTTATTTTTTTTTGTTGGTTAGTAATTATACGTCTTGTTTGG--

>H4

-GTTGAAATTATGGTAGAATGTTGGGGATAATTTTAGTTTTTCAGTTGTTAACTGGATTATTTTTATCTTTTTATTATGTTGCGGATGGTTTAGTGGCTTTTAGTTCGGTTCAATATGTTATATTTGATGTTAATTTGGGTTGGTTATTTCGGATTTTTCATTTTAATGGGGCGAGTTTGTTTTTTATTTTTTTGTATTTGCATATTTTTAAGGGTTTGTTTATAGTTAGTTATCGGTTGAAAAAAGTTTGAGGTACGGGTTTATTGATTTTTTTGTTGATTATGATAGAGGCTTTTATGGGTTATGTTTTAGTTTGGGCTCAAATAAGATTTTGAGCTGCGGTTGTAATTACTAGGTTGTTGAGTGTTGTTCCTTTTTGAGGTCAGTTGATTGTAATGTGAATTTGAAGAGGTTTTGGAGTAACCAGGTCAACTTTAAAGTTTTTTTTT---GTTTTACATTTTTTGTTACCTTGGCTATTGTTGGTTTTGGTTATAGTGCATATGATTTTTTTGCATAGTACTGGTAGAACTTCTAGTTTGTATTGTCATGGTGATTATGATAAAATTAGTTTTGGTCCTTATTATTGGAATAAGGATTTTTATAATTTTTTAGTTTTTTTTGTTTTTTTTTTGTTAGTTTTGTTGAAGCCTTTTTATTTAGGAGATCCCGAAATGTTTGTTGAGGCAGATCCCATGGTGAGCCCAGTGCATATTGTTCCAGAATGGTATTTTTTGTTTGCTTATGCTATTTTGCGAGCTATTCCTAATAAGGTTATGGGTGTTGTAGCTTTGTTGATAAGAATTTTGTTATTTTATTTTTTTTTGTTGGTTAGTAATTATACGTCTTGTTTGG--

>H5

-GTTGAAATTATGGTAGAATGTTGGGAATAATTTTAGTTTTTCAGTTGTTAACTGGATTATTTTTATCTTTTTATTATGTTGCGGATGGTTTAGTGGCTTTTAGTTCGGTTCAATATGTTATATTTGATGTTAATTTGGGTTGGTTATTTCGGATTTTTCATTTTAATGGGGCGAGTTTGTTTTTTATTTTTTTGTATTTGCATATTTTTAAGGGTTTGTTTATAGTTAGCTATCGGTTGAAAAAAGTTTGAGGTACGGGTTTATTGATTTTTTTGTTGATTATGATAGAGGCTTTTATGGGTTATGTTTTAGTTTGGGCTCAAATAAGATTTTGAGCTGCGGTTGTAATTACTAGGTTGTTGAGTGTTGTTCCTGTTTGAGGTCAGTTGATTGTAATGTGAATTTGAAGAGGTTTTGGAGTAACCAGGTCAACTTTAAAGTTTTTTTTT---GTTTTACATTTTTTGTTACCTTGGCTATTGTTGGTTTTGGTTATAGTGCATATGATTTTTTTGCATAGTACTGGTAGAACTTCTAGTTTGTATTGTCATGGTGATTATGATAAAATTAGTTTTGGTCCTTATTATTGGAATAAGGATTTTTATAATTTTTTAGTTTTTTTTGTTTTTTTTTTGTTAGTTTTGTTGAAGCCTTTTTATTTAGGAGATCCCGAAATGTTTGTTGAGGCAGATCCCATGGTGAGCCCAGTGCATATTGTTCCAGAATGGTATTTTTTGTTTGCTTATGCTATTTTGCGAGCTATTCCTAATAAGGTTATGGGTGTTGTAGCTTTGTTGATAAGAATTTTGTTATTTTATTTTTTTTTGTTGGTTAGTAATTATACGTCTTGTTTGG--

>KT947978 A. cantonensis

-GTTGAAATTATGGTAGAATGTTGGGAATAATTTTAGTTTTTCAGTTGTTAACTGGATTATTTTTATCTTTTTATTATGTTGCGGATGGTTTAGTGGCTTTTAGTTCGGTTCAATATGTTATATTTGATGTTAATTTGGGTTGGTTATTTCGGATTTTTCATTTTAATGGGGCGAGTTTGTTTTTTATTTTTTTGTATTTGCATATTTTTAAGGGTTTGTTTATAGTTAGTTATCGGTTGAAAAAAGTTTGAGGTACGGGTTTATTGATTTTTTTGTTGATTATGATAGAGGCTTTTATGGGTTATGTTTTAGTTTGGGCTCAAATAAGATTTTGAGCTGCGGTTGTAATTACTAGGTTGTTGAGTGTTGTTCCTGTTTGAGGTCAGTTGATTGTAATGTGAATTTGAAGAGGTTTTGGAGTAACCAGGTCAACTTTAAAGTTTTTTTTT---GTTTTACATTTTTTGTTACCTTGGCTATTGTTGGTTTTGGTTATAGTGCATATGATTTTTTTGCATAGTACTGGTAGAACTTCTAGTTTGTATTGTCATGGTGATTATGATAAAATTAGTTTTGGTCCTTATTATTGGAATAAGGATTTTTATAATTTTTTAGTTTTTTTTGTTTTTTTTTTGTTAGTTTTGTTGAAGCCTTTTTATTTAGGAGATCCCGAAATGTTTGTTGAGGCAGATCCCATGGTGAGCCCAGTGCATATTGTTCCAGAATGGTATTTTTTGTTTGCTTATGCTATTTTGCGAGCTATTCCTAATAAGGTTATGGGTGTTGTAGCTTTGTTGATAAGAATTTTGTTATTTTATTTTTTTTTGTTGGTTAGTAATTATACGTCTTGTTTGG--

>H7

-GTTGGAATTATGGTAGAATGTTGGGGATAATTTTAGTTTTTCAGTTGTTAACTGGATTATTTTTATCTTTTTATTATGTTGCGGATGGTTTAGTGGCTTTTAGTTCGGTTCAATATGTTATATTTGATGTTAATTTGGGTTGGTTATTTCGGATTTTTCATTTTAATGGGGCGAGTTTGTTTTTTATTTTTTTGTATTTGCATATTTTTAAGGGTTTGTTTATGGTTAGTTATCGGTTGAAAAAAGTTTGAGGTACGGGTTTATTGATTTTTTTGTTGATTATGATAGAGGCTTTTATGGGTTATGTTTTGGTCTGGGCTCAAATAAGATTTTGAGCTGCGGTTGTAATTACTAGGTTGTTGAGTGTTGTTCCTGTTTGAGGTCAATTGATTGTAATGTGAATTTGAAGAGGTTTTGGAGTAACCAGGTCAACTTTAAAGTTTTTTTTT---GTTTTACATTTTTTGTTGCCTTGGCTATTGTTGGTTTTGGTTATGGTGCATATGATTTTTTTGCATAGTACTGGTAGAACTTCTAGTTTGTATTGTCATGGTGATTATGATAAAATTAGTTTTGGTCCTTATTATTGGAATAAGGATTTTTATAATTTTTTAGTTTTTTTTGTTTTTTTTTTGTTAGTTTTATTGAAGCCTTTTTATTTAGGGGATCCCGAAATGTTTGTTGAGGCAGATCCCATGATGAGCCCAGTACATATTGTTCCAGAATGGTATTTTTTGTTTGCTTATGCTATTTTGCGGGCTATTCCTAATAAGGTTATGGGTGTTGTAGCTTTGTTGATAAGAATTTTGTTATTTTATTTTTTTTTATTGGTTAGTAATTATACGTCTTGTTTGG--

>H8

-GTTGAAATTATGGTAGAATGTTGGGAATAATTTTAGTTTTTCAGTTGTTAACTGGATTATTTTTATCTTTTTATTATGTTGCGGATGGTTTGGTGGCTTTTAGTTCGGTTCAATATATTATATTTGATGTTAATTTGGGTTGGTTATTTCGGATTTTTCATTTTAATGGGGCGAGTTTGTTTTTTATTTTTTTGTATTTGCATATTTTTAAGGGTTTGTTTATGGTTAGTTATCGGTTAAAAAAGGTTTGAGGTACGGGTTTGTTGATTTTTTTGTTGATTATGATAGAGGCTTTTATGGGTTATGTTTTAGTTTGGGCTCAAATAAGATTTTGAGCCGCGGTTGTAATTACTAGGTTGTTGAGTGTTGTTCCTGTTTGAGGTCAATTGATTGTGATGTGAATTTGAAGAGGTTTTGGAGTAACAAGGTCAACTTTAAAGTTTTTTTTT---GTTTTGCATTTTTTGTTACCTTGGTTATTGTTGGTTTTGGTTATAGTGCATATGATTTTTTTGCATAGTACTGGTAGAACTTCTAGTTTGTATTGTCATGGAGATTATGATAAAATTAGTTTTGGTCCTTATTATTGGAATAAGGACTTTTATAATTTTTTAGTTTTTTTTGTTTTTTTTTTGTTAGTTTTATTGAAGCCTTTTTATTTAGGGGATCCTGAAATGTTTGTTGAGGCAGATCCTATGATGAGCCCAGTGCATATTGTTCCAGAATGGTATTTTTTGTTTGCTTATGCTATTTTGCGGGCTATTCCTAATAAGGTTATGGGTGTTGTAGCTTCGTTGATAAGAATTTTGTTATTTTATTTTTTTTTATTGGTTAGTAATTATACGTCTTGTTTGG--

>H9

-GTTGAAATTATGGTAGAATGTTGGGAATAATTTTAGTTTTTCAGTTGTTAACTGGATTATTTTTATCTTTTTATTATGTTGCGGATGGTTTGGTGGCTTTTAGTTCGGTTCAATATATTATATTTGATGTTAATTTGGGTTGGTTATTTCGGATTTTTCATTTTAATGGGGCGAGTTTGTTTTTTATTTTTTTGTATTTGCATATTTTTAAGGGTTTGTTTATGGTTAGTTATCGGTTAAAAAAGGTTTGAGGTACGGGTTTGTTGATTTTTTTGTTGATTATGATAGAGGCTTTTATGGGTTATGTTTTAGTTTGGGCTCAAATAAGATTTTGAGCCGCGGTTGTAATTACTAGGTTGTTGAGTGTTGTTCCTGTTTGAGGTCAATTGATTGTGATGTGAATTTGAAGAGGTTTTGGAGTAACAAGGTCAACTTTAAAGTTTTTTTTT---GTTTTGCATTTTTTGTTACCTTGGTTATTGTTGGTTTTGGTTATAGTGCATATGATTTTTTTGCATAGTACTGGTAGAACTTCTAGTTTGTATTGTCATGGAGATTATGATAAAATTAGTTTTGGTCCTTATTATTGGAATAAGGACTTTTATAATTTTTTAGTTTTTTTTGTTTTTTTTTTGTTAGTTTTATTGAAGCCTTTTTATTTAGGGGATCCTGAAATGTTTGTTGAGGCAGATCCTATGATGAGCCCAGTGCATATTGTTCCAGAATGGTATTTTTTGTTTGCTTATGCTATTTTGCGGGCTATTCCTAATAAGGTTATGGGTGTTGTAGCTTTGTTGATAAGAATTTTGTTATTTTATTTTTTTTTATTGGTTAGTAATTATACGTCTTGTTTGG--

>H10

-GTTGAAATTATGGTAGAATGTTGGGAATAATTTTAGTTTTTCAATTGTTAACTGGATTATTTTTATCTTTTTATTATGTTGCGGATGGTTTGGTGGCTTTTAGTTCGGTTCAATATATTATATTTGATGTTAATTTGGGTTGGTTATTTCGGATTTTTCATTTTAATGGGGCGAGTTTGTTTTTTATTTTTTTGTATTTGCATATTTTTAAGGGTTTGTTTATGGTTAGTTATCGGTTAAAAAAGGTTTGAGGTACGGGTTTGTTGATTTTTTTGTTGATTATGATAGAGGCTTTTATGGGTTATGTTTTAGTTTGGGCTCAAATAAGATTTTGAGCCGCGGTTGTAATTACTAGGTTGTTGAGTGTTGTTCCTGTTTGAGGTCAATTGATTGTGATGTGAATTTGAAGAGGTTTTGGAGTAACAAGGTCAACTTTAAAGTTTTTTTTT---GTTTTGCATTTTTTGTTACCTTGGTTATTGTTGGTTTTGGTTATAGTGCATATGATTTTTTTGCATAGTACTGGTAGAACTTCTAGTTTGTATTGTCATGGAGATTATGATAAAATTAGTTTTGGTCCTTATTATTGGAATAAGGACTTTTATAATTTTTTAGTTTTTTTTGTTTTTTTTTTGTTAGTTTTATTGAAGCCTTTTTATTTAGGGGATCCTGAAATGTTTGTTGAAGCAGATCCTATGATGAGCCCAGTGCATATTGTTCCAGAATGGTATTTTTTGTTTGCTTATGCTATTTTGCGGGCTATTCCTAATAAGGTTATGGGTGTTGTAGCTTTGTTGATAAGAATTTTGTTATTTTATTTTTTTTTATTGGTTAGTAATTATACGTCTTGTTTGG--

>H11

-GTTGAAATTATGGTAGAATGTTGGGAATAATTTTAGTTTTTCAATTGTTAACTGGATTATTTTTATCTTTTTATTATGTTGCGGATGGTTTGGTGGCTTTTAGTTCGGTTCAATATATTATATTTGATGTTAATTTGGGTTGGTTATTTCGGATTTTTCATTTTAATGGGGCGAGTTTGTTTTTTATTTTTTTGTATTTGCATATTTTTAAGGGTTTGTTTATGGTTAGTTATCGGTTAAAAAAGGTTTGAGGTACGGGTTTGTTGATTTTTTTGTTGATTATGATAGAGGCTTTTATGGGTTATGTTTTAGTTTGGGCTCAAATAAGATTTTGAGCCGCGGTTGTAATTACTAGGTTGTTGAGTGTTGTTCCTGTTTGAGGTCAATTGATTGTGATGTGAATTTGAAGAGGTTTTGGAGTAACAAGGTCAACTTTAAAGTTTTTTTTT---GTTTTGCATTTTTTGTTACCTTGGTTATTGTTGGTTTTGGTTATAGTGCATATGATTTTTTTGCATAGTACTGGTAGAACTTCTAGTTTGTATTGTCATGGAGATTATGATAAAATTAGTTTTGGTCCTTATTATTGGAATAAGGACTTTTATAATTTTTTAGTTTTTTTTGTTTTTTTTTTGTTAGTTTTATTGAAGCCTTTTTATTTAGGGGATCCTGAAATGTTTGTTGAGGCAGATCCTATGATGAGCCCAGTGCATATTGTTCCAGAATGGTATTTTTTGTTTGCTTATGCTATTTTGCGGGCTATTCCTAATAAGGTTATGGGTGTTGTAGCTTTGTTGATAAGAATTTTGTTATTTTATTTTTTTTTATTGGTTAGTAATTATACGTCTTGTTTGG--

>H12

-GTTGAAATTATGGTAGAATGTTGGGAATAATTTTGGTTTTTCAATTGTTAACTGGTTTATTTTTATCTTTTTATTATGTTGCAGATGGTTTAGTAGCTTTTAGTTCGGTTCAATATATTATATCTGATGTTAATTTGGGTTGGTTGTTTCGGATTTTTCATTTTAATGGGGCAAGTTTGTTTTTTATTTTTTTGTATTTGCATATTTTTAAAGGTTTGTTTATGGTTAGTTATCGGTTAAAAAAAGTTTGAGGTACTGGTTTATTAATTTTTTTGCTAGTTATGATGGAGGCTTTTATAGGTTATGTTTTAGTTTGAGCTCAAATAAGATTTTGAGCTGCGGTTGTAATTACTAGGTTGTTGAGTGTCGTTCCTGTTTGAGGTCAGTTGATTGTGATGTGAATTTGAAGAGGTTTTGGGGTAACAAGGTCAACTTTAAAATTTTTTTTT---GTTTTACATTTTTTATTACCTTGGTTATTGTTGGTTTTGGTTGTAGTGCATATGATTTTTTTACATAGTACTGGTAGAACTTCTAGTTTGTATTGTCATGGTGATTATGATAAAATTAGTTTTGGTCCTTATTATTGGAATAAAGACTTTTATAATTTATTAGTTTTTTTTGTTTTTTTTTTGTTAGTTTTGTTGAAACCTTTTTATTTGGGAGATCCTGAAATGTTTGTTGAGGCAGATCCTATGATGAGACCGGTACATATTGTTCCAGAGTGATATTTTTTGTTTGCTTATGCTATTTTGCGGGCTATCCCTAATAAGGTTATGGGTGTTGTAGCTTTGTTGATAAGAATTTTGTTATTTTATTTTTTTTTATTGGTTAGTAATTATACATCTTGTTTGG--

>AP017672 A. cantonensis

-GTTGAAATTATGGTAGAATGTTGGGAATAATTTTAGTTTTTCAGTTGTTAACTGGATTATTTTTATCTTTTTATTATGTTGCGGATGGTTTAGTGGCTTTTAGTTCGGTTCAATATGTTATATTTGATGTTAATTTGGGTTGGTTATTTCGGATTTTTCATTTTAATGGGGCGAGTTTGTTTTTTATTTTTTTGTATTTGCATATTTTTAAGGGTTTGTTTATGGTTAGTTATCGGTTGAAAAAAGTTTGAGGTACGGGTTTATTGATTTTTTTGTTGATTATGATAGAGGCTTTTATGGGTTATGTTTTAGTCTGGGCTCAAATAAGATTTTGAGCTGCGGTTGTAATTACTAGGTTGTTGAGTGTTGTTCCTGTTTGAGGTCAATTGATTGTAATGTGAATTTGAAGAGGTTTTGGAGTAACTAGGTCAACTTTAAAGTTTTTTTTT---GTTTTACATTTTTTGTTGCCTTGGCTATTGTTGGTTTTGGTTATAGTGCATATGATTTTTTTGCATAGTACTGGTAGAACTTCTAGTTTGTATTGTCATGGTGATTATGATAAAATTAGTTTTGGTCCTTATTATTGGAATAAGGATTTTTATAATTTTTTAGTTTTTTTTGTTTTTTTTTTGTTAGTTTTATTGAAGCCTTTTTATTTAGGGGATCCCGAAATGTTTGTTGAGGCAGATCCCATGATGAGCCCAGTGCATATTGTTCCAGAATGGTATTTTTTGTTTGCTTATGCTATTTTGCGGGCTATTCCCAATAAGGTTATGGGTGTTGTAGCTTTGTTGATAAGAATTTTGTTATTTTATTTTTTTTTATTGGTTAGTAATTATACGTCTTGTTTGG--

>NC013065 A. cantonensis

-GTTGAAATTATGGTAGAATGTTGGGAATAATTTTAGTTTTTCAGTTGTTAACTGGACTATTTTTATCTTTTTATTATGTTGCGGATGGTTTAGTGGCTTTTAGTTCGGTTCAGTATATTATATCTGATGTTAATTTGGGTTGGTTATTTCGAATTTTTCATTTTAATGGGGCGAGTTTGTTTTTTATTTTTTTGTATTTGCATATTTTTAAGGGTTTGTTTATAGTTAGTTATCGGTTAAAAAAAGTTTGAGGTACGGGTTTATTGATTTTTTTGTTGATTATGATAGAGGCTTTTATGGGTTATGTTTTAGTTTGGGCTCAAATAAGATTTTGAGCTGCGGTTGTAATTACTAGGTTGTTGAGTGTTGTTCCTGTTTGAGGCCAACTGATTGTGATGTGAATTTGAAGAGGTTTTGGAGTAACAAGGTCAACTTTAAAGTTTTTTTTT---GTTTTGCATTTTTTGTTACCTTGATTATTATTAGTTTTGGTTATAGTGCATATGATTTTTTTGCATAGTACCGGTAGAACTTCTAGTTTGTATTGTCATGGTGATTATGATAAAATTAGTTTTGGTCCTTATTATTGGAATAAGGACTTTTATAATTTGTTAGTTTTTTTTGTTTTTTTTTTGTTAGTTCTATTGAAACCTTTTTATTTAGGAGATCCTGAAATGTTTGTTGAGGCTGACCCCATGATGAGTCCAGTGCATATTGTCCCAGAATGGTATTTTTTGTTTGCTTATGCTATTTTGCGGGCTATTCCTAATAAGGTTATGGGTGTTGTAGCTTCGTTGATAAGAATTTTGTTATTTTATTTTTTTTTACTGGTTAGTAATTATACGTCTTGTTTGG--

>H13

-GTTGAAATTATGGTAGAATACTGGGGATAATTTTGATTTTTCAGTTATTAACTGGGTTGTTTTTATCTTTTTATTATGTAGCTGATGGTTTGGTGGCTTTTAGTTCAGTTCAGTATATTATATATGATATTAATTTGGGTTGATTATTTCGGATTTTTCATTTTAATGGGGCAAGTTTGTTTTTTATTTTTTTGTATTTACATATTTTTAAAGGTTTATTTATAGTTAGTTATCGGTTGAAAAAGGTTTGAGGTACAGGTTTGTTGATTTTTTTATTGATTATAATAGAGGCTTTTATAGGATATGTTTTGGTTTGGGCTCAAATAAGATTTTGAGCAGCGGTTGTAATCACCAGATTATTGAGTGTTATTCCTGTTTGGGGACAATTAATTGTAATATGAGTTTGAAGGGGTTTCAGGGTTACAAGATCGACTTTAAAATTTTTTTTT---GTTTTACATTTTTTATTACCTTGGTTGCTGATAGTTTTGGTTATGATGCATATGGTTTTTTTGCATAGAACTGGTAGAACTTCTAGTTTATATTGTCATGGTGATTATGATAAAATTAGTTTTGGTCCTTATTATTGGAATAAAGATTTTTATAATTTATTAGTTTTTTTTATTTTTTTCTTATTAGTTTTGTTGAAACCTTTTTATTTAGGTGATCCCGAGATATTTATTGAGGCTGATCCTTTGACGAGACCAGTACATATTGTCCCAGAGTGATATTTTTTATTTGCTTATGCTATTTTGCGGGCTATTCCTAATAAAGTTTTGGGTGTCTTGGCTTTATTGATGAGGGTTTTGTCATTTTATTTTTTTTTATTATTTAATAATTATACGTCTTGTTTGG--

>KT947979 A. malaysiensis

-GTTGAAATTATGGTAGAATACTGGGGATAATTTTGATTTTTCAGTTATTAACTGGGTTGTTTTTATCTTTTTATTATGTAGCTGATGGTTTGGTGGCTTTTAGTTCAGTTCAGTATATTATATATGATATTAATTTGGGTTGATTATTTCGGATTTTTCATTTTAATGGGGCAAGTTTGTTTTTTATTTTTTTGTATTTACATATTTTTAAAGGTTTATTTATAGTTAGTTATCGGTTGAAAAAGGTTTGAGGTACAGGTTTGTTGATTTTTTTATTGATTATAATAGAGGCTTTTATAGGATATGTTTTGGTTTGGGCTCAAATAAGATTTTGAGCAGCGGTTGTAATCACCAGATTATTGAGTGTTATTCCTGTTTGGGGACAATTAATTGTAATATGAGTTTGAAGGGGTTTCAGGGTTACAAGATCGACTTTAAAATTTTTTTTT---GTTTTACATTTTTTATTACCTTGGTTGCTGATAGTTTTGGTTATGATGCATATGGTTTTTTTGCATAGAACTGGTAGAACTTCTAGTTTATATTGTCATGGTGATTATGATAAAATTAGTTTTGGTCCTTATTATTGGAATAAAGATTTTTATAATTTATTAGTTTTTTTTATTTTTTTCTTATTAGTTTTGTTGAAACCTTTTTATTTAGGTGATCCCGAGATATTTATTGAGGCTGATCCTTTGACGAGACCAGTACATATTGTTCCAGAGTGATATTTTTTATTTGCTTATGCTATTTTGCGGGCTATTCCTAATAAAGTTTTGGGTGTCTTGGCTTTATTGATGAGGGTTTTGTCATTTTATTTTTTTTTATTATTTAATAATTATACGTCTTGTTTGG--

>H15

-GTTGAAATTATGGTAGAATACTGGGGATAATTTTGATTTTTCAGTTATTAACTGGGTTGTTTTTATCTTTTTATTATGTAGCTGATGGTTTGGTGGCTTTTAGTTCAGTTCAGTATATTATATATGATATTAATTTGGGTTGATTATTTCGGATTTTTCATTTTAATGGGGCAAGTTTGTTTTTTATTTTTTTGTATTTACATATTTTTAAAGGTTTATTTATAGTTAGTTATCGGTTGAAAAAGGTTTGAGGTACAGGTTTGTTGATTTTTTTATTGATTATAATAGAGGCTTTTATAGGATATGTTTTGGTTTGGGCTCAAATAAGATTTTGAGCAGCGGTTGTAATCACCAGATTATTGAGTGTTATTCCTGTTTGGGGACAATTAATTGTAATATGAGTTTGAAGGGGTTTCAGGGTTACAAGATCGACTTTAAAATTTTTTTTT---GTTTTACATTTTTTATTACCTTGGTTGCTGATAGTTTTGGTTATGATGCATATGGTTTTTTTGCATAGAACTGGTAGAACTTCTAGTTTATATTGTCATGGTGATTATGATAAAATTAGTTTTGGTCCTTATTATTGGAATAAAGATTTTTATAATTTGTTAGTTTTTTTTATTTTTTTCTTATTAGTTTTGTTGAAACCTTTTTATTTAGGTGATCCCGAGATATTTATTGAGGCTGATCCTTTGACGAGACCAGTACATATTGTTCCAGAGTGATATTTTTTATTTGCTTATGCTATTTTGCGGGCTATTCCTAATAAAGTTTTGGGTGTTTTGGCTTTATTGATGAGGGTTTTGTCATTTTATTTTTTTTTATTATTTAATAATTATACGTCTTGTTTGG--

>H16

-GTTGAAATTATGGTAGAATATTGGGGATAATTTTGATTTTTCAGTTATTAACTGGGTTGTTTTTATCTTTTTATTATGTAGCTGATGGTTTGGTGGCTTTTAGTTCAGTTCAGTATATTATATATGATATTAATTTGGGTTGATTATTTCGGATTTTTCATTTTAATGGGGCAAGTTTGTTTTTTATTTTTTTGTATTTACATATTTTTAAAGGTTTATTTATAGTTAGTTATCGGTTGAAAAAGGTTTGAGGTACAGGTTTGTTGATTTTTTTATTGATTATAATAGAGGCTTTTATAGGATATGTTTTGGTTTGGGCTCAAATAAGATTTTGAGCAGCGGTTGTAATCACCAGATTATTGAGTGTTATTCCTGTTTGGGGACAATTAATTGTAATATGAGTTTGAAGGGGTTTCAGGGTTACAAGATCGACTTTAAAATTTTTTTTT---GTTTTACATTTTTTATTACCTTGGTTGCTGATAGTTTTGGTTATGATGCATATGGTTTTTTTGCATAGAACTGGTAGAACTTCTAGTTTATATTGTCATGGTGATTATGATAAAATTAGTTTTGGTCCTTATTATTGGAATAAAGATTTTTATAATTTATTAGTTTTTTTTATTTTTTTCTTATTAGTTTTGTTGAAACCTTTTTATTTAGGTGATCCCGAGATATTTATTGAGGCTGATCCTTTGACGAGACCAGTACATATTGTCCCAGAGTGATATTTTTTATTTGCTTATGCTATTTTGCGGGCTATTCCTAATAAAGTTTTGGGTGTCTTGGCTTTATTGATGAGGGTTTTGTCATTTTATTTTTTTTTATTATTTAATAATTATACGTCTTGTTTGG--

>H17

-GTTGAAATTATGGTAGAATACTGGGGATAATTTTGATTTTTCAGTTATTAACTGGGTTGTTTTTATCTTTTTATTATGTAGCTGATGGTTTGGTGGCTTTTAGTTCAGTTCAGTATATTATATATGATATTAATTTGGGTTGATTATTTCGGATTTTTCATTTTAATGGGGCAAGTTTGTTTTTTATTTTCTTGTATTTACATATTTTTAAAGGTTTATTTATAGTTAGTTATCGGTTGAAAAAGGTTTGAGGTACAGGTTTGTTGATTTTTTTATTGATTATAATAGAGGCTTTTATAGGATATGTTTTGGTTTGGGCTCAAATAAGATTTTGAGCAGCGGTTGTAATCACCAGATTATTGAGTGTTATTCCTGTTTGGGGACAATTAATTGTAATATGAGTTTGAAGGGGTTTCAGGGTTACAAGATCGACTTTAAAATTTTTTTTT---GTTTTACATTTTTTATTACCTTGGTTGCTGATAGTTTTGGTTATGATGCATATGGTTTTTTTGCATAGAACTGGTAGAACTTCTAGTTTATATTGTCATGGTGATTATGATAAAATTAGTTTTGGTCCTTATTATTGGAATAAAGATTTTTATAATTTATTAGTTTTTTTTATTTTTTTCTTATTAGTTTTGTTGAAACCTTTTTATTTAGGTGATCCCGAGATATTTATTGAGGCTGATCCTTTGACGAGACCAGTACATATTGTTCCAGAGTGATATTTTTTATTTGCTTATGCTATTTTGCGGGCTATTCCTAATAAAGTTTTGGGTGTCTTGGCTTTATTGATGAGGGTTTTGTCATTTTATTTTTTTTTATTATTTAATAATTATACGTCTTGTTTGG--

>H18

-GTTGAAATTATGGTAGAATACTGGGGATAATTTTGATTTTTCAGTTATTAACTGGGTTGTTTTTATCTTTTTATTATGTAGCTGATGGTTTGGTGGCTTTTAGTTCAGTTCAGTATATTATATATGATATTAATTTGGGTTGATTATTTCGGATTTTTCATTTTAATGGGGCAAGTTTGTTTTTTATTTTTTTGTATTTACATATTTTTAAAGGTTTATTTATAGTTAGTTATCGGTTGAAAAAGGTTTGAGGTACAGGTTTGTTGATTTTTTTATTGATTATAATAGAGGCTTTTATAGGATATGTTTTGGTTTGGGCTCAAATAAGATTTTGAGCAGCGGTTGTAATCACCAGATTATTGAGTGTTATTCCTGTTTGGGGACAATTAATTGTAATATGAGTTTGAAGGGGTTTCAGGGTTACAAGATCGACTTTAAAATTTTTTTTT---GTTTTACATTTTTTATTACCTTGGTTGCTGATAGTTTTGGTTATGATGCATATGGTTTTTTTGCATAGAACTGGTAGAACTTCTAGTTTATATTGTCATGGTGATTATGATAAAATTAGTTTTGGTCCTTATTATTGGAATAAAGATTTTTATAATTTGTTAGTTTTTTTTATTTTTTTCTTATTAGTTTTGTTGAAACCTTTTTATTTAGGTGATCCCGAGATATTTATTGAGGCTGATCCTTTGACGAGACCAGTACATATTGTTCCAGAGTGATATTTTTTATTTGCTTATGCTATTTTGCGGGCTATTCCTAATAAAGTTTTGGGTGTCTTGGCTTTATTGATGAGGGTTTTGTCATTTTATTTTTTTTTATTATTTAATAATTATACGTCTTGTTTGG--

>H19

-GTTGAAATTATGGTAGAATACTGGGGATAATTTTGATTTTTCAGTTATTAACTGGATTGTTTTTATCTTTTTATTATGTAGCTGATGGTTTGGTGGCTTTTAGTTCAGTTCAGTATATTATATATGATATTAATTTGGGTTGATTATTTCGGATTTTTCATTTTAATGGGGCAAGTTTGTTTTTTATTTTTTTGTATTTACATATTTTTAAAGGTTTATTTATAGTTAGTTATCGGTTGAAAAAGGTTTGAGGTACAGGTTTGTTGATTTTTTTATTGATTATAATAGAGGCTTTTATAGGATATGTTTTGGTTTGGGCTCAAATAAGATTTTGAGCAGCAGTTGTAATCACCAGATTATTGAGTGTTATTCCTGTTTGGGGACAATTAATTGTAATATGAGTTTGAAGGGGTTTCAGGGTTACAAGATCGACTTTAAAATTTTTTTTT---GTTTTGCATTTTTTATTACCTTGGTTGCTGATAGTTTTGGTTATGATGCATATGGTTTTTTTGCATAGAACTGGTAGAACTTCTAGTTTATATTGTCATGGTGATTATGATAAAATTAGTTTTGGTCCTTATTATTGGAATAAAGATTTTTATAATTTGTTAGTTTTTTTTATTTTTTTCTTATTAGTTTTGTTAAAACCTTTTTATTTAGGTGATCCCGAGATATTTATTGAGGCTGATCCTTTGACGAGACCAGTACATATTGTTCCAGAGTGATATTTTTTATTTGCTTATGCTATTTTGCGGGCCATTCCTAATAAAGTTTTGGGTGTCTTGGCTTTATTGATGAGGGTTTTGTCATTTTATTTTTTTTTATTATTTAATAATTATACATCTTGTTTGG--

>H20

-GTTGAAATTATGGTAGAATACTGGGGATAATTTTGATTTTTCAGTTATTAACTGGATTGTTTTTATCTTTTTATTATGTAGCTGATGGTTTGGTGGCTTTTAGTTCAGTTCAGTATATTATATATGATATTAATTTGGGTTGATTATTTCGGATTTTTCATTTTAATGGGGCAAGTTTGTTTTTTATTTTTTTGTATTTACATATTTTTAAAGGTTTATTTATAGTTAGTTATCGGTTGAAAAAGGTTTGAGGTACAGGTTTGTTGATTTTTTTATTGATTATAATAGAGGCTTTTATAGGATATGTTTTGGTTTGGGCTCAAATAAGATTTTGAGCAGCAGTTGTAATCACCAGATTATTGAGTGTTATTCCTGTTTGGGGACAATTAATTGTAATATGAGTTTGAAGGGGTTTCAGGGTTACAAGATCGACTTTAAAATTTTTTTTT---GTTTTGCATTTTTTATTACCTTGGTTGTTGATAGTTTTGGTTATGATGCATATGGTTTTTTTGCATAGAACTGGTAGAACTTCTAGTTTATATTGTCATGGTGATTATGATAAAATTAGTTTTGGTCCTTATTATTGGAATAAAGATTTTTATAATTTGTTAGTTTTTTTTATTTTTTTCTTATTAGTTTTGTTAAAACCTTTTTATTTAGGTGATCCCGAGATATTTATTGAGGCTGATCCTTTGACGAGACCAGTACATATTGTTCCAGAGTGATATTTTTTATTTGCTTATGCTATTTTGCGGGCCATTCCTAATAAAGTTTTGGGTGTCTTGGCTTTATTGATGAGGGTTTTGTCATTTTATTTTTTTTTATTATTTAATAATTATACATCTTGTTTGG--

>H21

-GTTGAAATTATGGTAGAATACTGGGGATAATTTTGATTTTTCAGTTATTAACTGGGTTGTTTTTATCTTTTTATTATGTAGCTGATGGTTTGGTGGCTTTTAGTTCAGTTCAGTATATTATATATGATATTAATTTGGGTTGATTATTTCGGATTTTTCATTTTAATGGGGCAAGTTTGTTTTTTATTTTTTTGTATTTACATATTTTTAAAGGTTTATTTATAGTTAGTTATCGGTTGAAAAAGGTTTGAGGTACAGGTTTGTTGATTTTTTTATTGATTATAATAGAGGCTTTTATAGGATATGTTTTGGTTTGGGCTCAAATAAGATTTTGAGCAGCGGTTGTAATCACCAGATTATTGAGTGTTATTCCTGTTTGGGGACAATTAATTGTAATATGAGTTTGAAGGGGTTTCAGGGTTACAAGATCGACTTTAAAATTTTTTTTT---GTTTTACATTTTTTATTACCTTGGTTGCTGATAGTTTTGGTTATGATGCATATGGTTTTTTTGCATAGAACCGGTAGAACTTCTAGTTTATATTGTCATGGTGATTATGATAAAATTAGTTTTGGTCCTTATTATTGGAATAAAGATTTTTATAATTTGTTAGTTTTTTTTATTTTTTTCTTATTAGTTTTGTTGAAACCTTTTTATTTAGGTGATCCCGAGATATTTATTGAGGCTGATCCTTTGACGAGACCAGTACATATTGTTCCAGAGTGATATTTTTTATTTGCTTATGCTATTTTGCGGGCTATTCCTAATAAAGTTTTGGGTGTCTTGGCTTTATTGATGAGGGTTTTGTCATTTTATTTTTTTTTATTATTTAATAATTATACGTCTTGTTTGG--

>KT186242 A. cantonensis

-GTTGAAATTATGGTAGAATACTGGGGATAATTTTGATTTTTCAGTTATTAACTGGGTTGTTTTTATCTTTTTATTATGTAGCTGATGGTTTGGTGGCTTTTAGTTCAGTTCAGTATATTATATATGATATTAATTTGGGTTGATTATTTCGGATTTTTCATTTTAATGGGGCAAGTTTGTTTTTTATTTTTTTGTATTTACATATTTTTAAAGGTTTATTTATAGTTAGTTATCGGTTGAAAAAGGTTTGAGGTACAGGTTTGTTGATTTTTTTATTGATTATAATAGAGGCTTTTATAGGATATGTTTTGGTTTGGGCTCAAATAAGATTTTGAGCAGCGGTTGTAATCACCAGATTATTGAGTGTTATTCCTGTTTGGGGACAATTAATTGTAATATGAGTTTGAAGGGGTTTCAGGGTTACAAGATCGACTTTAAAATTTTTTTTT---GTTTTACATTTTTTATTACCTTGGTTGCTGATAGTTTTGGTTATGATGCATATGGTTTTTTTGCATAGAACTGGTAGAACTTCTAGTTTATATTGTCATGGTGATTATGATAAAATTAGTTTTGGTCCTTATTATTGGAATAAAGATTTTTATAATTTATTAGTTTTTTTTATTTTTTTCTTATTAGTTTTGTTAAAACCTTTTTATTTAGGTGATCCCGAGATATTTATTGAGGCTGATCCTTTGACGAGACCAGTACATATTGTTCCAGAGTGATATTTTTTATTTGCTTATGCTATTTTGCGGGCTATTCCTAATAAAGTTTTGGGTGTCTTGGCTTTATTGATGAGGGTTTTGTCATTTTATTTTTTTTTATTATTTAATAATTATACGTCTTGTTTGG--

>KP721447

-GTTGAAATTATGGTAGAATGTTGGGAATAATTTTAGTTTTTCAGTTGTTAACTGGATTATTTTTATCTTTTTATTATGTTGCGGATGGTTTAGTGGCTTTTAGTTCGGTTCAATATGTTATATTTGATGTTAATTTGGGTTGGTTATTTCGGATTTTTCATTTTAATGGGGCGAGTTTGTTTTTTATTTTTTTGTATTTGCATATTTTTAAGGGTTTGTTTATAGTTAGTTATCGGTTGAAAAAAGTTTGAGGTACGGGTTTATTGATTTTTTTGTTGATTATGATAGAGGCTTTTATGGGTTATGTTTTAGTTTGGGCTCAAATAAGATTTTGAGCTGCGGTTGTAATTACTAGGTTGTTGAGTGTTGTTCCTGTTTGAGGTCAGTTGATTGTAATGTGAATTTGAAGAGGTTTTGGAGTAACCAGGTCAACTTTAAAGTTTTTTTTT---GTTTTACATTTTTTGTTACCTTGGCTATTGTTGGTTTTGGTTATAGTGCATATGATTTTTTTGCATAGTACTGGTAGAACTTCTAGTTTGTATTGTCATGGTGATTATGATAAAATTAGTTTTGGTCCTTATTATTGGAATAAGGATTTTTATAATTTTTTAGTTTTTTTTGTTTTTTTTTTGTTAGTTTTGTTGAAGCCTTTTTATTTAGGAGATCCCGAAATGTTTGTTGAGGCAGATCCCATGGTGAGCCCAGTGCATATTGTTCCAGAATGGTATTTTTTGTTTGCTTATGCTATTCTGCGAGCTATTCCTAATAAGGTTATGGGTGTTGTAGCTTTGTTGATAAGAATTTTGTTATTTTATTTTTTTTTGTTGGTTAGTAATTATACGTCTTGTTTGG--

>KP721449

-GTTGAAATTATGGTAGAATGTTGGGAATAATTTTAGTTTTTCAGTTGTTAACTGGATTATTTTTATCTTTTTATTATGTTGCGGATGGTTTAGTGGCTTTTAGTTCGGTTCAATATGTTATATTTGATGTTAATTTGGGTTGGTTATTTCGGATTTTTCATTTTAATGGGGCGAGTTTGTTTTTTATTTTTTTGTATTTGCATATTTTTAAGGGTTTGTTTATAGTTAGTTATCGGTTGAAAAAAGTTTGAGGTACGGGTTTATTGATTTTTTTGTTGATTATGATAGAGGCTTTTATGGGTTATGTTTTAGTTTGGGCTCAAATAAGATTTTGAGCTGCGGTTGTAATTACTAGGTTGTTGAGTGTTGTTCCTTTTTGAGGTCAGTTGATTGTAATGTGAATTTGAAGAGGTTTTGGAGTAACCAGGTCAACTTTAAAGTTTTTTTTT---GTTTTACATTTTTTGTTACCTTGGCTATTGTTGGTTTTGGTTATAGTGCATATGATTTTTTTGCATAGTACTGGTAGAACTTCTAGTTTGTATTGTCATGGTGATTATGATAAAATTAGTTTTGGTCCTTATTATTGGAATAAGGATTTTTATAATTTTTTAGTTTTTTTTGTTTTTTTTTTGTTAGTTTTGTTGAAGCCTTTTTATTTAGGAGATCCCGAAATGTTTGTTGAGGCAGATCCCATGGTGAGCCCAGTGCATATTGTTCCAGAATGGTATTTTTTGTTTGCTTATGCTATTTTGCGAGCTATTCCTAATAAGGTTATGGGTGTTGTAGCTTTGTTGATAAGAATTTTGTTATTTTATTTTTTTTTGTTGGTTAGTAATTATACGTCTTGTTTGG--

>KP721450

-GTTGGAATTATGGTAGAATGTTGGGGATAATTTTAGTTTTTCAGTTGTTAACTGGATTATTTTTATCTTTTTATTATGTTGCGGATGGTTTAGTGGCTTTTAGTTCGGTTCAATATGTTATATTTGATGTTAATTTGGGTTGGTTATTTCGGATTTTTCATTTTAATGGGGCGAGTTTGTTTTTTATTTTTTTGTATTTGCATATTTTTAAGGGTTTGTTTATGGTTAGTTATCGGTTGAAAAAAGTTTGAGGTACGGGTTTATTGATTTTTTTGTTGATTATGATAGAGGCTTTTATGGGTTATGTTTTGGTCTGGGCTCAAATAAGATTTTGAGCTGCGGTTGTAATTACTAGGTTGTTGAGTGTTGTTCCTGTTTGAGGTCAATTGATTGTAATGTGAATTTGAAGAGGTTTTGGAGTAACCAGGTCAACTTTGAAGTTTTTTTTT---GTTTTACATTTTTTGTTGCCTTGGCTATTGTTGGTTTTGGTTATGGTGCATATGATTTTTTTGCATAGTACTGGTAGAACTTCTAGTTTGTATTGTCATGGTGATTATGATAAAATTAGTTTTGGTCCTTATTATTGGAATAAGGATTTTTATAATTTTTTAGTTTTTTTTGTTTTTTTTTTGTTAGTTTTATTGAAGCCTTTTTATTTAGGGGATCCCGAAATGTTTGTTGAGGCAGATCCCATGATGAGCCCAGTACATATTGTTCCAGAATGGTATTTTTTGTTTGCTTATGCTATTTTGCGGGCTATTCCTAATAAGGTTATGGGTGTTGTAGCTTTGTTGATAAGAATTTTGTTATTTTATTTTTTTTTATTGGTTAGTAATTATACGTCTTGTTTGG--

>KP721454

-GTTGAAATTATGGTAGAATGTTGGGAATAATTTTAGTTTTTCAGTTGTTAACTGGATTATTTTTATCTTTTTATTATGTTGCGGATGGTTTAGTGGCTTTTAGTTCGGTTCAGTATATTATATTTGATGTTAATTTGGGTTGGTTATTTCGAATTTTTCATTTTAATGGGGCGAGTTTGTTTTTTATTTTTTTGTATTTGCATATTTTTAAGGGTTTGTTTATAGTTAGTTATCGGTTAAAAAAAGTTTGAGGTACGGGTTTATTGATTTTTTTGTTGATTATGATAGAGGCTTTTATGGGTTATGTTTTAGTTTGGGCTCAAATAAGATTTTGAGCTGCGGTTGTAATTACTAGGTTGTTGAGTGTTGTTCCTGTTTGAGGTCAACTGATTGTGATGTGAATTTGAAGAGGTTTTGGAGTAACAAGGTCAACTTTAAAGTTTTTTTTT---GTTTTGCATTTTTTGTTACCTTGATTATTATTAGTTTTGGTTATAGTGCATATGATTTTTTTGCATAGTACCGGTAGAACTTCTAGTTTGTATTGTCATGGTGATTATGATAAAATTAGTTTTGGTCCTTATTATTGGAATAAGGACTTTTATAATTTGTTAGTTTTTTTTGTTTTTTTTTTGTTAGTTTTATTGAAACCTTTTTATTTAGGAGATCCTGAAATGTTTGTTGAGGCTGACCCCATGATGAGTCCAGTGCATATTGTCCCAGAATGGTATTTTTTGTTTGCTTATGCTATTTTGCGGGCTATTCCTAATAAGGTTATGGGTGTTGTAGCTCTGTTGATAAGAATTTTGTTATTTTATTTTTTTTTACTGGTTAGTAATTATACGTCTTGTTTGG--

>KP721456

-GTTGAAATTATGGTAGAATGTTGGGAATAATTTTGGTTTTTCAATTGTTAACTGGTTTATTTTTATCTTTTTATTATGTTGCGGATGGTTTAGTAGCTTTTAGTTCGGTTCAATATATTATATCTGATGTTAATTTGGGTTGGTTGTTTCGGATTTTTCATTTTAATGGGGCAAGTTTGTTTTTTATTTTTTTGTATTTGCATATTTTTAAAGGTTTGTTTATGGTTAGTTATCGGTTAAAAAAAGTTTGAGGTACTGGTTTATTAATTTTTTTGTTAGTTATGATGGAGGCTTTTATAGGTTATGTTTTAGTTTGAGCTCAAATAAGATTTTGAGCTGCGGTTGTAATTACTAGGTTGTTGAGTGTCGTTCCTGTTTGAGGTCAGTTGATTGTGATGTGAATTTGAAGAGGTTTTGGGGTAACAAGGTCAACTTTAAAATTTTTTTTT---GTTTTACATTTTTTATTACCTTGGTTATTGTTGGTTTTGGTTGTAGTGCATATGATTTTTTTACATAGTACTGGTAGAACTTCTAGTTTGTATTGTCATGGTGATTATGATAAAATTAGTTTTGGTCCTTATTATTGGAATAAAGACTTTTATAATTTATTAGTTTTTTTTGTTTTTTTTTTGTTAGTTTTGTTGAAACCTTTTTATTTGGGAGATCCTGAAATGTTTGTTGAGGCAGATCCTATGATGAGACCGGTACATATTGTTCCAGAGTGATATTTTTTGTTTGCTTATGCTATTTTGCGGGCTATTCCTAATAAGGTTATGGGTGTTGTAGCTTTGTTGATAAGAATTTTGTTATTTTATTTTTTTTTATTGGTTAGTAATTATACATCTTGTTTGG--

>Aghazadeh A. mackerasae

-GTTGGAATTATGGTAGAATGTTGGGAATAATTTTAGTTTTTCAGTTGTTAACTGGATTATTTTTATCTTTTTATTATGTTGCGGATGGTTTAGTGGCTTTTAGTTCGGTTCAATATGTTATATTTGATGTTAATTTGGGTTGGTTATTTCGGATTTTTCATTTTAATGGGGCGAGTTTGTTTTTTATTTTTTTGTATTTGCATATTTTTAAGGGTTTGTTTATGGTTAGTTATCGGTTGAAAAAAGTTTGAGGTACGGGTTTATTGATTTTTTTGTTGATTATGATAGAGGCTTTTATGGGTTATGTTTTGGTCTGGGCTCAAATAAGATTTTGAGCTGCGGTTGTAATTACTAGGTTGTTGAGTGTTGTTCCTGTTTGAGGTCAATTGATTGTAATGTGAATTTGAAGAGGTTTTGGAGTAACCAGGTCAACTTTAAAGTTTTTTTTT---GTTTTACATTTTTTGTTGCCTTGGCTATTGTTGGTTTTGGTTATGGTGCATATGATTTTTTTGCATAGTACTGGTAGAACTTCTAGTTTGTATTGTCATGGTGATTATGATAAAATTAGTTTTGGTCCTTATTATTGGAATAAGGATTTTTATAATTTTTTAGTTTTTTTTGTTTTTTTTTTGTTAGTTTTATTGAAGCCTTTTTATTTAGGGGATCCCGAAATGTTTGTTGAGGCAGATCCCATGATGAGCCCAGTGCATATTGTTCCAGAATGGTATTTTTTGTTTGCTTATGCTATTTTGCGGGCTATTCCTAATAAGGTTATGGGTGTTGTAGCTTTGTTGATAAGAATTTTGTTATTTTATTTTTTTTTATTGGTTAGTAATTATACGTCTTGTTTGG--
