# Supplementary material for: Hurdles in the evolutionary epidemiology of Angiostrongylus cantonensis: Pseudogenes, incongruence between taxonomy and DNA sequence variants, and cryptic lineages
Source: Evol Appl. 2018 Mar 25;11(8):1257–69. doi: 10.1111/eva.12621 (PMC6099809; doi:10.1111/eva.12621)
Supplement: Supplementary file 4 [file EVA-11-1257-s004.docx]

>NC_013067

---------------------------------------ATTAATTTATATAAAAAATATCAAGGGGGTTTGTCGATTTGATTGGAGAGTTCTAATCATAAGGATATTGGTACGCTTTATTTTTTGTTTGGTTTGTGATCTGGGATGTTAGGGACTGCTTTTTCTTTGATTATTCGTTTAGAGTTGTCTAAGCCAGGGTTGTTATTATGTGGCGGACAGTTGTATAATTCGATTATTACTGCTCATGCTTTTTTAATAATTTTTTTTATAGTGATACCTAGGATGATTGGTGGTTTTGGTAATTGGATATTGCCTATAATGTTGGGGGCTCCTGATATGAGTTTTCCTCGTTTAAATAATCTGAGTTTTTGGTTGTTGCCAACTTCAATATTTTTAATTTTAGATTCTTGTTTTGTTGATATAGGTTGTGGTACGAGTTGAACTGTTTATCCTCCTTTAAGGACTTTAGGTCATCCAGGTAGGAGGGTTGATTTAGCTATTTTTAGTTTACATTGTGCTGGTCTAAGTTCTATTTTGGGTGGAATTAACTTTATATGTACGACTAAAAATATACGTAGTAGTTCTATTTCTTTGGAGCATATGAGTTTGTTTGTTTGGGCTGTGTTTGTGACTGTTTTTTTGTTGATTTTGTCTTTACCGGTTTTAGCTGGTGCTATTACTATGTTATTGACTGATCGTAATTTGAATACTTCTTTTTTTGATCCGAGTTCGGGAGGAAATCCTTTGATTTATCAACATTTGTTTTGGTTTTTTGGTCATCCTGAAGTATATATTTTGATTTTGCCAGCTTTTGGGATTATTAGTCAATCTGCTTTGTATTTGTCAGGGAAGAAAGAGGTTTTTGGTTATTTAGGGATGGTTTATGCGATTTTAAGAATTGGGTTGATTGGGTGTGTAGTTTGAGCTCATCATATGTATACTGTTGGTATGGATTTGGATTCTCGTGCTTACTTTACTGCAGCTACAATAGTTATTGCGGTTCCTACTGGGGTTAAAGTGTTTAGTTGGTTGGCTACACTTTATGGGATGAAAATGATGTTTCAGCCGATTTTGTTGTGGGTTATGGGGTTTATT---TTTTTGTTTACTATTGGGGGTTTGACCGGGGTTATGTTATCTAATTCAAGTTTGGATATTATTTTGCATGATACTTATTATGTGGTTAGGCATTTTCATTATGTATTAAGGTTGGGTGCAGTTTTTGGGATTTTTACTGGAGTTAGTCTTTGGTGGAGATTTATAAGAGGTTATGTTTATAATAAATTGTATATGGTAGTAGTGTTCTTTTTAATGTTTGTTGGTGTTAATTTAACTTTTTTTCCTTTACATTTTGCGGGTTTACACGGTTATCCTCGTAAGTATTTAGATTATCCTGATGTGTATTCTGTTTGGAATGTAATTTCTTCTTTTGGTTCTTTGGTTAGTGTGTTTGCTTTATTTATATTTGTTTTTTTATTATTAGAGTCTTTTTTTAGTTGTCGTTTAGTTTTAATGGATAACTATTATAATAGGAGTCCTGAATATAGATATAGGAATTATGTGTTTGGTCATAGGTATCAGTCCGAGATTTTTTTTAGAAGGAGAAGGTTGAAATATTAA

>AP017675 A. costaricensis

------------------------------ATCTCTTATATTAATTTATATAAGAAATATCAAGGGGGTTTGTTAATTTGGTTGGAGAGTTCTAACCATAAGGATATTGGAACGCTTTATTTTTTGTTTGGATTGTGATCTGGTATATTAGGAACTGCTTTTTCTTTGGTTATTCGTTTGGAGTTGTCTAAACCAGGTTTGTTATTGTGTGATGGACAGTTATATAATTCTATTATTACTGCTCATGCTTTTTTAATGATTTTCTTTATAGTAATACCTAGGATGATTGGAGGATTTGGTAATTGGATGTTACCTTTGATATTAGGGGCTCCAGATATGAGTTTTCCTCGTTTAAATAATTTAAGTTTTTGGTTATTGCCAGCTTCTATGTTTTTGATTTTGGATTCATGTTTTGTAGATATGGGTTGTGGAACTAGTTGAACTGTTTATCCGCCTTTAAGGACTTTAGGTCATGTGGGAAGAAGGGTTGATTTGGCTATTTTTAGTTTGCATTGTGCTGGTTTGAGTTCTATTTTGGGTGGAATTAATTTTATATGTACAACTAAGAATATGCGTAGTAGTTCGGTTTCTTTAGAACATATGAGTTTATTTGTTTGAGCTGTTTTTGTGACTGTTTTTTTATTGGTCTTGTCTTTGCCTGTTTTAGCTGGGGCTATTACTATATTGTTAGCTGATCGTAATTTAAATACTTCTTTTTTTGATCCTAGTTCGGGGGGTAATCCTTTAATTTATCAGCATTTATTTTGATTTTTTGGTCATCCTGAGGTCTATATTTTAATTTTGCCTGCTTTTGGTATTATTAGTCAATCTGCTTTGTATTTAACGGGAAAGAAAGAGGTTTTTGGTTATTTAGGGATGGTTTATGCAATTTTGAGAATTGGTTTAATTGGATGTGTGGTTTGAGCTCATCATATGTATACAGTAGGTATGGATTTGGATTCTCGTGCTTATTTTACTGCTGCTACTATGGTTATTGCTGTTCCTACGGGTGTTAAAGTTTTTAGTTGATTGGCTACTTTGTTTGGGATGAAGATGGTTTTTCAACCGGTTTTATTATGAGTTATGGGTTTTATT---TTTTTATTTACTATTGGGGGTTTAACAGGGGTAATATTATCTAATTCAAGTTTGGATATTATTTTACATGATACTTATTATGTAGTTAGACATTTTCATTATGTATTGAGATTGGGGGCAGTTTTTGGTATTTTTACAGGTATTAGTTTGTGATGAAGTTTTATAAGAGGTTGTGTGTATAATAAATTGTTTATGGTAGTGGTGTTTATTTTGATGTTTGTAGGTGTAAATTTAACTTTTTTTCCTTTACATTTTGCTGGTTTGCATGGTTATCCTCGAAAATATTTGGATTATCCTGATATTTATTCAGTTTGAAATGTAGTTTCTTCTTTTGGTTCTTTGTTAAGTGTTTTTGCTTTATTTATGTTTATTTTTTTGTTATTAGAGTCTTTTTTTAGTTATCGTTTGGTTTTGATAGATAATTATTATAATAGGAGTCCTGAGTATAGATATAGAAGTTATGTATTTGGTCATAGTTATCAATCAGAGATTTATTTTAGAAGAAGAAATTTGAAGTATTAA

>KR827449 A. costaricensis

---------------------------------------ATTAATTTATATAAGAAATATCAAGGGGGTTTGTTAATTTGGTTGGAGAGTTCTAACCATAAGGATATTGGAACGCTTTATTTTTTGTTTGGATTGTGATCTGGTATATTAGGAACTGCTTTTTCTTTGGTTATTCGTTTGGAGTTGTCTAAACCAGGTTTGTTATTGTGTGATGGACAGTTATATAATTCTATTATTACTGCTCATGCTTTTTTAATGATTTTCTTTATAGTAATACCTAGGATGATTGGAGGATTTGGTAATTGGATGTTACCTTTGATATTAGGGGCTCCAGATATGAGTTTTCCTCGTTTAAATAATTTAAGTTTTTGGTTATTGCCAGCTTCTATGTTTTTGATTTTGGATTCATGTTTTGTAGATATGGGTTGTGGAACTAGTTGAACTGTTTATCCGCCTTTAAGGACTTTAGGTCATGTGGGAAGAAGGGTTGATTTGGCTATTTTTAGTTTGCATTGTGCTGGTTTGAGTTCTATTTTGGGTGGAATTAATTTTATATGTACAACTAAGAATATGCGTAGTAGTTCGGTTTCTTTAGAACATATGAGTTTATTTGTTTGAGCTGTTTTTGTGACTGTTTTTTTATTGGTCTTGTCTTTGCCTGTTTTAGCTGGGGCTATTACTATATTGTTAGCTGATCGTAATTTAAATACTTCTTTTTTTGATCCTAGTTCGGGGGGTAATCCTTTAATTTATCAGCATTTATTTTGATTTTTTGGTCATCCTGAGGTCTATATTTTAATTTTGCCTGCTTTTGGTATTATTAGTCAATCTGCTTTGTATTTAACGGGAAAGAAAGAGGTTTTTGGTTATTTAGGGATGGTTTATGCAATTTTGAGAATTGGTTTAATTGGATGTGTGGTTTGAGCTCATCATATGTATACAGTAGGTATGGATTTGGATTCTCGTGCTTATTTTACTGCTGCTACTATGGTTATTGCTGTTCCTACGGGTGTTAAAGTTTTTAGTTGATTGGCTACTTTGTTTGGGATGAAGATGGTTTTTCAACCGGTTTTATTATGAGTTATGGGTTTTATT---TTTTTATTTACTATTGGGGGTTTAACAGGGGTGATATTATCTAATTCAAGTTTGGATATTATTTTACATGATACTTATTATGTAGTTAGACATTTTCATTATGTATTGAGATTGGGGGCAGTTTTTGGTATTTTTACAGGTATTAGTTTGTGATGAAGTTTTATAAGAGGTTGTGTGTATAATAAATTGTTTATGGTAGTGGTGTTTATTTTGATGTTTGTAGGTGTAAATTTAACTTTTTTTCCTTTACATTTTGCTGGTTTGCATGGTTATCCTCGAAAATATTTGGATTATCCTGATATTTATTCAGTTTGAAATGTAGTTTCTTCTTTTGGTTCTTTGTTAAGTGTTTTTGCTTTATTTATGTTTATTTTTTTGTTATTAGAGTCTTTTTTTAGTTATCGTTTGGTTTTGATAGATAATTATTATAATAGGAGTCCTGAGTATAGATATAGAAGTTATGTATTTGGTCATAGTTATCAATCAGAGATTTATTTTAGAAGAAGAAATTTGAAGTATTAA

>NC_018602 A. vasorum

---------------------------------------------ATATATAAGAAATATCAGGGGGGTTTATTAGTTTGGTTGGAGAGTTCTAATCATAAGGATATTGGTACGTTGTATTTTTTGTTTGGTTTGTGATCTGGTATATTGGGTGCTGCTTTTTCTTTAATTATTCGTTTGGAATTGTCTAAGCCTGGATTGTTGTTGTGTAGGGGTCAGTTGTATAATTCTATTATTACGGCTCATGCTTTTTTGATGATTTTTTTTATGGTTATGCCTAGGATGATTGGGGGTTTTGGTAATTGGATGTTGCCTTTGATGTTGGGAGCGCCGGATATAAGTTTTCCTCGTTTGAATAATTTGAGTTTTTGGTTGTTGCCTACTTCAATGTTTTTGATTTTAGATTCTTGTTTTGTGGATATGGGTTGTGGTACGAGGTGAACTGTGTATCCTCCGTTGAGTAGTTTGGGTCATCCGGGGAGAAGGGTGGATTTGGCTATTTTTAGTTTGCACTGTGCGGGTTTGAGTTCTATTTTAGGTGGGATTAATTTTATGTGCACTACTAAGAATATGCGGAGTAGATCTATTTCTTTGGAGCATATAAGGTTGTTTGTTTGAGCGGTTTTTGTGACTGTTTTTTTGTTAGTTTTGTCTTTGCCTGTTTTAGCTGGAGCGATTACTATGTTATTGACGGATCGTAATTTGAATACTTCTTTTTTTGATCCTAGTTCTGGGGGGAATCCTTTGATTTATCAGCATTTGTTTTGGTTTTTTGGTCATCCGGAGGTTTATGTTTTGATTTTGCCGGCTTTTGGGATTGTTAGTCAGTCGACTTTATATTTGACTGGGAAGAAGGAGGTGTTTGGTTATTTGGGGATGGTTTATGCGATTTTAAGGATTGGTTTGATTGGTTGTGTGGTGTGAGCTCATCATATGTATACTGTTGGTATAGATTTAGATTCTCGTGCTTATTTTACTGCGGCTACTATGGTGATTGCGGTGCCGACTGGAGTGAAGGTTTTTAGTTGGTTGGCTACTTTGTTTGGGATGAAGTATAGTATTTCAGCCTATTTTGTTGTGGGTTATAGGATTTATTTTTTTTTATTTACTATTGGGGGTTTGACGGGTGTGATATTGTCAAATTCGAGATTGGATATTATTTTACATGATACGTATTATGTGGTAAGTCATTTTCATTATGTGTTGAGGCTGGGGGCTGTTTTTGGGATTTTTACGGGGGTTAGTTTATGGTGAAGATTTATGACGGGTTATGTTTATAATAAGTTGTATATGGTGGTGATGTTTTTTTTAATGTTTGTGGGTGTGAATTTAACTTTTTTTCCTTTGCATTTTGCTGGTTTGCACGGTTATCCTCGTAAATATTTGGATTATCCCGATGCTTATTCAGTTTGAAATGTAGTATCTTCTTTTGGTTCTTTGGTGAGAGTGTTTGCTTTGTTTATGTTTATTTTTTTGTTGTTGGAGTCTTTTTTTAGTTATCGTTTGGTGTTGGTGGATAATTATTATAATAGGAGTCCAGAGTATAGGTATAGGAGTTATGTGTTTGGTCATAGGTATCAGTCGGATGTTTATTTTAGTAGAAGAAGTTTAAAGTGTTAA

>KT947978 A. cantonensis

---------------------------------------ATTTATATATATAAGAAATATCAAAGTGGTTTGTCGGTTTGGTTAGAAAGTTCTAACCATAAGGATATTGGTACTCTTTATTTTTTGTTTGGTTTGTGGTCGGGTATGCTAGGTACTGCTTTATCTTTGATTGTTCGTTTGGAGTTATCTAAGCCTGGAATGTTGTTGTCTAATGGGCAATTATATAACTCAATTATTACGGCTCATGCTTTTTTGATAATTTTTTTTATGGTAATGCCAAGTATGATTGGTGGTTTTGGTAATTGAATGTTGCCTTTGATATTGGGGGCTCCAGATATGAGTTTTCCTCGTTTGAATAATTTGAGTTTTTGATTGTTGCCAACTTCGATGTTTTTGATTTTAGATTCTTGTTTTGTAGATATAGGATGTGGGACTAGTTGGACTGTTTATCCACCTTTGAGGAGTTTAGGTCATCCTGGTAGAAGGGTAGATTTGGCGATTTTTAGTTTGCATTGTGCTGGATTGAGTTCTATTTTGGGTGGTATTAATTTTATGTGTACGACGAAGAATATGCGGAGAAGTTCAATTTCTTTGGAACATATAAGTTTGTTTGTTTGATCTGTTTTTGTGACTGTTTTTTTGTTGGTATTGTCTTTACCTGTGTTGGCGGGGGCTATTACTATATTATTGACTGATCGTAATTTGAATACTTCTTTTTTTGATCCTAGTTCTGGTGGTAATCCTTTGATTTATCAGCATTTGTTTTGATTTTTTGGTCATCCTGAGGTTTATGTTTTGATTTTGCCTGCTTTTGGGATTGTTAGACAGTCTACTTTATATTTAACGGGTAAGAAGGAGGTTTTTGGTTATTTGGGTATGGTTTATGCTATTTTAAGGATTGGTTTGATTGGTTGTGTGGTTTGGGCTCATCATATATATACGGTTGGTATGGATTTAGATTCTCGTGCTTATTTTACTGCTGCTACTATAGTTATTGCGGTTCCTACGGGAGTGAAGGTTTTTAGGTGGTTAGCAACTTTATTTGGTATAAAGATATTGTTTCAACCTATTTTATTGTGGGTTTTGGGTTTTATT---TTTTTATTTACTATTGGGGGGTTAACTGGGGTTATATTGTCTAATTCTAGTTTGGATATTATTTTACATGATACTTATTATGTAGTTAGGCATTTTCATTATGTTTTAAGGTTGGGGGCTGTTTTTGGTATTTTTACTGGTATTAGTCTTTGATGAAGGTTTATGACTGGTTGTGTTTATAATAAGTTGTATATAGTGGTTATGTTTTTTTTGATGTTTTTAGGTGTGAATTTGACTTTTTTTCCTTTACATTTTGCTGGTTTGCATGGTTATCCTCGTAAGTATTTGGATTATCCTGATGTTTATTCGGTTTGAAATGTTATTTCTTCTTTTGGGTCTTTAGTTAGTGTTTTTGCTTTATTTATATTTATTTTTTTGTTGTTGGAGTCTTTTTTTAGATATCGTTTGTTGTTATTGGATAATTATTATAATAGAAGTCCAGAATATAGGTATAGGAGTTATGTTTTTGGCCATAGCTATCAGTCGGAAGTTTATTTTAGAAGAAGGAGTTTAAAGTGTTAA

>NC_013065 A. cantonensis

---------------------------------------ATTTATATATATAAGAAATATCAAGGTGGTTTGTCGGTTTGATTAGAAAGTTCTAATCATAAGGATATTGGTACTCTTTATTTTTTGTTTGGTTTATGGTCGGGTATGTTAGGTACTGCTTTATCTTTGATTGTTCGTTTGGAATTGTCTAAGCCTGGAATGTTGTTGTCTAATGGGCAATTGTATAATTCAATTATTACGGCTCATGCTTTTTTGATGATTTTTTTTATGGTGATGCCTAGTATGATTGGTGGTTTTGGTAATTGAATATTGCCTTTGATGTTGGGGGCTCCGGATATGAGTTTTCCTCGTTTGAATAATTTGAGTTTTTGATTATTACCAACTTCGATGTTTTTGATTTTAGATTCTTGTTTTGTGGATATAGGATGTGGGACTAGTTGGACTGTTTATCCGCCTTTGAGAAGTTTAGGTCATCCTGGTAGAAGGGTGGATTTGGCGATTTTTAGTTTGCATTGTGCTGGACTGAGTTCTATTTTGGGGGGTATTAATTTTATGTGTACAACGAAGAATATGCGAAGAAGTTCAATTTCTTTGGAACATATGAGTTTGTTTGTTTGATCTGTTTTTGTGACTGTTTTTTTGTTGGTGTTGTCTTTACCTGTGTTGGCCGGGGCTATCACTATATTATTAACTGATCGTAATTTAAATACTTCTTTTTTTGATCCTAGTTCTGGTGGTAATCCTTTGATTTATCGGCATTTGTTTTGATTTTTTGGTCATCCTGAGGTTTATGTTTTGATTTTGCCTGCTTTTGGGATTGTTAGACAGTCTACTTTATATTTAACGGGTAAAAAAGAGGTTTTTGGTTATTTGGGTATGGTTTATGCTATTTTAAGAATTGGTTTGATTGGTTGTGTGGTTTGGGCTCATCATATATATACGGTTGGTATGGATTTAGATTCTCGTGCTTATTTTACTGCGGCTACTATAGTTATTGCGGTTCCTACGGGAGTGAAGGTTTTTAGGTGGTTGGCAACTTTATTTGGTATAAAGATATTGTTTCAACCTATTTTATTGTGGGTTTTGGGTTTTATT---TTTTTATTTACTATCGGTGGGTTAACGGGGGTTATATTGTCTAATTCTAGTTTGGATATTATTTTACATGATACTTATTATGTAGTTAGGCATTTTCATTATGTTTTAAGGTTGGGGGCTGTTTTTGGTATTTTTACTGGTATTAGTCTTTGATGAGGGTTTATGACTGGTTGTGTTTATAATAAGTTGTATATAGTGGTGATGTTTTTTTTGATGTTTTTGGGTGTGAATTTGACTTTTTTTCCTTTGCATTTTGCTGGTTTACATGGTTATCCTCGTAAGTATTTAGATTATCCTGATGTTTATTCAGTTTGAAATGTTATTTCTTCTTTTGGGTCTTTAATTAGTGTTTTTGCTTTATTTATATTTATTTTTTTGTTGTTGGAGTCTTTTTTTAGATATCGTTTGTTGTTACAGGATAATTATTATAATAGAAGTCCTGAGTATAGATATAGAAGTTATGTTTTTGGTCATAGTTATCAGTCAGAAGTTTATTTTAGAAGGAGGAGTTTAAAGCGTTAG

>AP017672 A. cantonensis

ATAAGAGTGAAATTTTGATTGGAAGAAAAATTCTCTTACATTTATATATATAAGAAATATCAAAGTGGTTTGTCGGTTTGGTTAGAAAGTTCTAACCATAAGGATATTGGTACTCTTTATTTTTTGTTTGGTTTGTGGTCGGGTATGTTAGGTACTGCTTTATCTTTGATTGTTCGTTTGGAGTTATCTAAGCCTGGAATGTTGTTGTCTAATGGGCAATTGTATAATTCAATTATTACGGCTCATGCTTTTTTGATAATTTTTTTTATGGTAATGCCTAGTATGATTGGTGGTTTTGGTAATTGAATGTTGCCTTTGATGTTGGGGGCTCCAGATATGAGTTTTCCTCGTTTGAATAATTTGAGTTTTTGATTGTTGCCAACTTCGATGTTTTTGATTTTAGATTCTTGTTTTGTAGATATAGGATGTGGAACTAGTTGGACTGTTTATCCACCTTTGAGGAGTTTAGGTCATCCTGGTAGAAGGGTAGATTTGGCGATTTTTAGTTTGCATTGTGCTGGATTGAGTTCTATTTTGGGGGGTATTAATTTTATGTGTACGACGAAGAATATACGAAGAAGTTCAATTTCTTTGGAACATATAAGTTTGTTTGTTTGATCTGTTTTTGTAACTGTTTTTTTGTTGGTATTGTCTTTACCTGTGTTGGCGGGGGCTATTACCATGTTATTAACTGATCGTAATTTAAATACTTCTTTTTTTGATCCTAGTTCTGGTGGTAATCCTTTGATTTATCAGCATTTGTTTTGATTTTTTGGCCATCCTGAGGTTTATGTTTTGATTTTGCCTGCTTTTGGGATTGTTAGACAGTCTACTTTATATTTAACGGGTAAGAAGGAGGTTTTTGGTTATTTGGGTATGGTTTATGCTATTTTAAGGATTGGTTTGATTGGTTGTGTGGTTTGGGCTCATCATATATACACGGTTGGTATGGATTTAGATTCTCGTGCTTATTTTACTGCAGCTACTATAGTTATTGCGGTTCCTACGGGAGTGAAGGTTTTTAGGTGGTTAGCAACTTTATTTGGTATGAAGATATTGTTTCAACCTATTTTATTGTGGGTTTTGGGTTTTATT---TTTTTGTTTACTATTGGGGGGTTGACTGGGGTTATATTGTCTAATTCTAGTTTGGATATTATTTTACATGATACTTATTATGTAGTTAGTCATTTTCATTATGTTTTAAGGTTGGGAGCTGTTTTTGGTATTTTTACTGGTATTAGTCTTTGATGAAGGTTTATGACTGGTTGTGTTTATAATAAGTTGTATATAGTAGTTATGTTTTTTTTGATGTTTTTAGGTGTGAATTTGACTTTTTTTCCTTTGCATTTTGCTGGTTTGCACGGTTATCCTCGTAAGTATTTGGATTATCCTGATGTTTATTCGGTTTGAAATGTTATTTCTTCTTTTGGGTCTTTAGTTAGTGTTTTTGCTTTATTTATGTTTATTTTTTTGTTGTTGGAGTCTTTTTTTAGATATCGTTTGTTGTTATTGGATAATTATTATAATAGAAGTCCAGAATATAGATATAGGAGTTATGTTTTTGGTCATAGCTATCAGTCGGAAATTTATTTTAGAAGAAGGAGTTTAAAGTGTTAA

>KT947979 A. malaysiensis

---------------------------------------ATTAATATATATAAAAAATATCAAGGTGGTTTGTTAGTTTGATTAGAGAGTTCTAATCATAAGGATATTGGTACTCTTTATTTTTTGTTTGGATTTTGGTCGGGTATGTTAGGTACTGCATTATCTTTGATTATTCGTTTGGAATTATCTAAACCAGGGATGTTGTTATCTAATGGGCAATTGTATAATTCGATTATTACGGCGCATGCTTTTTTGATGATTTTTTTTATAGTAATACCAAGTATGATTGGAGGATTTGGTAATTGAATGTTGCCTTTAATGTTGGGGGCTCCTGATATGAGTTTTCCTCGTTTAAATAATTTGAGTTTTTGATTATTGCCAACTTCGATGTTTTTGATTTTGGATTCTTGTTTTGTTGATATAGGTTGTGGTACTAGATGAACTGTTTATCCGCCTTTGAGAAGTTTGGGTCATCCTGGTAGAAGAGTGGATTTAGCGATTTTTAGTTTACATTGTGCTGGATTGAGTTCTATTTTGGGAGGTATTAATTTTATATGTACAACTAAGAATATACGAAGGAGTTCAATTTCTTTGGAGCATATGAGTTTATTTGTTTGATCTATTTTTGTAACTGTTTTTTTGTTGGTGTTGTCTTTACCTGTATTAGCAGGGGCTATTACTATATTATTAACGGATCGTAATTTGAATACTTCTTTTTTTGATCCTAGTTCTGGAGGTAATCCTTTAATTTATCAACACTTATTTTGATTTTTTGGTCATCCAGAGGTTTATGTCTTGATTTTGCCTGCATTTGGAATTGTTAGGCAATCTACTTTGTATTTAACGGGTAAAAAAGAAGTTTTTGGTTATTTAGGTATAGTTTATGCTATTTTAAGTATTGGTTTAATTGGTTGTGTAGTGTGGGCTCATCATATATATACAGTAGGTATAGATTTAGATTCTCGTGCTTATTTTACTGCGGCTACGATGGTTATTGCTGTTCCTACAGGGGTAAAGGTGTTTAGATGATTAGCAACTTTGTTTGGTATAAAAATGTTATTTCAGCCTATTTTGTTGTGGGTTTTAGGTTTCATT---TTTTTATTTACTATTGGGGGTTTGACTGGGGTTATATTGTCTAATTCTAGTTTAGATATTATTTTACATGATACTTATTATGTAGTTAGGCATTTTCATTATGTTTTGAGGTTAGGGGCTGTTTTTGGTATTTTTACTGGTATTAGTCTTTGATGAAGTTTTATGACTGGTTACGTATATAATAAGTTATATATAGTAGTGATATTTTTTTTGATGTTTTTGGGTGTGAATTTGACTTTTTTTCCTTTGCATTTTGCTGGTTTGCACGGGTATCCACGTAAGTATTTAGATTATCCTGATGTTTATTCGGTTTGAAATGTTATTTCTTCTTTTGGGTCTTTAGTTAGTGTTTTTGCTTTGTTTATATTTATTTTTTTGTTATTAGAATCTTTTTTTAGGTATCGCTTGGTTTTAATGGATAATTATTATAATAGAAGTCCTGAGTATAGATATAGGAGTTATGTTTTTGGTCATAGTTATCAGTCAGAAGTTTATTTTAGAAGAAGAATTTTGAAATATTAA

>KT186242 A. cantonensis

---------------------------------------ATTAATATATATAAAAAATATCAAGGTGGTTTGTTAGTTTGATTAGAGAGTTCTAATCATAAGGATATTGGTACTCTTTATTTTTTGTTTGGATTTTGGTCGGGTATGTTAGGTACTGCATTATCTTTGATTATTCGTTTGGAATTATCTAAACCAGGGATGTTGTTATCTAATGGGCAATTGTATAATTCGATTATTACGGCGCATGCTTTTTTGATGATTTTTTTTATAGTAATACCAAGTATGATTGGAGGATTTGGTAATTGAATGTTGCCTTTAATGTTGGGGGCTCCTGATATGAGTTTTCCTCGTTTAAATAATTTGAGTTTTTGATTATTGCCAACTTCGATGTTTTTGATTTTGGATTCTTGTTTTGTTGATATAGGTTGTGGTACTAGATGAACTGTTTATCCGCCTTTGAGAAGTTTAGGTCATCCTGGTAGAAGAGTGGATTTAGCGATTTTTAGTTTACATTGTGCTGGATTGAGTTCTATTTTGGGAGGTATTAATTTTATATGTACAACTAAGAATATACGAAGGAGTTCAATTTCTTTGGAGCATATGAGTTTATTTGTTTGATCTATTTTTGTAACTGTTTTTTTGTTGGTGTTGTCTTTACCTGTATTAGCAGGGGCTATTACTATATTATTAACGGATCGTAATTTGAATACTTCTTTTTTTGATCCTAGTTCTGGAGGTAATCCTTTAATTTATCAACACTTATTTTGATTTTTTGGTCATCCAGAGGTTTATGTCTTAATTTTGCCTGCATTTGGAATTGTTAGGCAATCTACTTTGTATTTAACGGGTAAAAAAGAAGTTTTTGGTTATTTAGGTATAGTTTATGCTATTTTAAGTATTGGTTTAATTGGTTGTGTAGTGTGGGCTCATCATATATATACAGTAGGTATAGATTTAGATTCTCGTGCTTATTTTACTGCGGCTACGATGGTTATTGCTGTTCCTACAGGGGTAAAGGTGTTTAGATGATTAGCAACTTTGTTTGGTATAAAAATGTTATTTCAGCCTATTTTGTTGTGGGTTTTAGGTTTCATT---TTTTTATTTACTATTGGGGGTTTGACTGGGGTTATATTGTCTAATTCTAGTTTAGATATTATTTTACATGATACTTATTATGTAGTTAGGCATTTTCATTATGTTTTGAGGTTAGGGGCTGTTTTTGGTATTTTTACTGGTATTAGTCTTTGATGAAGTTTTATGACTGGTTACGTATATAATAAGTTATATATAGTAGTGATATTTTTTTTGATGTTTTTGGGTGTGAATTTGACTTTTTTTCCTTTGCATTTTGCTGGTTTGCACGGGTATCCACGTAAGTATTTAGATTATCCTGATGTTTATTCGGTTTGAAATGTTATTTCTTCTTTTGGGTCTTTAGTTAGTGTTTTTGCTTTGTTTATATTTATTTTTTTGTTATTAGAATCTTTTTTTAGGTATCGCTTGGTTTTAATGGATAATTATTATAATAGAAGTCCTGAGTATAGATATAGGAGTTATGTTTTTGGTCATAGTTATCAGTCAGAAGTTTATTTTAGAAGAAGAATTTTGAAATATTAA

>AB684358 ac1

------------------------------------------------------------------------------------------------------------------------------------------------------------------------------------------------------------------------------------------------------------------------------------------------------------------------------------------------------------------------------------------------------------------------------------------------------------------------------------------------------------------------------------------------------------------------------------------------------------------------------------------------------------------------------------------------------------------------------------------------------------------------------------------------------------------------------------------TTTGGGATTGTTAGACAGTCTACTTTATATTTAACGGGTAAAAAAGAGGTTTTTGGTTATTTGGGTATGGTTTATGCTATTTTAAGAATTGGTTTGATTGGTTGTGTGGTTTGGGCTCATCATATATATACGGTTGGTATGGATTTGGATTCTCGTGCTTATTTTACTGCGGCTACTATAGTTATTGCGGTTCCTACGGGAGTGAAGGTTTTTAGGTGGTTGGCAACTTTATTTGGTATAAAGATATTGTTTCAACCTATTTTATTGTGGGTTTTGGGTTTTATT---TTTTTATTTACTATTGGTGGGTTAACCGGGGTTATATTGTCTAATTCTAGTTTGGATATTATTTTACATGATACTTATTATGTAGTTAGGCATTTTCATTATGTTTTAAGGTTGGGGGCTGTTTTTGGTATTTTTACTGGTATTAGTCTTTGATGAGGGTTTATGACTGGTTGTGTTTATAATAAGTTGTATATAGTGGTGATGTTTTTTTTGATGTTTTTGGGTGTGAATTTGACTTTTTTTCCTTTGCATTTTGCTGGTTTACATGGTTATCCTCGTA--------------------------------------------------------------------------------------------------------------------------------------------------------------------------------------------------------------------------------------------------

>AB684367 ac3

------------------------------------------------------------------------------------------------------------------------------------------------------------------------------------------------------------------------------------------------------------------------------------------------------------------------------------------------------------------------------------------------------------------------------------------------------------------------------------------------------------------------------------------------------------------------------------------------------------------------------------------------------------------------------------------------------------------------------------------------------------------------------------------------------------------------------------------TTTGGGATTGTTAGACAGTCTACTTTATATTTAACGGGTAAAAAGGAGGTTTTTGGTTATTTGGGTATGGTTTATGCTATTTTAAGAATTGGTTTGATTGGTTGTGTGGTTTGGGCTCATCATATATATACGGTTGGTATGGATTTAGATTCTCGTGCTTATTTTACTGCGGCTACTATAGTTATTGCGGTTCCTACGGGAGTGAAGGTTTTTAGGTGGTTGGCAACTTTATTTGGTATAAAGATATTGTTTCAACCTATTTTATTGTGGGTTTTGGGCTTTATT---TTTTTATTTACTATTGGTGGGTTAACTGGGGTTATATTGTCTAATTCTAGTTTGGATATTATTTTACATGATACTTATTATGTAGTTAGGCATTTTCATTATGTTTTAAGGTTGGGGGCTGTTTTTGGTATTTTTACTGGTATTAGTCTTTGATGAGGGTTTATGACCGGTTGTGTTTATAATAAGTTGTATATAGTGGTAATGTTTTTTTTGATGTTTTTGGGTGTGAATTTGACTTTTTTTCCTTTGCATTTTGCTGGTTTACATGGTTATCCTCGTA--------------------------------------------------------------------------------------------------------------------------------------------------------------------------------------------------------------------------------------------------

>AB684368 ac4

------------------------------------------------------------------------------------------------------------------------------------------------------------------------------------------------------------------------------------------------------------------------------------------------------------------------------------------------------------------------------------------------------------------------------------------------------------------------------------------------------------------------------------------------------------------------------------------------------------------------------------------------------------------------------------------------------------------------------------------------------------------------------------------------------------------------------------------TTTGGGATTGTTAGACAGTCTACTTTATATTTAACGGGTAAGAAGGAGGTTTTTGGTTATTTGGGTATGGTTTATGCTATTTTAAGGATTGGTTTGATTGGTTGTGTGGTTTGGGCTCATCATATATATACGGTTGGTATGGATTTAGATTCTCGTGCTTATTTTACTGCGGCTACTATAGTTATTGCGGTTCCTACGGGAGTGAAGGTTTTTAGGTGGTTAGCAACTTTATTTGGTATAAAGATATTGTTTCAACCTATTTTATTGTGGGTTTTGGGTTTTATT---TTTTTATTTACTATTGGGGGGTTAACTGGGGTTATATTGTCTAATTCTAGTTTGGATATTATTTTACATGATACTTATTATGTAGTTAGGCATTTTCATTATGTTTTAAGGTTGGGGGCTGTTTTTGGTATTTTTACTGGTATTAGTCTTTGATGAAGGTTTATGACTGGTTGTGTTTATAATAAGTTGTATATAGTGGTTATGTTTTTTTTGATGTTTTTAGGTGTGAATTTGACTTTTTTTCCTTTACATTTTGCTGGTTTGCATGGTTATCCTCGTA--------------------------------------------------------------------------------------------------------------------------------------------------------------------------------------------------------------------------------------------------

>AB684369 ac5

------------------------------------------------------------------------------------------------------------------------------------------------------------------------------------------------------------------------------------------------------------------------------------------------------------------------------------------------------------------------------------------------------------------------------------------------------------------------------------------------------------------------------------------------------------------------------------------------------------------------------------------------------------------------------------------------------------------------------------------------------------------------------------------------------------------------------------------TTTGGGATTGTTAGACAGTCTACTTTATATTTAACGGGTAAGAAGGAGGTTTTTGGTTATTTGGGTATGGTTTATGCTATTTTAAGGATTGGTTTGATTGGTTGTGTGGTTTGGGCTCACCATATATACACGGTTGGTATGGATTTAGATTCTCGTGCTTATTTTACTGCGGCTACTATAGTTATTGCGGTTCCTACGGGAGTGAAGGTTTTTAGGTGGTTAGCAACTTTATTTGGTATGAAGATATTGTTTCAACCTATTTTATTGTGGGTTTTGGGTTTTATT---TTTTTGTTTACTATTGGGGGGTTGACTGGAGTTATATTGTCTAATTCTAGTTTGGATATTATTTTACATGATACTTATTATGTAGTTAGGCATTTTCATTATGTTTTAAGGTTGGGGGCTGTTTTTGGTATTTTTACTGGTATTAGTCTTTGATGAAGGTTTATGACTGGTTGTGTTTATAATAAGTTGTATATAGTGGTTATGTTTTTTTTGATGTTTTTAGGTGTGAATTTGACTTTTTTTCCTTTGCATTTTGCTGGTTTGCATGGCTATCCTCGTA--------------------------------------------------------------------------------------------------------------------------------------------------------------------------------------------------------------------------------------------------

>KU532144 ac5b

------------------------------------------------------------------------------------------------------------------------------------------------------------------------------------------------------------------------------------------------------------------------------------------------------------------------------------------------------------------------------------------------------------------------------------------------------------------------------------------------------------------------------------------------------------------------------------------------------------------------------------------------------------------------------------------------------------------------------------------------------------------------------------------------------------------------GTTTTGATTTTGCCTGCTTTTGGGATTGTTAGACAGTCTACTTTATATTTAACGGGTAAGAAGGAGGTTTTTGGTTATTTGGGTATGGTTTATGCTATTTTAAGGATTGGTTTGATTGGTTGTGTGGTTTGGGCTCACCATATATACACGGTTGGTATGGATTTAGATTCTCGTGCTTATTTTACTGCGGCTACTATAGTTATTGCGGTTCCTACGGGAGTGAAGGTTTTTAGGTGGTTAGCAACTTTATTTGGTATGAAGATATTGTTTCAACCTATTTTATTGTGGGTTTTGGGTTTTATT---TTTTTGTTTACTATTGGGGGGTTGACTGGAGTTATATTGTCTAATTCTAGTTTGGATATTATTTTACATGATACTTATTATGTAGTTAGGCATTTTCATTATGTTTTAAGGTTGGGGGCTGTTTTTGGTATTTTTACTGGTATTAGTCTTTGATGAAGGTTTATGACTGGTTGTGTTTATAATAAGTTGTATATAGTGGTTATGTTTTTTTTGATGTTTTTAGGTGTGAATTTGACTTTTTTTCCTTTGC--------------------------------------------------------------------------------------------------------------------------------------------------------------------------------------------------------------------------------------------------------------------------------

>AB684374 ac6

------------------------------------------------------------------------------------------------------------------------------------------------------------------------------------------------------------------------------------------------------------------------------------------------------------------------------------------------------------------------------------------------------------------------------------------------------------------------------------------------------------------------------------------------------------------------------------------------------------------------------------------------------------------------------------------------------------------------------------------------------------------------------------------------------------------------------------------TTTGGGATTGTTAGACAGTCTACTTTATATTTGACTGGTAAGAAGGAGGTTTTTGGTTATTTGGGTATGGTTTATGCTATTTTGAGAATTGGTTTGATTGGTTGTGTGGTATGGGCTCATCATATATATACGGTTGGTATGGATTTAGATTCTCGTGCTTATTTTACTGCGGCTACTATGGTTATTGCGGTCCCTACGGGAGTAAAGGTTTTTAGGTGGTTAGCAACTTTATTTGGTATAAAAATGTTGTTTCAACCTATTTTATTGTGGGTTTTGGGTTTTATT---TTTTTATTTACTATTGGGGGATTAACTGGGGTTATATTGTCTAATTCTAGTTTGGATATTATTTTACATGATACTTATTATGTGGTTAGACATTTTCATTATGTTTTAAGATTGGGGGCTGTTTTTGGTATTTTTACAGGTATTAGTCTTTGATGGAGGTTTATGACTGGTTGTGTTTATAATAAGTTGTATATAGTGGTGGTGTTTTTTTTAATGTTTTTAGGTGTAAATTTGACTTTTTTTCCTTTGCATTTTGCTGGTTTACGTGGTTATCCTCGTA--------------------------------------------------------------------------------------------------------------------------------------------------------------------------------------------------------------------------------------------------

>AB684375 ac7

------------------------------------------------------------------------------------------------------------------------------------------------------------------------------------------------------------------------------------------------------------------------------------------------------------------------------------------------------------------------------------------------------------------------------------------------------------------------------------------------------------------------------------------------------------------------------------------------------------------------------------------------------------------------------------------------------------------------------------------------------------------------------------------------------------------------------------------TTTGGGATTGTTAGACAGTCTACTTTATATTTGACTGGTAAGAAGGAGGTTTTTGGTTATTTGGGTATGGTTTATGCTATTTTAAGAATTGGTTTGATTGGTTGTGTGGTATGGGCTCATCATATATATACGGTTGGTATAGATTTAGATTCTCGTGCTTATTTTACTGCGGCTACTATGGTTATTGCGGTTCCTACGGGAGTAAAGGTTTTTAGGTGGTTAGCAACTTTATTTGGTATAAAAATGTTGTTTCAACCTATTTTATTGTGGGTTTTGGGTTTTATT---TTTTTGTTTACTATTGGGGGATTAACTGGGGTTATATTGTCTAATTCTAGTTTGGATATTATTTTACATGATACTTATTATGTGGTTAGACATTTTCACTATGTTTTAAGATTGGGGGCTGTTTTTGGTATTTTTACAGGTATTAGTCTTTGATGGAGGTTTATGACTGGTTGTGTTTATAATAAGTTGTATATGGTGGTGGTGTTTTTTTTAATGTTTTTAGGTGTAAATTTGACTTTTTTTCCTTTGCATTTTGCTGGTTTACATGGTTATCCTCGTA--------------------------------------------------------------------------------------------------------------------------------------------------------------------------------------------------------------------------------------------------

>HQ440217 ac8

-----------------------------------------------------------------------------------------------------------------------------------------------------------------------------------------------------------------------------------------------------------------------------------------------------------------------------------------------------------------------------------------------------------------------------------------------------------------------------------------------------------------------------------------------------------------------------------------------------------------------------------------------------------------------------------------------------------------------------------------------------------------------------------------------------------------------------------------------------TAGACAGTCTACTTTATATTTAACGGGTAAGAAGGAGGTTTTTGGTTATTTGGGTATGGTTTATGCTATTTTAAGGATTGGTTTGATTGGTTGTGTGGTTTGGGCTCATCATATATACACGGTTGGTATGGATTTAGATTCTCGTGCTTATTTTACTGCAGCTACTATAGTTATTGCGGTTCCTACGGGAGTGAAGGTTTTTAGGTGGTTAGCAACTTTATTTGGTATGAAGATATTGTTTCAACCTATTTTATTGTGGGTTTTGGGTTTTATT---TTTTTGTTTACTATTGGGGGGTTGACTGGGGTTATGTTGTCTAATTCTAGTTTGGATATTATTTTACATGATACTTATTATGTAGTTAGTCATTTTCATTATG-----------------------------------------------------------------------------------------------------------------------------------------------------------------------------------------------------------------------------------------------------------------------------------------------------------------------------------------------------------------------------------------------------------------------------------

>JX471055 ac9

-----------------------------------------------------------------------------------------------------------------------------------------------------------------------------------------------------------------------------------------------------------------------------------------------------------------------------------------------------------------------------------------------------------------------------------------------------------------------------------------------------------------------------------------------------------------------------------------------------------------------------------------------------------------------------------------------------------------------------------------------------------------------------------------------------------------------------------------------------TAGACAGTCTACTTTATATTTGACTGGTAAGAAGGAGGTTTTTGGTTATTTGGGTATGGTTTATGCTATTTTGAGAATTGGTTTGATTGGTTGTGTGGTATGGGCTCATCATATATATACGGTTGGTATGGATTTAGATTCTCGTGCTTATTTTACTGCGGCTACTATGGTTATTGCGGTTCCTACTGGAGTAAAGGTTTTTAGGTGGTTAGCAACTTTATTTGGTATAAAAATGTTGTTTCAGCCTATTTTATTGTGGGTTTTGGGTTTTATT---TTTTTATTTACTATTGGTGGGTTAACTGGGGTTATGTTATCTAATTCTAGTTTGGATATTATTTTACATGATACTTATTATGTGGT----------------------------------------------------------------------------------------------------------------------------------------------------------------------------------------------------------------------------------------------------------------------------------------------------------------------------------------------------------------------------------------------------------------------------------------------------

>KU532147 ac10

------------------------------------------------------------------------------------------------------------------------------------------------------------------------------------------------------------------------------------------------------------------------------------------------------------------------------------------------------------------------------------------------------------------------------------------------------------------------------------------------------------------------------------------------------------------------------------------------------------------------------------------------------------------------------------------------------------------------------------------------------------------------------------------------------------------------GTTTTGATTTTGCCTGCTTTTGGGATTGTTAGACAGTCTACTTTATATTTAACGGGTAAGAAGGAGGTTTTTGGTTATTTGGGTATGGTTTATGCTATTTTAAGGATTGGTTTGATTGGTTGTGTGGTTTGGGCTCATCATATATATACGGTTGGTATGGATTTAGATTCTCGTGCTTATTTTACTGCTGCTACTATAGTTATTGCGGTTCCTACGGGAGTGAAGGTTTTTAGGTGGTTAGCAACTTTATTTGGTATAAAGATATTGTTTCAACCTATTTTATTGTGGGTTTTGGGTTTTATT---TTTTTATTTACTATTGGGGGGTTAACTGGGGTTATATTGTCTAATTCTAGTTTGGATATTATTTTACATGATACTTATTATGTAGTTAGGCATTTTCATTATGTTTTAAGGTTGGGGGCTGTTTTTGGTATTTTTACTGGTATTAGTCTTTGATGAAGGTTTATGACTGGTTGTGTTTATAATAAGTTGTATATAGTGGTTATGTTTTCTTTGATGTTTTTAGGTGTGAATTTGACTTTTTTTCCTTTACATTTT---------------------------------------------------------------------------------------------------------------------------------------------------------------------------------------------------------------------------------------------------------------------------

>KU532143 ac11

------------------------------------------------------------------------------------------------------------------------------------------------------------------------------------------------------------------------------------------------------------------------------------------------------------------------------------------------------------------------------------------------------------------------------------------------------------------------------------------------------------------------------------------------------------------------------------------------------------------------------------------------------------------------------------------------------------------------------------------------------------------------------------------------------------------------GTTTTGATTTTGCCTGCTTTTGGGATTGTTAGGCAGTCTACTTTATATTTAACGGGTAAGAAAGAGGTTTTTGGTTATTTGGGTATGGTTTATGCTATTTTGAGGATTGGTTTGATTGGTTGTGTGGTTTGGGCTCATCATATATATACGGTTGGTATGGATTTAGATTCTCGTGCTTATTTTACTGCGGCTACTATAGTTATTGCGGTCCCTACGGGAGTGAAGGTTTTTAGGTGGTTAGCGACTTTATTTGGTATAAAGATATTGTTTCAACCTATTTTATTGTGGGTTTTAGGTTTTATT---TTTTTATTTACTATTGGAGGGTTAACGGGAGTTATATTGTCTAATTCTAGTTTGGATATTATTTTACATGATACTTATTATGTAGTTAGGCATTTTCATTATGTTTTAAGGTTGGGGGCTGTTTTTGGTATTTTTACTGGTATTAGTCTTTGATGAAGGTTCATGACTGGTTGTGTTTATAATAAGTTGTATATAGTGGTAATGTTTTTTTTGATGTTTTTAGGTGTGAATTTGACTTTTTTTCCTTTGCATTTT---------------------------------------------------------------------------------------------------------------------------------------------------------------------------------------------------------------------------------------------------------------------------

>KU532148 ac12

------------------------------------------------------------------------------------------------------------------------------------------------------------------------------------------------------------------------------------------------------------------------------------------------------------------------------------------------------------------------------------------------------------------------------------------------------------------------------------------------------------------------------------------------------------------------------------------------------------------------------------------------------------------------------------------------------------------------------------------------------------------------------------------------------------------------GTTTTGATTTTGCCTGCTTTTGGGATTGTTAGACAGTCTACTTTATATTTAACGGGTAAGAAGGAGGTTTTTGGTTATTTGGGTATGGTTTATGCTATTTTAAGGATTGGTTTGATTGGTTGTGTGGTTTGGGCCCATCATATATATACGGTTGGTATGGATTTAGATTCTCGTGCTTATTTTACTGCGGCTACCATAGTTATTGCGGTTCCTACGGGAGTGAAGGTTTTTAGGTGGTTAGCAACTTTATTTGGTATGAAGATATTGTTTCAACCTATTTTATTGTGGGTTTTGGGTTTTATT---TTTTTGTTTACTATTGGGGGGTTAACTGGGGTTATATTGTCTAATTCTAGTTTGGATATTATTTTACATGATACTTATTATGTAGTTAGGCATTTTCATTATGTTTTAAGGTTGGGGGCTGTTTTTGGTATTTTTACTGGTATCAGTCTTTGATGAAGGTTTATGACTGGTTGTGTTTATAATAAGTTGTATATAGTGGTTATGTTTTTTTTGATGTTTTTAGGTGTGAATTTGACTTTTTTTCCTTTGCATTTT---------------------------------------------------------------------------------------------------------------------------------------------------------------------------------------------------------------------------------------------------------------------------

>KU532146 ac13

------------------------------------------------------------------------------------------------------------------------------------------------------------------------------------------------------------------------------------------------------------------------------------------------------------------------------------------------------------------------------------------------------------------------------------------------------------------------------------------------------------------------------------------------------------------------------------------------------------------------------------------------------------------------------------------------------------------------------------------------------------------------------------------------------------------------GTTTTGATTTTGCCTGCTTTTGGGATTGTTAGACAGTCTACTTTATATTTAACGGGTAAGAAGGAGGTTTTTGGTTATTTGGGTATGGTTTATGCTATTTTAAGGATTGGTTTGATTGGTTGTGTGGTTTGGGCCCACCATATATACACGGTTGGTATGGATTTAGATTCTCGTGCTTATTTTACTGCGGCTACTATAGTTATTGCGGTTCCTACGGGAGTGAAGGTTTTTAGGTGGTTAGCAACTTTATTTGGTATGAAGATATTGTTTCAACCTATTTTATTGTGGGTTTTGGGTTTTATT---TTTTTGTTTACTATTGGGGGGTTGACTGGGGTTATATTGTCTAATTCTAGTTTGGATATTATTTTACATGATACTTATTATGTAGTTAGGCATTTTCATTATGTTTTGAGGTTGGGAGCTGTTTTTGGTATTTTTACTGGTATTAGTCTTTGATGAAGGTTTATGACTGGTTGTGCTTATAATAAGTTGTATATAGTGGTTATGTTTTTTTTGATGTTTTTAGGTGTGAATTTGACTTTTTTTCCTTTGCATTTT---------------------------------------------------------------------------------------------------------------------------------------------------------------------------------------------------------------------------------------------------------------------------

>KU532150 am2

------------------------------------------------------------------------------------------------------------------------------------------------------------------------------------------------------------------------------------------------------------------------------------------------------------------------------------------------------------------------------------------------------------------------------------------------------------------------------------------------------------------------------------------------------------------------------------------------------------------------------------------------------------------------------------------------------------------------------------------------------------------------------------------------------------------------GTTGAGATTTCGCCCGCATTTGGGATTGTTAGGCAATACACTGTGTATTTAACGGGTAAAAAAGAAGTTTTGGGTTATTTAGGTATAGTATATGCTATTTTAAGTATTGGTATAATTGGTTGTGTAGTGAGGGCTCATCATATATATACAGTAGGTATAGATTTAGATTCTCGTGCTTATTTTACTGCGGCTACGATGGTTATTGCGGTTCCTACAGGGGTAAAGGTGTTTAGATGATTAGCAACTTTGTTTGGTATAAAAATGTTATTTCAGCCTATTTTGTTGTGGGTTTTAGGTTTTATT---TTTTTATTTACTATTGGGGGTTTGACTGGGGTTATATTGTCTAATTCTAGTTTAGATATTATTTTACATGATACTTATTATGTAGTTAGGCATTTTCATTATGTTTTGAGGTTAGGGGCTGTTTTTGGTATTTTTACTGGTATTAGTCTTTGATGAAGTTTTATGACTGGTTACGTATATAATAAGTTATATATAGTAGTGATATTTTTTTTGATGTTTTTGGGGGTGAATTTGACTTTTTTTCC-------------------------------------------------------------------------------------------------------------------------------------------------------------------------------------------------------------------------------------------------------------------------------------

>KU532149 am3

------------------------------------------------------------------------------------------------------------------------------------------------------------------------------------------------------------------------------------------------------------------------------------------------------------------------------------------------------------------------------------------------------------------------------------------------------------------------------------------------------------------------------------------------------------------------------------------------------------------------------------------------------------------------------------------------------------------------------------------------------------------------------------------------------------------------GTCTTGATTTTGCCTGCATTTGGGATTGTTAGGCAATCTACTTTGTATTTAACGGGTAAAAAAGAAGTTTTTGGTTATTTAGGTATAGTTTATGCTATTTTAAGTATTGGTTTAATTGGTTGTGTAGTGTGGGCTCATCATATATATACAGTAGGTATAGATTTAGATTCTCGTGCTTATTTTACTGCGGCTACGATGGTTATTGCTGTTCCTACAGGGGTAAAGGTGTTTAGATGATTAGCAACTTTGTTTGGTATAAAAATGTTATTTCAGCCTATTTTGTTGTGGGTTTTAGGTTTTATT---TTTTTATTTACTATTGGGGGTTTGACTGGGGTTATATTGTCTAATTCTAGTTTAGATATTATTTTACATGATACTTATTATGTAGTTAGGCATTTTCATTATGTTTTGAGGTTAGGGGCTGTTTTTGGTATTTTTACTGGTATTAGTCTTTGATGAAGTTTTATGACTGGTTACGTATATAATAAGTTATATATAGTAGTGATATTTTTTTTGATGTTTTTGGGTGTGAATTTGACTTTTTTTCCTTTGCATTTT---------------------------------------------------------------------------------------------------------------------------------------------------------------------------------------------------------------------------------------------------------------------------

>KU532154 am4

------------------------------------------------------------------------------------------------------------------------------------------------------------------------------------------------------------------------------------------------------------------------------------------------------------------------------------------------------------------------------------------------------------------------------------------------------------------------------------------------------------------------------------------------------------------------------------------------------------------------------------------------------------------------------------------------------------------------------------------------------------------------------------------------------------------------GTCTTGATTTTGCCTGCATTTGGGATTGTTAGGCAATCTACTTTGTATTTAACGGGTAAAAAAGAAGTTTTTGGTTATTTAGGTATAGTTTATGCTATTTTAAGTATTGGTTTAATTGGTTGTGTAGTGTGGGCTCATCATATATATACAGTAGGTATAGATTTAGATTCTCGTGCTTATTTTACTGCGGCTACGATGGTTATTGCTGTTCCTACGGGGGTAAAGGTGTTTAGATGATTAGCAACTTTGTTTGGTATAAAAATGTTATTTCAGCCTATTTTGTTGTGGGTTTTAGGTTTTATT---TTTTTATTTACTATTGGGGGTTTGACTGGGGTTATATTGTCTAATTCTAGTTTAGATATTATTTTACATGATACTTATTATGTAGTTAGGCATTTTCATTATGTTTTGAGGTTAGGGGCTGTTTTTGGTATTTTTACTGGTATTAGTCTTTGATGAAGTTTTATGACTGGTTACGTATATAATAAGTTATATATAGTAGTGATATTTTTTTTGATGTTTTTGGGTGTGAATTTGACTTTTTTTCCTTTGCATTTT---------------------------------------------------------------------------------------------------------------------------------------------------------------------------------------------------------------------------------------------------------------------------

>MF000735

---------------------------------------------------------------------------------------AGTTCTAATCATAAAGATATTGGTACTCTTTATTTTTTGTTTGGTTTGTGGTCGGGTATGTTAGGTACTGCTTTATCTTTGATTGTTCGTTTGGAGTTATCTAAGCCTGGAATGTTGTTGTCTAATGGGCAATTGTATAATTCAATTATTACGGCTCATGCTTTTTTGATAATTTTTTTTATGGTAATGCCTAGTATGATTGGTGGTTTTGGTAATTGAATGTTGCCTTTGATGTTGGGGGCTCCAGATATGAGTTTTCCTCGTTTGAATAATTTGAGTTTTTGATTGTTGCCAACTTCGATGTTTTTGATTTTAGATTCTTGTTTTGTAGATATAGGATGTGGAACTAGTTGGACTGTTTATCCACCTTTGAGGAGTTTAGGTCATCCTGGTAGAAGGGTAGATTTGGCGATTTTTAGTTTGCATTGTGCTGGATTGAGTTCTATTTTGGGGGGTATTAATTTTATGTGTACGACGAAGAATATACGAAGAAGTTCAATTTCTTTGGAACATATAAGTTTGTTTGTTTGATCTGTTTTTGTAACTGTTTTTTTGTTGGTATTGTCTTTACCTGTGTTGGCGGGGGCTATTACCATGTTATTAACTGATCGTAATTTAAATACTTCTTTTTTTGATCCTAGTTCTGGTGGTAATCCTTTGATTTATCAGCATTTGTTTTGATTTTTTGGTCATCCTGAGGTTTA-------------------------------------------------------------------------------------------------------------------------------------------------------------------------------------------------------------------------------------------------------------------------------------------------------------------------------------------------------------------------------------------------------------------------------------------------------------------------------------------------------------------------------------------------------------------------------------------------------------------------------------------------------------------------------------------------------------------------------------------------------------------------------------------------------------------------------------------------------------

>MF000736

---------------------------------------------------------------------------------------AGTTCTAATCATAAAGATATTGGTACTCTTTATTTTTTGTTTGGTTTGTGGTCGGGTATGTTAGGTACTGCTTTATCTTTGATTGTTCGTTTGGAGTTATCTAAGCCTGGAATGTTGTTGTCTAATGGGCAATTGTATAATTCAATTATTACGGCTCATGCTTTTTTGATAATTTTTTTTATGGTAATGCCTAGTATGATTGGTGGTTTTGGTAATTGAATGTTGCCTTTGATGTTGGGGGCTCCAGATATGAGTTTTCCTCGTTTGAATAATTTGAGTTTTTGATTGTTGCCAACTTCGATGTTTTTGATTTTAGATTCTTGTTTTGTAGATATGGGATGTGGAACTAGTTGGACTGTTTATCCACCTTTGAGGAGTTTAGGTCATCCTGGTAGAAGGGTAGATTTGGCGATTTTTAGTTTGCATTGTGCTGGATTGAGTTCTATTTTGGGGGGTATTAATTTTATGTGTACGACGAAGAATATGCGAAGAAGTTCAATTTCTTTGGAACATATAAGTTTGTTTGTTTGATCTGTTTTTGTAACTGTTTTTTTGTTGGTATTGTCTTTACCTGTGTTGGCGGGGGCTATTACCATGTTATTAACTGATCGTAATTTAAATACTTCTTTTTTTGATCCTAGTTCTGGTGGTAATCCTTTGATTTATCAGCATTTGTTTTGATTTTTTGGTCATCCTGAGGTTTA-------------------------------------------------------------------------------------------------------------------------------------------------------------------------------------------------------------------------------------------------------------------------------------------------------------------------------------------------------------------------------------------------------------------------------------------------------------------------------------------------------------------------------------------------------------------------------------------------------------------------------------------------------------------------------------------------------------------------------------------------------------------------------------------------------------------------------------------------------------

>KU934244

-------------------------------------------------------------------------------------------------------------------------------------------------------------------------------------------------------------------------------------------------------------------------------------------------------------------------------------------------------------------------------------------------------------------------------------------------------------------------------------------------------------------------------------------------------------------------------------------------------------------------------------------------------------------------------------------------------------------------------------------------------------------------------------------------------------------------------------------------------------------------------------------------------------------ATGCTATTTTAAGGATTGGTTTGATTGGTTGTGTGGTTTGGGCTCATCATATATATACGGTTGGTATGGATTTAGATTCTCGTGCTTATTTTACTGCTGCTACTATAGTTATTGCGGTTCCTACGGGAGTGAAGGTTTTTARGTGGTTAGCAACTTTATTTGGTATAAAAATATTGTTTCAACCTATTTTATTGTGGGTTTTGGGTTTTATT---TTTTTATTTACTATTGGGGGGTTAACTGGGGTTATATTGTCTAATTCTAGTT--------------------------------------------------------------------------------------------------------------------------------------------------------------------------------------------------------------------------------------------------------------------------------------------------------------------------------------------------------------------------------------------------------------------------------------------------------------------------------------

>KU934245

-------------------------------------------------------------------------------------------------------------------------------------------------------------------------------------------------------------------------------------------------------------------------------------------------------------------------------------------------------------------------------------------------------------------------------------------------------------------------------------------------------------------------------------------------------------------------------------------------------------------------------------------------------------------------------------------------------------------------------------------------------------------------------------------------------------------------------------------------------------------------------------------------------------------ATGGCATTTTAAGGATTGGTTTGATCGGTTGTGTGGTTTGGGCTCATCATATATATACGGTTGGTATGGATTTAGATTCTCGTGCTTATTTTACTGCTGCTACTATAGTTATTGCGGTTCCTACGGGAGTGAAGGTTTTTAGGTGGTTAGCAACTTTATTTGGTATAAAGATATTGTTTCAACCTATTTTATTGTGGGTTTTGGGTTTTATT---TTTTTATTTACTATTGGGGGGTTAACTGGGGTTATATTGTCTAATTCTAGTT--------------------------------------------------------------------------------------------------------------------------------------------------------------------------------------------------------------------------------------------------------------------------------------------------------------------------------------------------------------------------------------------------------------------------------------------------------------------------------------

>KU934237

-------------------------------------------------------------------------------------------------------------------------------------------------------------------------------------------------------------------------------------------------------------------------------------------------------------------------------------------------------------------------------------------------------------------------------------------------------------------------------------------------------------------------------------------------------------------------------------------------------------------------------------------------------------------------------------------------------------------------------------------------------------------------------------------------------------------------------------------------------------------------------------------------------------------ATGCTATTTTAAGTATTGGTTTAATTGGTTGTGTAGTGTGGGCTCATCATATATATACAGTAGGTATAGATTTAGATTCTCGTGCTTATTTTACTGCGGCTACGATGGTTATTGCTGTTCCTACAGGGGTAAAGGTGTTTAGATGATTAGCAACTTTGTTTGGTATAAAAATGTTATTTCAGCCTATTTTGTTGTGGGTTTTAGGTTTTATT---TTTTTATTTACTATTGGGGGTTTCACTGGGGTTATATTGTCTAATTCGAGTT--------------------------------------------------------------------------------------------------------------------------------------------------------------------------------------------------------------------------------------------------------------------------------------------------------------------------------------------------------------------------------------------------------------------------------------------------------------------------------------

>KU934238

-------------------------------------------------------------------------------------------------------------------------------------------------------------------------------------------------------------------------------------------------------------------------------------------------------------------------------------------------------------------------------------------------------------------------------------------------------------------------------------------------------------------------------------------------------------------------------------------------------------------------------------------------------------------------------------------------------------------------------------------------------------------------------------------------------------------------------------------------------------------------------------------------------------------ATGCTATTTTAAGTATTGGTTTAATTGGTTGTGTAGTATGGGCTCATCATATATATACAGTAGGTATAGATTTAGATTCTCGTGCTTATTTTACTGCGGCTACGATGGTTATTGCTGTTCCTACAGGGGTAAAGGTGTTTAGATGATTAGCAACTTTGTTTGGTATAAAAATGTTATTTCAGCCTATTTTGTTGTGGGTTTTAGGTTTTATT---TTTTTATTTACTATTGGGGGTTTGACTGGGGTTATATTGTCTAATTCTAGTT--------------------------------------------------------------------------------------------------------------------------------------------------------------------------------------------------------------------------------------------------------------------------------------------------------------------------------------------------------------------------------------------------------------------------------------------------------------------------------------

>KU934241

-------------------------------------------------------------------------------------------------------------------------------------------------------------------------------------------------------------------------------------------------------------------------------------------------------------------------------------------------------------------------------------------------------------------------------------------------------------------------------------------------------------------------------------------------------------------------------------------------------------------------------------------------------------------------------------------------------------------------------------------------------------------------------------------------------------------------------------------------------------------------------------------------------------------ATGCTATTTTAAGGATTGGTTTGATTGGTTGTGTGGTTTGGGCTCATCATATATATACAGGAGGTATAGATTTAGATTCTCGTGCTTATTTTACTGCGGCTACGATGGTTATTGCTGTTCCTACAGGGGTAAAGGTGTTTAGATGGTTAGCAACTTTGTTTGGTATAAAAATGTTATTTCAACCTATTTTGTTGTGGGTTTTAGGTTTTATT---TTTTTATTTACTATTGGGGGTTTGACTGGGGTTATATTGTCTAATTCTAGTT--------------------------------------------------------------------------------------------------------------------------------------------------------------------------------------------------------------------------------------------------------------------------------------------------------------------------------------------------------------------------------------------------------------------------------------------------------------------------------------

>KU934242

-------------------------------------------------------------------------------------------------------------------------------------------------------------------------------------------------------------------------------------------------------------------------------------------------------------------------------------------------------------------------------------------------------------------------------------------------------------------------------------------------------------------------------------------------------------------------------------------------------------------------------------------------------------------------------------------------------------------------------------------------------------------------------------------------------------------------------------------------------------------------------------------------------------------ATGCTATTTTAAGGATTGGTTTGATTGGTTGTGTAGTTTGGGCTCATCATATATATACAGTAGGTATAGATTTAGATTCTCGTGCTTATTTTACTGCGGCTACGATGGTTATTGCTGTTCCTACAGGGGTAAAGGTGTTTAGATGATTAGCAACTTTGTTTGGTATAAAAATGTTATTTCAGCCTATTTTGTTGTGGGTTTTAGGTTTTATT---TTTTTATTTACTATTGGGGGTTTGACTGGGGTTATATTGTCTAATTCTAGTT--------------------------------------------------------------------------------------------------------------------------------------------------------------------------------------------------------------------------------------------------------------------------------------------------------------------------------------------------------------------------------------------------------------------------------------------------------------------------------------

>KU934246

-------------------------------------------------------------------------------------------------------------------------------------------------------------------------------------------------------------------------------------------------------------------------------------------------------------------------------------------------------------------------------------------------------------------------------------------------------------------------------------------------------------------------------------------------------------------------------------------------------------------------------------------------------------------------------------------------------------------------------------------------------------------------------------------------------------------------------------------------------------------------------------------------------------------ATGCTATTTTAAGTATTGGTTTAATTGGTTGTGTAGTGTGGGCTCATCATATATATACAGTAGGTATAGATTTAGATTCTCGTGCTTATTTTACTGCGGCTACGATGGTTATTGCTGTTCCTACAGGGGTAAAGGTGTTTAGATGATTAGCAACTTTGTTTGGTATAAAAATGTTATTTCAGCCTATTTTGTTGTGGGTTTTAGGTTTCATT---TTTTTATTTACTATTGGGGGTTTGACTGGAGTTATATTGTCTAATTCTAGTT--------------------------------------------------------------------------------------------------------------------------------------------------------------------------------------------------------------------------------------------------------------------------------------------------------------------------------------------------------------------------------------------------------------------------------------------------------------------------------------

>AB699589

----------------------------------------------------------------------------------------------------------------------------------------------------------------------------------------------------------------------------------------------------------------------------------------------------------------------------------------------------------------------------------------------------------------------------------------------------------------------------------------------------------------------------------------------------------------------------------------------------------------------------------------------------------------------------------------------------------------------------------------------------------------------------------------------------------------------------------------------------------------------------CGGGTAAGAAGGAGGTTTTTGGTTATTTGGGTATGGTTTATGCTATTTTAAGGATTGGTTTGATTGGTTGTGTGGTTTGGGCTCACCATATATACACGGTTGGTATGGATTTAGATTCTCGTGCTTATTTTACTGCGGCTACTATAGTTATTGCGGTTCCTACGGGAGTGAAGGTTTTTAGGTGGTTAGCAACTTTATTTGGTATGAAGATATTGTTTCAACCTATTTTATTGTGGGTTTTGGGTTTTATT---TTTTTGTTTACTATTGGGGGGTTGACTGGGGTTATGTTGTCTAA----------------------------------------------------------------------------------------------------------------------------------------------------------------------------------------------------------------------------------------------------------------------------------------------------------------------------------------------------------------------------------------------------------------------------------------------------------------------------------------------

>KY439008 am9

-----------------------------------------------------------------------------------------------------------------------------------------------------------------------------------------------------------------------------------------------------------------------------------------------------------------------------------------------------------------------------------------------------------------------------------------------------------------------------------------------------------------------------------------------------------------------------------------------------------------------------------------------------------------------------------------------------------------------------------------------------------------------------------------------------------------------------------------------------------ATCTACTTTGTATTTAACGGGTAAAAAAGAAGTTTTTGGTTATTTAAAAATAGTTTATGCTATTTTAAGTATTGGTTTAATGGGTTGTGTAGTGTGGGCCCATCAAATATATACAGTAGGTATAGATTTAGATTCTCGTGCTTATTTTACTGCGGCTACGATGGTTATTGCTGTTCCTACAGGGGTAAAGGTGTTTAGATGATTAGCAACTTTGTTTGGTATAAAAATGTTATTCCAGCCTATTCTGTTCTGGAAATTAGGTTTTATT---TTTTTATTAACTATAGGGGGTTCGACTGGGGTTATATTGTCTAATTCTA-----------------------------------------------------------------------------------------------------------------------------------------------------------------------------------------------------------------------------------------------------------------------------------------------------------------------------------------------------------------------------------------------------------------------------------------------------------------------------------------

>KY439009 am7

-----------------------------------------------------------------------------------------------------------------------------------------------------------------------------------------------------------------------------------------------------------------------------------------------------------------------------------------------------------------------------------------------------------------------------------------------------------------------------------------------------------------------------------------------------------------------------------------------------------------------------------------------------------------------------------------------------------------------------------------------------------------------------------------------------------------------------------------------------------ATCTACTTTGTATTTAACGGGTAAAAAAGAAGTTTTTGGTTATTTAGGTATAGTTTATGCTATTTTAAGTATTGGTTTAATTGGTTGTGTAGTGTGGGCTCATCATATATATACAGTAGGTATAGATTTAGATTCTCGTGCTTATTTTACTGCGGCTACGATGGTTATTGCTGTTCCTACAGGGGTAAAGGTGTTTAGATGATTAGCAACTTTGTTTGGTATCAAAATGTTATTTCAGCCTATTTTGTTGTGGGTTTTAGGTTTCATT---TTTTTATTTACTATTGGGGGCTTGACTGGGGTTATATTGTCTAATTCTA-----------------------------------------------------------------------------------------------------------------------------------------------------------------------------------------------------------------------------------------------------------------------------------------------------------------------------------------------------------------------------------------------------------------------------------------------------------------------------------------

>KY439010 am8

-----------------------------------------------------------------------------------------------------------------------------------------------------------------------------------------------------------------------------------------------------------------------------------------------------------------------------------------------------------------------------------------------------------------------------------------------------------------------------------------------------------------------------------------------------------------------------------------------------------------------------------------------------------------------------------------------------------------------------------------------------------------------------------------------------------------------------------------------------------ATCTACTTTGTATTTAACGGGTAAAAAAGAAGTTTTTGGTTATTTAGGTATAGTTTATGCTATTTTAAGTATTGGTTTAATTGGTTGTGTAGTGTGGGCTCATCATATATATACAGTAGGTATAGATTTAGATTCTCGTGCTTATTTTACTGCGGCTACGATGGTTATTGCTGTTCCTACAGGGGTAAAGGTGTTTAGATGATTAGCAACTTTGTTTGGTATAAAAATGTTATTTCAGCCTATTTTGTTGTGGGTTTTAGGTTTCATT---TTTTTATTTACTATTGGGGGTCTGACTGGGGTTATATTGTCTAATTCTA-----------------------------------------------------------------------------------------------------------------------------------------------------------------------------------------------------------------------------------------------------------------------------------------------------------------------------------------------------------------------------------------------------------------------------------------------------------------------------------------

>KY439011 am6

-----------------------------------------------------------------------------------------------------------------------------------------------------------------------------------------------------------------------------------------------------------------------------------------------------------------------------------------------------------------------------------------------------------------------------------------------------------------------------------------------------------------------------------------------------------------------------------------------------------------------------------------------------------------------------------------------------------------------------------------------------------------------------------------------------------------------------------------------------------ACCTACTTTGTATTTAACGGGCAAAAACGAAGTTTTTGGATATTTAGGTATAGTTTATGCTATTTTAAGTATTGGTTTAATTGGTTGTGTAGTGTGGGCTCATCATATATATACAGTAGGCATAGATTTAGATTCTCGGGCTTATTTTACTGCGGCTACGATGCTTATTGCTGTTCCTACAGGGGTAAAGGTGTTTAGATGATTAGCAACTTTGTTTGGCCCAAAAATGTTATTTCAGCCTATTTTGTTGTGGGATTTAGGATTCATT---TTTTTATTTACTATTGGGGGCTTGACTACCCTTATATTGTCTAATTCTA-----------------------------------------------------------------------------------------------------------------------------------------------------------------------------------------------------------------------------------------------------------------------------------------------------------------------------------------------------------------------------------------------------------------------------------------------------------------------------------------

>KY439012 am5

-----------------------------------------------------------------------------------------------------------------------------------------------------------------------------------------------------------------------------------------------------------------------------------------------------------------------------------------------------------------------------------------------------------------------------------------------------------------------------------------------------------------------------------------------------------------------------------------------------------------------------------------------------------------------------------------------------------------------------------------------------------------------------------------------------------------------------------------------------------ATCTACTTTGTATTTAACGGGTAAAAAAGAAGTTTTTGGTTATTTAGGTATAGTTTATGCTATTTTAAGTATTGGTTTAATTGGTTGTGTAGTGTGGGCTCATCATATATATACAGTAGGTATAGATTTAGATTCCCGTGCTTATCTTACTGCGGCTACGAAGCTTATTGCTGTTCCTACAGGGAAACAGGTGTTTAGATGACTAGCAACTTTGTATGAGCCCAAACTGTTATTTCAGCCTATTTTGTTGTTAGTTTTAGGTTTCATT---TCTTTATTTACTATTGAGGGCTTTACTGCCGTTATAATACCCAATTTTT-----------------------------------------------------------------------------------------------------------------------------------------------------------------------------------------------------------------------------------------------------------------------------------------------------------------------------------------------------------------------------------------------------------------------------------------------------------------------------------------

>Aghazadeh A. mackerasae

---------------------------------------ATTTATATATATAAGAAATATCAAAGTGGTTTGTCGGTTTGGTTAGAAAGTTCTAACCATAAGGATATTGGTACTCTTTATTTTTTGTTTGGTTTGTGGTCGGGTATGTTAGGTACTGCTTTATCTTTGATTGTTCGTTTGGAGTTATCTAAGCCTGGAATGTTGTTGTCTAATGGGCAATTGTATAATTCAATTATTACGGCTCATGCTTTTTTGATAATTTTTTTTATGGTAATGCCTAGTATGATTGGTGGTTTTGGTAATTGAATGTTGCCTTTGATGTTGGGGGCTCCAGATATGAGTTTTCCTCGTTTGAATAATTTGAGTTTTTGATTGTTGCCAACTTCGATGTTTTTGATTTTAGATTCTTGTTTTGTAGATATAGGATGTGGAACTAGTTGGACTGTTTATCCACCTTTGAGGAGTTTAGGTCATCCTGGTAGAAGGGTAGATTTGGCGATTTTTAGTTTGCATTGTGCTGGATTGAGTTCTATTTTGGGGGGTGTTAATTTTATGTGTACGACGAAGAATATGCGAAGAAGTTCAATTTCTTTGGAACACATAAGTTTGTTTGTTTGATCTGTTTTTGTAACTGTTTTTTTGTTGGTATTGTCTTTACCTGTGTTGGCGGGGGCTATTACTATATTATTAACTGATCGTAATTTAAATACTTCTTTTTTTGACCCTAGTTCTGGTGGTAATCCTTTGATTTATCAGCATTTGTTTTGATTTTTTGGTCACCCTGAGGTTTATGTTTTGATTTTGCCTGCTTTTGGGATTGTTAGACAGTCTACTTTATATTTAACGGGTAAGAAGGAGGTTTTTGGTTATTTGGGTATGGTTTATGCTATTTTAAGGATTGGTTTGATTGGTTGTGTGGTTTGGGCCCACCATATATACACGGTTGGTATGGATTTAGATTCTCGTGCTTATTTTACTGCGGCTACTATAGTTATTGCGGTTCCTACGGGAGTGAAGGTTTTTAGGTGGTTAGCAACTTTATTTGGTATGAAGATATTGTTTCAACCTATTTTATTGTGGGTTTTGGGTTTTATT---TTTTTGTTTACTATTGGGGGGTTGACTGGGGTTATATTGTCTAATTCTAGTTTGGATATTATTTTACATGATACTTATTATGTAGTTAGGCATTTTCATTATGTTTTGAGGTTGGGAGCTGTTTTTGGTATTTTTACTGGTATTAGTCTTTGATGAAGGTTTATGACTGGTTGTGTTTATAATAAGTTGTATATAGTAGTTATGTTTTTTTTGATGTTTTTAGGTGTGAATTTGACTTTTTTTCCTTTGCATTTTGCTGGTTTGCACGGTTATCCTCGTAAGTATTTGGATTATCCTGATGTTTATTCGGTTTGAAATGTTATTTCTTCTTTTGGGTCTTTAGTTAGTGTTTTTGCTTTATTTATGTTTATTTTTTTGTTGTTGGAGTCTTTTTTTAGATATCGTTTGTTGTTATTGGATAATTATTATAATAGAAGTCCAGAATATAGATATAGGAGTTATGTTTTTGGTCATAGCTATCAGTCGGAAGTTTATTTTAGAAGAAGGAGTTTAAAGTGTTAA

>KY439004 ac10x

-----------------------------------------------------------------------------------------------------------------------------------------------------------------------------------------------------------------------------------------------------------------------------------------------------------------------------------------------------------------------------------------------------------------------------------------------------------------------------------------------------------------------------------------------------------------------------------------------------------------------------------------------------------------------------------------------------------------------------------------------------------------------------------------------------------------------------------------------------TATACAGTCTACTTTATATTTAACGGGTAAGAAGGAGGTTTTTGGTTATTTGGGTATGGTTTATGCTATTTTAAGGATTGGTTTGATTGGTTGTGTGGTTTGGGCTCATCATATATATACGGTTGGTATGGATTTAGATTCTCGTGCTTATTTTACTGCTGCTACTATAGTTATTGCGGTTCCTACGGGAGTGAAGGTTTTTAGGTGGTTAGCAACTTTATTTGGTATAAAGATATTGTTTCAACCTATTTTATTGTGGGTTTTGGGTTTTATT---TTTTTATTTACTATTGGGGGGTTAACTGGGGTTATATTGTCTAATTCTAGTTTGGATATTATTTTACATGATACTTATTATGTAG-----------------------------------------------------------------------------------------------------------------------------------------------------------------------------------------------------------------------------------------------------------------------------------------------------------------------------------------------------------------------------------------------------------------------------------------------------

>KY439007 ac14

-----------------------------------------------------------------------------------------------------------------------------------------------------------------------------------------------------------------------------------------------------------------------------------------------------------------------------------------------------------------------------------------------------------------------------------------------------------------------------------------------------------------------------------------------------------------------------------------------------------------------------------------------------------------------------------------------------------------------------------------------------------------------------------------------------------------------------------------------------TAGACAGTCTACTTTATATTTAACGGGTAAGAAGGAGGTTTTTGGTTATTTGGGTATGGTTTACGCTATTTTAAGGATTGGTTTGATTGGTTGTGTGGTTTGGGCTCATCATATATATACGGTTGGTATGGATTTAGATTCTCGTGCTTATTTTACTGCGGCTACTATAGTTATTGCGGTTCCTACGGGAGTGAAGGTTTTTAGGTGGTTAGCAACTTTATTTGGTATAAAGATATTGTTTCAACCTATTTTATTGTGGGTTTTGGGTTTTATT---TTTTTATTTACTATTGGGGGGTTAACTGGGGTTATATTGTCTAATTCTAGTTTGGATATTGTGTACTATGACACCCCTCAGGTCT-----------------------------------------------------------------------------------------------------------------------------------------------------------------------------------------------------------------------------------------------------------------------------------------------------------------------------------------------------------------------------------------------------------------------------------------------------

>KY703435 ac15

------------------------------------------------------------------------------------------------------------------------------------------------------------------------------------------------------------------------------------------------------------------------------------------------------------------------------------------------------------------------------------------------------------------------------------------------------------------------------------------------------------------------------------------------------------------------------------------------------------------------------------------------------------------------------------------------------------------------------------------------------------------------------------------------------------------------------------------------------------TCTACTTTATATTTAACGGGTAAGAAGGAGGTTTTTGGTTATTTGGGTATGGTTTATGCTATTTTAAGGATTGGTTTGATTGGTTGTGTGGTTTGGGCTCATCATATATATACGGTTGGTATGGATTTAGATTCTCGTGCTTATTTTACTGCGGCTACTATAGTTATTGCGGTTCCTACTGGAGTGAAGGTTTTTAGGTGGTTAGCAACTTTATTTGGTATAAAGATATTGTTTCAGCCTATTTTATTGTGGGTTTTGGGTTTTATT---TTTTTATTTACTATTGGGGGGTTGACTGGGGTTATATTGTCTAATTCTAGTTTGGATATTATTTTACATGATACTTATTATGTAG-----------------------------------------------------------------------------------------------------------------------------------------------------------------------------------------------------------------------------------------------------------------------------------------------------------------------------------------------------------------------------------------------------------------------------------------------------

>KY703436 ac16

------------------------------------------------------------------------------------------------------------------------------------------------------------------------------------------------------------------------------------------------------------------------------------------------------------------------------------------------------------------------------------------------------------------------------------------------------------------------------------------------------------------------------------------------------------------------------------------------------------------------------------------------------------------------------------------------------------------------------------------------------------------------------------------------------------------------------------------------------------TCTACTTTATATTTAACGGGTAAGAAGGAGGTTTTTGGTTATTTGGGTATGGTTTATGCTATTTTAAGGATTGGTTTGATTGGTTGTGTGGTTTGGGCTCATCATATATATACGGTTGGTATGGATTTAGATTCTCGTGCTTATTTTACTGCGGCTACTATAGTTATTGCGGTTCCTACGGGAGTAAAGGTTTTTAGGTGGTTGGCAACTTTATTTGGTATAAAGATATTGTTTCAGCCTATTTTATTGTGGGTTTTGGGTTTTATT---TTTTTATTTACTATTGGGGGGTTGACTGGGGTTATATTGTCTAATTCTAGTTTGGATATTATTTTACATGATACTTATTATGTAG-----------------------------------------------------------------------------------------------------------------------------------------------------------------------------------------------------------------------------------------------------------------------------------------------------------------------------------------------------------------------------------------------------------------------------------------------------
