# Supplementary material for: Hurdles in the evolutionary epidemiology of Angiostrongylus cantonensis: Pseudogenes, incongruence between taxonomy and DNA sequence variants, and cryptic lineages
Source: Evol Appl. 2018 Mar 25;11(8):1257–69. doi: 10.1111/eva.12621 (PMC6099809; doi:10.1111/eva.12621)
Supplement: Supplementary file 6 [file EVA-11-1257-s006.docx]

>AY295804.1 Angiostrongylus cantonensis 18S small subunit ribosomal RNA gene, partial sequence

ATTAAGCCATGCATGAGGAGTTCAGCTTTAAGTGAAACTGCGAACGGCTCATTAGAGCAGATGTGATTTATTCGGAAAATCCTATTGGATAACTGCGGTAATTCTGGAGCTAATACATGCGTATAAACCCTGACTTTCGAAAGGGTGCAATTATTAGAGCAAATCAATCATTTTCGGATGTAGTTTGCTGACTCTGAATAACGCAGCATATCGGCGGCTTGTTCGCCGATAATCCGAAAAAGTGTCTGCCCTATCAACCTGATGGTAGTCTATTAGTCTACCATGGTTATTACGGGTAACGGAGAATAAGGGTTCGACTCCGGAGAGGGAGCCTTAGAAACGGCTACCACATCCAAGGAAGGCAGCAGGCGCGAAACTTATCCAATCTTGAATAGATGAGATAGTGACTAAAAATAAAAAGACCATTCCTATGGAACGGTTATTTCAATGAGTTGATCATAAACCTTTTTTCGAGTATCCAGTGGAGGGCAAGTCTGGTGCCAGCAGCCGCGGTAATTCCAGCTCCACTAGTGTAAATCGTCATTGCTGCGGTTAAAAAGCTCGTAGTTGGATCTGAGTTGCATGCAATGATTCGCCTTTGGCGTTAATCATTGTTGTGACTATTTGCTGGTTTTCTATTGAAATTTCGATTTCTTTAGTGGCTAGCGAGTTTACTTTGAATAAATTAAAGTGCTCAGAACAAGCGTTTGCTTGAATGGTCGATCATGGAATAATAAAAGAGGACTTCGGTTCTATTTATTGGTTCAGGAACTGAAGTAATGATTAAGAGGGACAATTCGGGGGCATTCGTATCCCTGCGCGAGAGGTGAAATTCGTGGACCGCAGGGGGACGCCCTAAAGCGAAAGCATTTGCCAAGAATGTCTTCATTAATCAAGAACGAAAGTCAGAGGTTCGAAGGCGATTAGATACCGCCCTAGTTCTGACCGTAAACTATGCCATCTAGCGATCCGATGGGGTATTGTTGCCTTGTCGAGGAGCTTCCCGGAAACGAAAGTCTTTCGGTTCCTGGGGTAGTATGGTTGCAAAGCTGAAACTTAAAGAAATTGACGGAATGGCACCACCAGGAGTGGAGCCTGCGGCTTAATTTGACTCAACACGGGAAAACTCACCCGGCCCGGACACCGTAAGGATTGACAGATTGAAAGCTCTTTCTCGATTTGGTGGTTGGTGGTGCATGGCCGTTCTTAGTTGGTGGAGCGATTTGTCTGGTTTATTCCGATAACGAGCGAGACTCTAGCCTGCTAAATAGTGACTAGATTATTGAGTCTAGTCTACTTCTTAGAGGGATAAGCGGTGTTTAGCCGCACGAGATTGAGCGATAACAGGTCTGTGATGCCCTTAGATGTCCGGGGCTGCACGCGCGCTACAATGGAAGAATCAGCTGGCCTATCCATTGCCGAAAGGTATTGGTAAACCGTTGAAACTCTTCCGTGACCGGGATAGGGAATTGTAATTATTTCCCTTGAACGAGGAATTCCTAGTAAGTGTGAGTCATCAGCTCACGCTGATTACGTCCCTGCCATTTGTACACACCGCCCGTCGCTGTCCGGGACTGAGCTGTCTCGAGAGGACTGCGGACTACTGTATTGAGGCCTTCGGGTCGCGATATGGCGGGAAACAGTTCAATCGCAATGGCTTGAACCGGGTAAAAGTCGTAACAAGGTATCTG

>KU528683.1 Angiostrongylus cantonensis isolate Ac39L3SIE 18S ribosomal RNA gene, partial sequence

---------------AGGAGTTCAGCTTTAAGTGAAACTGCGAACGGCTCATTAGAGCAGATGTGATTTATTCGGAAAATCCTATTGGATAACTGCGGTAATTCTGGAGCTAATACATGCGTATAAACCCTGACTTTCGAAAGGGTGCAATTATTAGAGCAAATCAATCATTTTCGGATGTAGTTTGCTGACTCTGAATAACGCAGCATATCGGCGGCTTGTTCGCCGATAATCCGAAAAAGTGTCTGCCCTATCAACCTGATGGTAGTCTATTAGTCTACCATGGTTATTACGGGTAACGGAGAATAAGGGTTCGACTCCGGAGAGGGAGCCTTAGAAACGGCTACCACATCCAAGGAAGGCAGCAGGCGCGAAACTTATCCAATCTTGAATAGATGAGATAGTGACTAAAAATAAAAAGACCATTCCTATGGAACGGTTATTTCAATGAGTTGATCATAAACCTTTTTTCGAGTATCCAGTGGAGGGCAAGTCTGGTGCCAGCAGCCGCGGTAATTCCAGCTCCACTAGTGTAAATCGTCATTGCTGCGGTTAAAAAGCTCGTAGTTGGATCTGAGTTGCATGCAATGATTCGCCTTTGGCGTTAATCATTGTTGTGACTATTTGCTGGTTTTCTATTGAAATTTCGATTTCTTTAGTGGCTAGCGAGTTTACTTTGAATAAATTAGAGTGCTCAGAACAAGCGTTTGCTTGAATGGTCGATCATGGAATAATAAAAGAGGACTTCGGTTCTATTTATTGGTTCAGGAACTGAAGTAATGATTAAGAGGGACAATTCGGGGGCATTCGTATCCCTGCGCGAGAGGTGAAATTCGTGGACCGCAGGGGGACGCCCTAAAG--------------------------------------------------------------------------------------------------------------------------------------------------------------------------------------------------------------------------------------------------------------------------------------------------------------------------------------------------------------------------------------------------------------------------------------------------------------------------------------------------------------------------------------------------------------------------------------------------------------------------------------------------------------------------------------------------------------------------------------------------------------------------------------------------------------------------------------------------------------

>EF514914.1 Angiostrongylus malaysiensis 18S ribosomal RNA gene, partial sequence

---------------AGGAGTTCAGCTTCAAGTGAAACTGCGAACGGCTCATTAGAGCAGATGTGATTTATTCGGAAAATCCTATTGGATAACTGCGGTAATTCTGGAGCTAATACATGCGTATAAACCCTGACTTTCGAAAGGGTGCAATTATTAGAGCAAATCAATCATTTTCGGATGCAGTTTGCTGACTCTGAATAACGCAGCATATCGGCGGCTTGTTCGCTGATAATCCGAAAAAGTGTCTGCCCTATCAACCTGATGGTAGTCTATTAGTCTACCATGGTTATTACGGGTAACGGAGAATAAGGGTTCGACTCCGGAGAGGGAGCCTTAGAAACGGCTACCACATCCAAGGAAGGCAGCAGGCGCGAAACTTATCCAATCTTGAATAGATGAGATAGTGACTAAAAATAAAAAGACCATTCCTATGGAACGGTTATTTCAATGAGTTGATCATAAACCTTTTTTCGAGTATCAAGTGGAGGGCAAGTCTGGTGCCAGCAGCCGCGGTAATTCCAGCTCCACTAGTGTAAATCGTCATTGCTGCGGTTAAAAAGCTCGTAGTTGGATCTGAGTTGCATGCAATGATTCGCCTTTGGCGTTAATCATTGTTGTGACTATTTGCTGGTTTTCTATTGAAATTTCGATTTCTCTAGTGACTGGCGAGTTTACTTTGAATAAATTAGAGTGCTCAGAACAAGCGTTTGCTTGAATGGTCGATCATGGAATAATAAAAGAGGACTTCGGTTCTATTTATTGGTTCAGGAACTGAAGTAATGATTAAGAGGGACAATTCGGGGGCATTCGTATCCCTGCGCGAGAGGTGAAATTCGTGGACCGCAGGGGGACGCCCTAAAGCGAAAGCATTTGCCAAGAATGTCTTCATTAATCAAGAACGAAAGTCAGAGGTTCGAAGGCGATTAGATACCGCCCTAGTTCTGACCGTAAACTATGCCATCTAGCGATCCGATGGGGTATTGTTGCCTTGTCGAGGAGCTTCCCGGAAACGAAAGTCTTTCGGTTCCTGGGGTAGTATGGTTGCAAAGCTGAAACTTAAAGAAATTGACGGAATGGCACCACCAGGAGTGGAGCCTGCGGCTTAATTTGACTCAACACGGGAAAACTCACCCGGCCCGGACACCGTAAGGATTGACAGATTGAAAGCTCTTTCTCGATTTGGTGGTTGGTGGTGCATGGCCGTTCTTAGTTGGTGGAGCGATTTGTCTGGTTTATTCCGATAACGAGCGAGACTCTAGCCTGCTAAATAGTGACTAGATTATTGAGTCTAGTCTACTTCTTAGAGGGATAAGCGGTGTTTAGCCGCACGAGATTGAGCGATAACAGGTCTGTGATGCCCTTAGATGTCCGGGGCTGCACGCGCGCTACAATGGAAGAATCAGCTGGCCTATCCATTGCCGAAAGGTATTGGTAAACCGTTGAAACTCTTCCGTGACCGGGATAGGGAATTGTAATTATTTCCCTTGAACGAGGAATTCCTAGTAAGTGTGAGTCATCAGCTCACGCTGATTACGTCCCTGCCATTTGTACACACCGCCCGTCGCTGTCCGGGACTGAGCTGTCTCGAGAGGACTGCGGACTACTGTATTGAGGCCTTCGGGTCGCAATATGGCGGGAAACAGTTCAATCGCAATGGCTTGAACCGGGTAAAAGTCGTAACAAGGTATCTG

>KU528687.1 Angiostrongylus malaysiensis isolate Am28L3VIE 18S ribosomal RNA gene, partial sequence

---------------AGGAGTTCAGCTTCAAGTGAAACTGCGAACGGCTCATTAGAGCAGATGTGATTTATTCGGAAAATCCTATTGGATAACTGCGGTAATTCTGGAGCTAATACATGCGTATAAACCCTGACTTTCGAAAGGGTGCAATTATTAGAGCAAATCAATCATTTTCGGATGTAGTTTGCTGACTCTGAATAACGCAGCATATCGGCGGCTTGTTCGCTGATAATCCGAAAAAGTGTCTGCCCTATCAACCTGATGGTAGTCTATTAGTCTACCATGGTTATTACGGGTAACGGAGAATAAGGGTTCGACTCCGGAGAGGGAGCCTTAGAAACGGCTACCACATCCAAGGAAGGCAGCAGGCGCGAAACTTATCCAATCTTGAATAGATGAGATAGTGACTAAAAATAAAAAGACCATTCCTATGGAACGGTTATTTCAATGAGTTGATCATAAACCTTTTTTCGAGTATCAAGTGGAGGGCAAGTCTGGTGCCAGCAGCCGCGGTAATTCCAGCTCCACTAGTGTAAATCGTCATTGCTGCGGTTAAAAAGCTCGTAGTTGGATCTGAGTTGCATGCAATGATTCGCCTTTGGCGTTAATCATTGTTGTGACTATTTGCTGGTTTTCTATTGAAATTTCGATTTCTCTAGTGGCTGGCGAGTTTACTTTGAATAAATTAGAGTGCTCAGAACAAGCGTTTGCTTGAATGGTCGATCATGGAATAATAAAAGAGGACTTCGGTTCTATTTATTGGTTCAGGAACTGAAGTAATGATTAAGAGGGACAATTCGGGGGCATTCGTATCCCTGCGCGAGAGGTGAAATTCGTGGACCGCA------------------------------------------------------------------------------------------------------------------------------------------------------------------------------------------------------------------------------------------------------------------------------------------------------------------------------------------------------------------------------------------------------------------------------------------------------------------------------------------------------------------------------------------------------------------------------------------------------------------------------------------------------------------------------------------------------------------------------------------------------------------------------------------------------------------------------------------------------------------------------
